# Thorium- and Uranium-Mediated C–H Activation of a Silyl-Substituted Cyclobutadienyl Ligand

**DOI:** 10.1021/acs.inorgchem.2c03534

**Published:** 2022-12-09

**Authors:** Nikolaos Tsoureas, Thayalan Rajeshkumar, Oliver P. E. Townrow, Laurent Maron, Richard A. Layfield

**Affiliations:** † Department of Chemistry, School of Life Sciences, University of Sussex, Brighton BN1 9QJ, U.K.; ‡ Laboratoire de Physique et Chimie des Nano-Objets, Institut National des Sciences Appliquées, Toulouse Cedex 4 31077, France; § Chemistry Research Laboratory, Department of Chemistry, University of Oxford, 12 Mansfield Road, Oxford OX1 3TA, U.K.

## Abstract

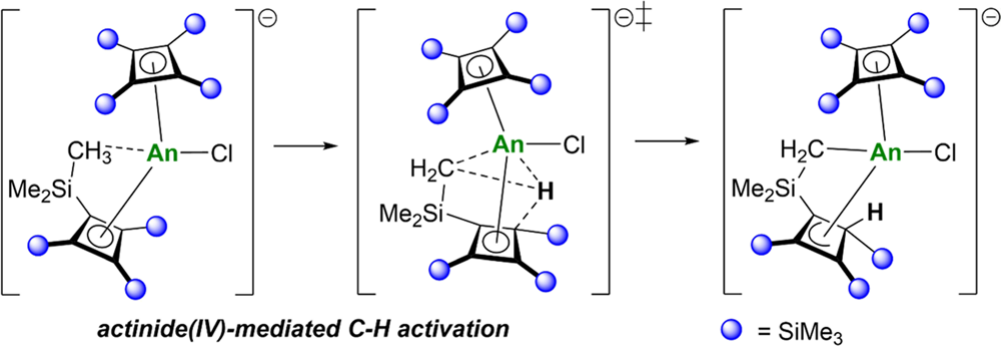

Cyclobutadienyl complexes of the f-elements are a relatively new yet poorly understood class of sandwich and half-sandwich organometallic compounds. We now describe cyclobutadienyl transfer reactions of the magnesium reagent [(η^4^-Cb'''')Mg(THF)_3_] (**1**), where Cb'''' is tetrakis(trimethylsilyl)cyclobutadienyl, toward thorium(IV) and uranium(IV) tetrachlorides. The 1:1 stoichiometric reactions between **1** and AnCl_4_ proceed with intact transfer of Cb'''' to give the half-sandwich complexes [(η^4^-Cb'''')AnCl(μ-Cl)_3_Mg(THF)_3_] (An = Th, **2**; An = U, **3**). Using a 2:1 reaction stoichiometry produces [Mg_2_Cl_3_(THF)_6_][(η^4^-Cb'''')An(η^3^-C_4_H(SiMe_3_)_3_-κ-(CH_2_SiMe_2_)(Cl)] (An = Th, [Mg_2_Cl_3_(THF)_6_][**4**]; An = U [Mg_2_Cl_3_(THF)_6_][**5**]), in which one Cb'''' ligand has undergone cyclometalation of a trimethylsilyl group, resulting in the formation of an An–C σ-bond, protonation of the four-membered ring, and an η^3^-allylic interaction with the actinide. Complex solution-phase dynamics are observed with multinuclear nuclear magnetic resonance spectroscopy for both sandwich complexes. A computational analysis of the reaction mechanism leading to the formation of **4** and **5** indicates that the cyclobutadienyl ligands undergo C–H activation across the actinide center.

## Abstract 2

Adding two equivalents of a bulky, trimethylsilyl-substituted cyclobutadienyl ligand as the magnesium salt to thorium(IV) or uranium(IV) chloride results in intact transfer of one ligand and activation of a methyl C−H bond across the actinide center on the second ligand, with subsequent formation of an η^3^-allylic cyclobutadienyl interaction.

## Introduction

Organometallic sandwich and half-sandwich compounds of the lanthanides and actinides have played a central role in the development of f-block chemistry. Applications of these compounds range from catalysis,^1−4^ small-molecule and other bond activation chemistry,^5−9^ and, more recently, single-molecule magnetism^10−13^ to fundamental questions related to electronic structure and chemical bonding.^14−18^ The most popular ligands in f-element organometallic chemistry are cyclopentadienyl (Cp),^19^ cyclo-octatetraenyl (COT),^20^ and their numerous substituted derivatives. This popularity stems largely from the availability of ligand precursors and the ease with which substituents can be introduced, which allows the steric, electronic, and solubility properties of their f-element complexes to be modified for a particular purpose. Other η-bonded ligands are less common in f-element chemistry, although several complexes of cycloheptatrienyl,^21^ arene,^22,23^ pentadienyl,^24,25^ and allyl^26^ ligands are known, and the cyclononatetraenyl ligand was recently introduced into lanthanide chemistry.^27,28^

Our interest in f-element sandwich compounds was recently extended to incorporate ligands based on the smaller, four-membered ring η^4^-cyclobutadienyl (Cb). In contrast to their cyclopentadienyl cousins, f-element complexes of cyclobutadienyl ligands are rare,^29,30^ primarily because very few cyclobutadiene ligand precursors are known owing to the instability associated with their antiaromatic character and ring strain. Indeed, the only stable cyclobutadiene derivatives that can be isolated in synthetically useful amounts are persilylated derivatives, notably C_4_(SiMe_3_)_4_ (Cb''''), first reported by Sekiguchi et al.^31,32^ Once isolated, Cb'''' can conveniently be converted into alkali metal salts of the type A_2_Cb'''' (A = Li, Na, or K), which, in principle, enable salt metathesis reactions with f-element halides and pseudohalides. However, in attempting to isolate sandwiches of the type [Ln(η^4^-Cb'''')_2_]^−^ and [U(η^4^-Cb'''')_2_], we have found that the [Cb'''']^2–^ ligand is prone to activation, either via deprotonation of a trimethylsilyl substituent to give cyclometalated complexes or by protonation to give complexes of the allylic derivative [η^3^-C_4_H(SiMe_3_)_3_-κ-(CH_2_SiMe_2_) (abbreviated to Cb'''(H)].^33^ Both modes of activation can also occur together.^34^ The mechanism through which the silyl-substituted cyclobutadienyl ligand undergoes cyclometalation and hydrogen atom transfer is not currently known. Despite the apparent reactivity of [Cb'''']^2–^ when exposed to f-elements, intact transfer of the ligand was achieved in the case of the lanthanide half-sandwich coordination polymers [A(μ:η^4^:η^4^-Cb'''')Ln(BH_4_)_2_(THF)]_∞_ (A = Na, K; Ln = Dy, Y),^35^ the uranium(IV) half-sandwich compounds [Na(12-crown-4)_2_][U(η^4^-Cb'''')(BH_4_)_3_] and [U(η^4^-Cb'''')(μ-BH_4_)_3_{K(THF)_2_}]_2_,^36,37^ and the hybrid uranocenes [(η^4^-Cb'''')U(η^8^-C_8_H_8_)] and [(η^4^-Cb'''')U{η^8^-1,4-(^i^Pr_3_Si)_2_C_8_H_6_}].^38^ An intriguing feature of these hybrid uranocenes is the apparent preference for overlap of the uranium 6d orbitals with the Cb'''' valence orbitals, whereas the 5f orbitals preferentially interact with the COT ligands.

The choice of lanthanide or actinide starting material seemingly plays an important role in determining the fate of the tetrakis(trimethylsilyl)cyclobutadienyl ligand in f-element chemistry. In addition to our observations, the reaction between K_2_Cb'''' and [ThCl_4_(THF)_3.5_] was reported to result in formation of neutral Cb'''' with concomitant reduction of thorium(IV) to thorium metal, which was interpreted in terms of the strongly reducing nature of [Cb'''']^2–^ toward the polar starting material.^39^ Intrigued by this, we were interested to determine if the less-polar magnesium reagent [(η^4^-Cb'''')Mg(THF)_3_] (**1**) would be more suitable in ligand transfer reactions toward the convenient starting materials thorium and uranium tetrachloride, as well as the mechanism(s) of any ligand activation processes.

## Results and Discussion

**Scheme 1:**
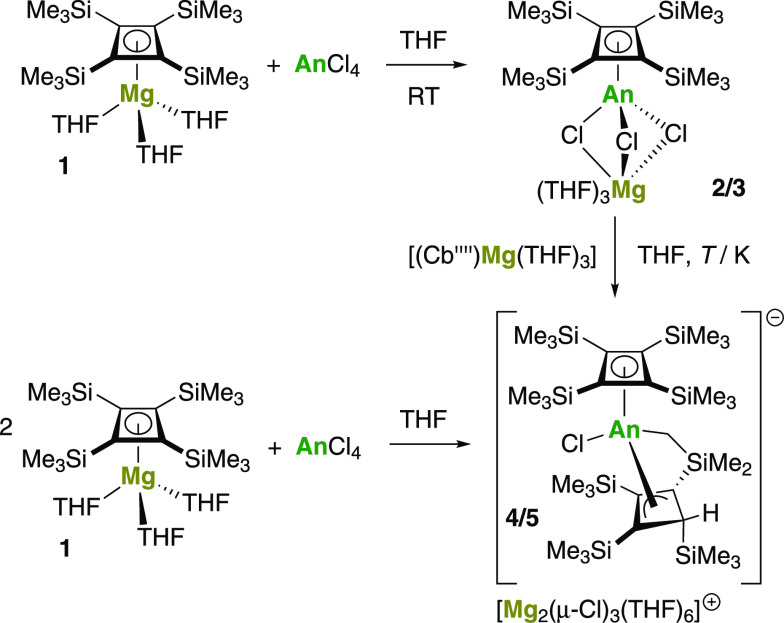
Synthesis of **2** (An = Th), **3** (An = U), [Mg_2_Cl_3_(THF)_6_][**4**] (An = Th, 55 °C), and [Mg_2_Cl_3_(THF)_6_][**5**] (An = U, room temperature)

Adding one equivalent of **1** to ThCl_4_ in THF-D_8_ at −35 °C followed by slow warming to room temperature produced a yellow-orange solution. After stirring for 15 min, complete consumption of **1** was confirmed by ^1^H nuclear magnetic resonance (NMR) spectroscopy, with a resonance observed at 0.23 ppm, suggesting the formation of an {(η^4^-Cb'''')Th} unit (Figure S1). Work-up of the reaction followed by recrystallization produced the half-sandwich complex [(η^4^-Cb'''')ThCl(μ-Cl)_3_Mg(THF)_3_] (**2**) in an isolated yield of 65% (Scheme 1). With UCl_4_ as the starting material, the same procedure was used to synthesize the analogous uranium(IV) compound [(η^4^-Cb'''')UCl(μ-Cl)_3_Mg(THF)_3_] (**3**) in 48% isolated yield.

**Figure 1:**
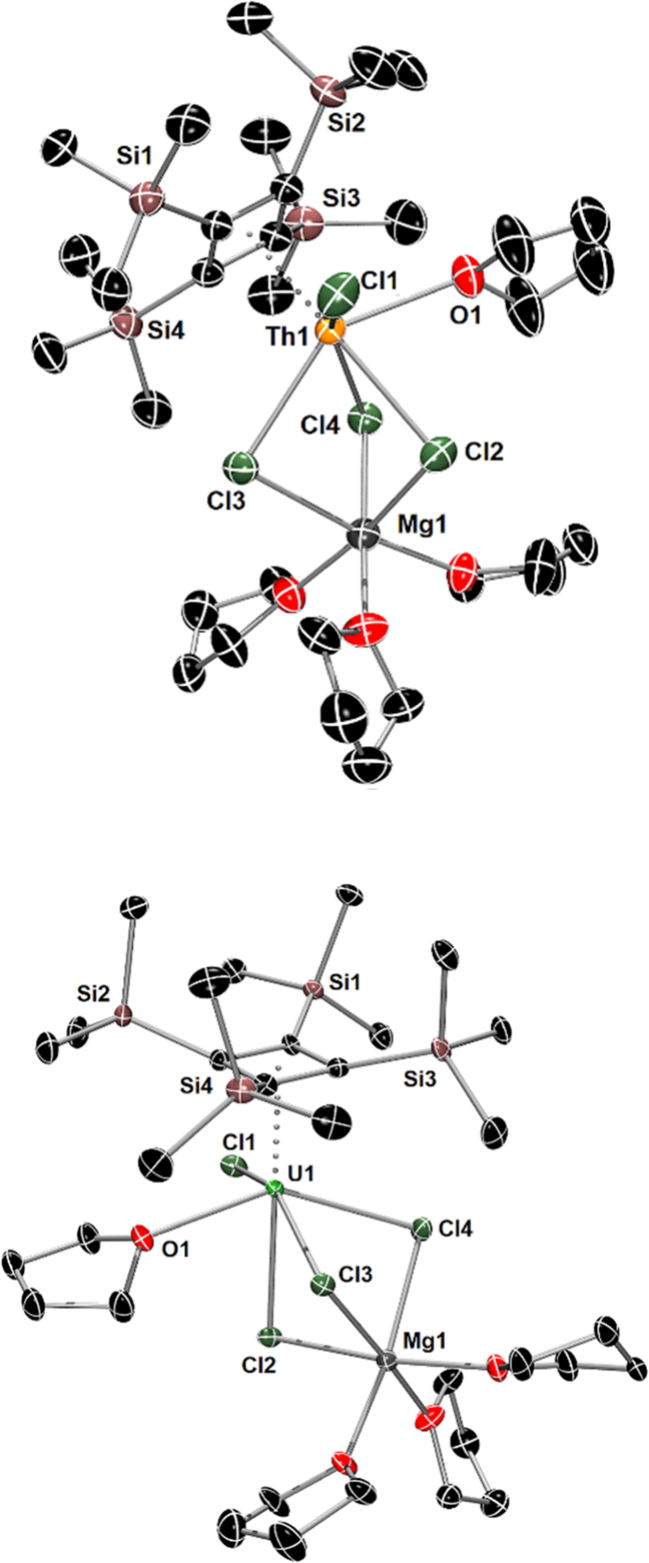
Thermal ellipsoid plots (50% probability) of the molecular structures of **2** (upper) and **3** (lower). Hydrogen atoms are omitted for clarity.

The molecular structures of **2** and **3** were determined by X-ray crystallography and found to be very similar, with each consisting of an η^4^-cyclobutadienyl ligand coordinated to the actinide center (Figure 1 and Table S1). The coordination environments of thorium and uranium additionally contain a tetrahydrofuran (THF) ligand, a terminal chloride ligand, and three μ-chloride ligands bridging to magnesium. The An–Cb'''' distances to the centroid of the ligand in **2** and **3** are 2.396(15) and 2.330(19) Å, respectively, with average Th–C and U–C distances of 2.614 and 2.552 Å, respectively. The planarity of the cyclobutadienyl ligands in both compounds is reflected in the C1–C2–C3–C4 torsional angles of 0.2(2)° for **2** and 0.1(3)° for **3**. A distinct bending of the trimethylsilyl groups out of the C_4_ plane, away from the actinide, is reflected in C–C–C–Si torsional angles in the range 150.8(3)–161.9(2)° for **2** and 150.4(3)–161.8(3)° for **3**. Overall, the geometric parameters associated with the η^4^-Cb'''' ligand in **2** and **3** are consistent with those found in other thorium(IV) and uranium(IV) cyclobutadienyl complexes. Selected other distances and angles are shown in Table 1.

**Table 1:** Selected Distances (Å) and Angles (°) for **2**–**5**

	**2**	**3**	**4**	**5**
An–(η^4^-Cb'''')	2.396(15)	2.330(19)	2.462(4)	2.378(5)
An–C (η^4^-Cb'''')	2.597(3)–2.630(3)	2.523(4)–2.585(4)	2.636(7)–2.674(10)	2.557(10)–2.635(10)
	av. 2.614	av. 2.552	av. 2.673	av. 2.597
An–(η^3^-Cb'''(H))			2.551(4)	2.492(6)
An–C (η^3^-Cb'''(H))			2.700(6)–2.796(7)	2.631(11)–2.758(10)
			av. 2.734	av. 2.677
An–C1			2.548(5)	2.499(11)
An···H2			2.441	2.344
An–Cl1	2.7083(7)	2.6557(9)	2.7213(15)	2.654(3)
An–(μ-Cl)	2.8191(8)–2.9090(8)	2.7890(9)–2.8359(9)		
An–O1	2.532(3)	2.514(3)		
Cb''''–An–Cb'''(H)			148.4(11)	150.8(19)

The solution-phase structures of isolated **2** and **3** are consistent with their solid-state structures. In THF-D_8_, the ^1^H NMR spectrum of **2** shows a resonance at 0.23 ppm due to the SiMe_3_ substituents, and resonances for the THF/THF-D_8_ ligands (approximate 1:3 ratio) were observed around 3.58 and 1.80 ppm (Figure S2). The ^13^C{^1^H} NMR spectrum features resonances at 5.20 ppm for the methyl groups and 141.98 ppm for the cyclobutadienyl carbon atoms (Figure S3). A single resonance in the ^29^Si NMR spectrum was also observed at 21.30 ppm (Figure S4). In the ^1^H NMR spectrum of **3**, the SiMe_3_ groups resonate at −4.37 ppm, and the THF/THF-D_8_ ligands (approximate 2.5:1.5 ratio) occur at 3.61 and 1.76 ppm (Figures S6 and S7). A single resonance in the ^29^Si NMR spectrum was observed at −183.77 ppm (Figure S8), which falls within the chemical shift range expected for silyl-substituted organometallic complexes of uranium(IV).^40^

**Figure 2:**
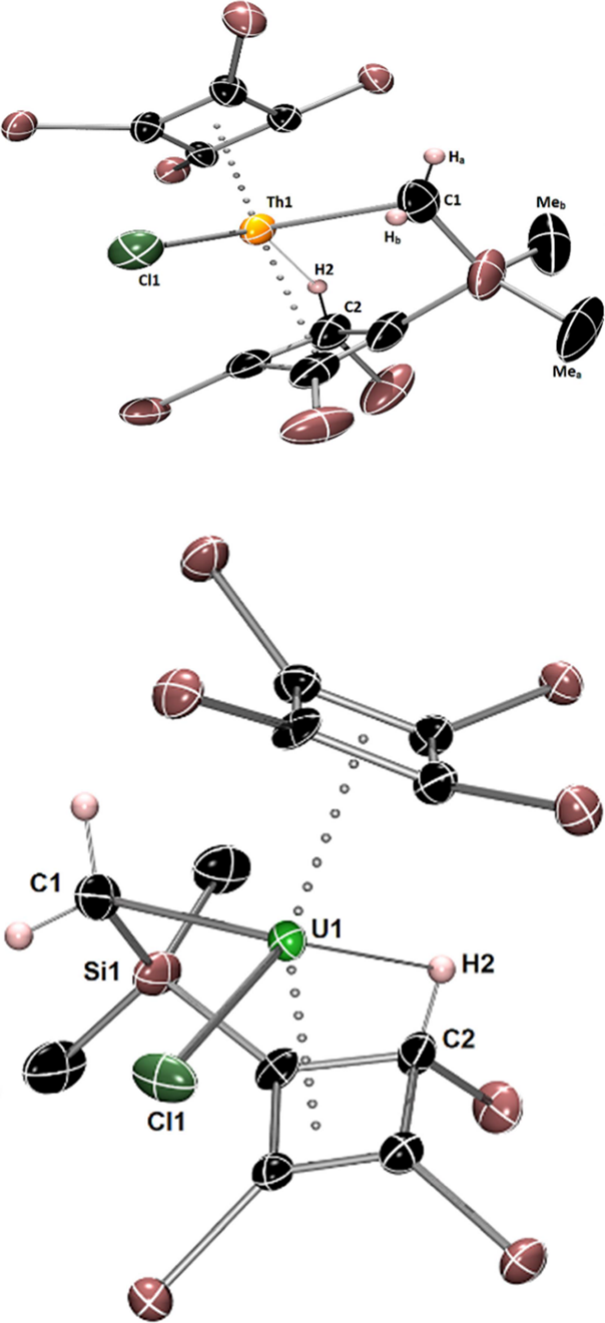
Thermal ellipsoid plots (50% probability) of the molecular structures of **4** (upper) and **5** (lower). Hydrogen atoms, except those originating from the cyclometalated trimethylsilyl group, are omitted for clarity.

The intact transfer of the Cb'''' ligand to give **2** and **3** prompted us to undertake the 2:1 stoichiometric reactions of **1** with ThCl_4_ and UCl_4_, aiming to synthesize the actinocenes [An(η^4^-Cb'''')_2_]. After stirring for several hours at room temperature, the ^1^H NMR spectrum of the reaction mixture containing **1** and ThCl_4_ in THF-D_8_ consists of equimolar amounts of **1** and **2** (Figures S11). However, significant changes occurred after heating an aliquot of the mixture to 55 °C overnight, with the observation of six signals in a ratio of 3:9:9:36:3:9 in the silyl region of the ^1^H NMR spectrum, i.e., 0.13–0.26 ppm, indicating one intact η^4^-Cb'''' ligand and the other ligand having undergone activation at a trimethylsilyl group (Figure S12). Filtration of the reaction mixture and addition of *n*-heptane followed by slow evaporation of the solvent produced crystals that were subsequently identified by X-ray crystallography to consist of ion-separated [Mg_2_Cl_3_(THF)_6_][(η^4^-Cb'''')Th(η^3^-C_4_H(SiMe_3_)_3_-κ-(CH_2_SiMe_2_)(Cl)] ([Mg_2_Cl_3_(THF)_6_][**4**], Figure 2 and Table S1), in which the anion **4** shows cyclometalation of a Cb'''' ligand via a methyl group and protonation of the same Cb'''' ligand to its allylic form. The analogous uranium(IV) compound [Mg_2_Cl_3_(THF)_6_][(η^4^-Cb'''')U(η^3^-C_4_H(SiMe_3_)_3_-κ-(CH_2_SiMe_2_)(Cl)]^−^ ([Mg_2_Cl_3_(THF)_6_][**5**]) was synthesized at room temperature with stirring for only 3 h. The isolated yields of the thorium and uranium sandwich compounds were 66 and 53%, respectively.

Complexes **4** and **5** are essentially isostructural, featuring an η^4^-Cb'''' ligand bound to the thorium and uranium center, respectively (Figure 2). The second cyclobutadienyl ligand in both complexes has been deprotonated at a methyl group, resulting in a cyclometalated silylmethyl ligand σ-bound to thorium and uranium in **5** and **6**, respectively. The hydrogen has seemingly been transferred to the same four-membered carbocyclic ring to give an allylic η^3^-Cb(H)''' ligand, with the methine hydrogen oriented toward the actinide center. A chloride ligand completes the coordination environment of the metal in each complex. Compared to **2**, the Th–Cb'''' centroid distance in **4** is slightly longer at 2.462(4) Å, as is the U–Cb'''' centroid distance of 2.378(5) Å in **5** when compared to **3** (Table 1). The Th–Cb(H)''' and U–Cb(H)''' centroid distances to the allylic ligands are significantly longer at 2.551(4) and 2.492(6) Å in **4** and **5**, respectively. Appreciable bending of the sandwich structures is reflected in the Cb''''–An–Cb(H)''' angles of 148.4(11)° and 150.8(19)° in **4** and **5**, respectively. The orientation of the methine hydrogen atom on the Cb(H)'''' ligands (added to the structures using the riding model) toward the metal produces Th···H2 and U···H2 separations of 2.441 and 2.344 Å, respectively. The cyclometalated Th–C1 and U–C1 distances are 2.548(5) and 2.499(11) Å in **4** and **5**, respectively.

The ^1^H and ^29^Si NMR spectra of isolated [Mg_2_Cl_3_(THF)_6_][**4**] in THF-D_8_ at +30 °C are consistent with the solid-state structure. In addition to the single resonance due to the trimethylsilyl groups on the Cb'''' occurring at δ(^1^H) = 0.21 ppm, signals due to the trimethylsilyl groups of the Cb(H)''' ligand were also observed at 0.18, 0.19, and 0.35 ppm, with the diastereotopic methyl groups on the cyclometalated carbon atom occurring at 0.13 and 0.26 ppm, respectively (Figures S13 and S14). Two mutually coupled doublets, each integrating to one proton, occur at −2.57 and 1.54 ppm with ^2^*J*_HH_ = 11.7 Hz and correspond to the diastereotopic protons on the cyclometalated carbon. This assignment was further confirmed with a heteronuclear single quantum coherence (gHSQC) experiment, which showed a correlation at δ(^13^C) = 25.8 ppm for the cyclometalated carbon (Figures S16 and S17). Five resonances were also observed in the ^29^Si{^1^H} NMR spectrum in the range −6.35 to −25.13 ppm (Figure S18 and S19). The ^13^C{^1^H} NMR spectrum of [Mg_2_Cl_3_(THF)_6_][**4**] at +30 °C shows six resonances in the region 3.24–5.73 ppm for the methyl carbon atoms and a resonance at 25.8 ppm for the cyclometalated carbon (Figure S15). The cyclobutadienyl carbons on the intact Cb'''' ligand occur at 139.13 ppm. However, the ^13^C{^1^H} NMR spectrum did not show any resonances that could be assigned to the ring carbon atoms of the cyclometalated Cb(H)''' ligand even when applying long acquisition and relaxation times to concentrated solutions (i.e., overnight, 2 s, >80 mg mL^–1^). This observation prompted us to undertake experiments at lower temperatures to determine whether a fluxional process was occurring (Figures S20 and S21).

In the ^1^H NMR spectrum of [Mg_2_Cl_3_(THF)_6_][**4**] at +30 °C, the broad singlet at 0.65 ppm with an FWHM of 7.71 Hz (FWHM = full width at half-maximum) moves to 0.62 ppm upon cooling to −30 °C and sharpens to an FWHM of 2.14 Hz (Figure S22). No other significant changes were observed. Multinuclear NMR characterization of the compound at −30 °C was then undertaken and found to be fully consistent with the solid-state structure, with three new resonances observed at δ(^13^C) = 175.67, 120.14, and 121.95 ppm for the allylic carbons of the Cb(H)''' ligand (Figure S23). A new resonance was also observed at δ(^13^C) = 55.21 ppm and assigned to the non-allylic carbon atom in the ring of the Cb(H)''' ligand using a gHSQC experiment, with ^1^*J*_CH_ = 110.87 Hz (Figures S24 and S25). These observations indicate that the methine proton is fluxional in solution at room temperature. This was further confirmed by ^29^Si–^1^H heteronuclear multiple-bond coherence (gHMBC) experiments at +30 and −30 °C, which show no correlation between the proton and any trimethylsilyl groups at the higher temperature but two correlations at the lower temperature with trimethylsilyl groups occurring at δ(^29^Si) = −15.69 and −6.17 ppm (Figures S26–S28). A ^29^Si–^1^H gHMBC experiment allowed the silicon bound to the cyclometalated carbon to be assigned to δ(^29^Si) = −25.19 ppm owing to its correlation with the diastereotopic methyl groups and protons in **4** (Figures S27 and S28). Using one-dimensional (1D) nuclear Overhauser effect spectroscopy (NOESY, Figures S29–S34), these trimethylsilyl groups were assigned as the *meso* substituent on the η^3^-allyl ligand and the geminal substituent relative to the methine proton, respectively. 1D-NOESY also allowed the diastereotopic methyl protons to be assigned to resonances at δ(^1^H) = 0.25 and 0.10 ppm, respectively, and the diastereotopic methylene protons at 1.57 and −2.67 ppm, respectively.

The NMR spectra of the analytically pure [Mg_2_Cl_3_(THF)_6_][**5**] in THF-D_8_ at +30 °C are more complicated. ^1^H NMR resonances assignable to the cyclometalated complex **5** were observed at −1.65 ppm for the η^4^-Cb'''' ligand, at 10.73, 6.56, and −8.09 ppm for the trimethylsilyl groups on the η^4^-Cb(H)''' ligand, at 15.76 and −23.81 ppm for the diastereotopic methyl groups, and at −77.35, −108.62, and −140.00 ppm for the diastereotopic methylene protons and the methine proton (Figures S36–S39). In addition, evidence for a second, minor (approximately 7%) species was also found in the ^1^H NMR spectrum. At +30 °C, this minor species gives rise to three singlets at 15.17, −0.69, and −9.63 ppm, with relative integrations of 3:9:1 (Figure S39). Consistent with these observations, the ^29^Si{^1^H} NMR spectrum at +30 °C shows resonances due to the major species **5** at 127.22, 59.90, −103.98, −176.81, and −199.45 ppm, as expected based on the solid-state structure (Figure S40). Two additional ^29^Si NMR resonances for the minor species were observed at 81.18 and −152.79 ppm. Upon cooling to −50 °C and below, a total of six resonances unassignable to **5** were observed in the ^1^H NMR spectrum (Figures S41–S44). At −77 °C, these resonances occur at 53.43, 25.11, 24.57, −8.99, −14.31, and −60.44 ppm, with relative integrations of 6:9:18:18:9:12 (Figures S45 and S46). The ^29^Si{^1^H} NMR spectrum at −77 °C shows three new minor peaks at 340.75, 143.80, and −236.10 ppm alongside the five major peaks for **5** (Figure S47).

**Scheme 2:**
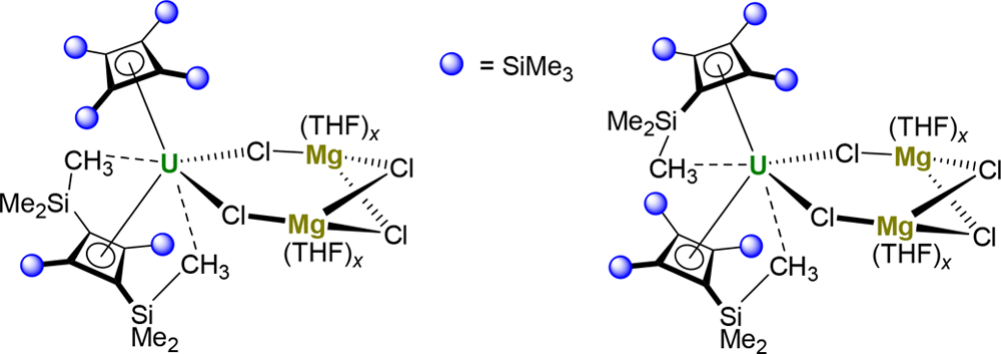
Possible Structures of the Minor Species Present in Solution with [Mg_2_Cl_3_(THF)_6_][**5**]

While the structure of the minor species in solution cannot be proposed with certainty, the relative integrals in the ^1^H NMR spectrum are notably present as multiples of 3, with no associated methylene or methine protons. We can therefore propose that this minor species contains two intact η^4^-Cb'''' ligands. The low symmetry indicated by the ^1^H NMR spectrum of this minor uranium-containing species can be accounted for by an ion-separated complex such as [(η^4^-Cb'''')_2_UCl]^−^ or an ion-contact heterobimetallic complex such as [(η^4^-Cb'''')_2_U(μ-Cl)_2_{Mg(THF)_3_}_2_] (Scheme 2), with U···CH_3_ contacts reminiscent of those observed in the solid-state structures of the uranium(III) half-sandwich compound [(η^5^-Cp*)U{N(SiMe_3_)_2_}_2_] (Cp* = C_5_Me_5_).^41^ Indeed, our computational study of the reaction mechanism for the formation of **4** and **5** found such interactions to be relevant (see below). The ^29^Si{^1^H} NMR spectrum of the minor species is broadly consistent with the proposed structures in Scheme 2, showing three resonances at 340.75, 143.80, and −236.10 ppm.

A further noteworthy feature of the ^1^H NMR spectrum of [Mg_2_Cl_3_(THF)_6_][**5**] at +30 °C is the singlet at δ(^1^H) = −8.09 ppm with FWHM = 44.2 Hz, which was assigned to a trimethylsilyl group. Upon cooling, this signal broadens significantly (FWHM ≈ 1350 Hz) and is essentially indistinguishable from the baseline at −30 °C (Figure S48). An insight into this phenomenon was obtained from variable-temperature ^25^Mg{^1^H} NMR spectroscopy. At 30 °C, the ^25^Mg{^1^H} NMR spectrum in THF-D_8_ consists of a single resonance at 2.95 ppm with FWHM = 97.0 Hz, whereas at −30 and −77 °C, this signal is unobservable. This can be interpreted in terms of association of the [Mg_2_Cl_3_(THF)_6_]^+^ cation with the paramagnetic uranium complex **5** at low temperatures and dissociation into separated ions at higher temperatures, with the dynamic process also being associated with the broadening of the ^1^H NMR resonance at δ(^1^H) = −8.09 ppm.

### Computational Studies of Ligand C–H Activation

**Figure 3:**
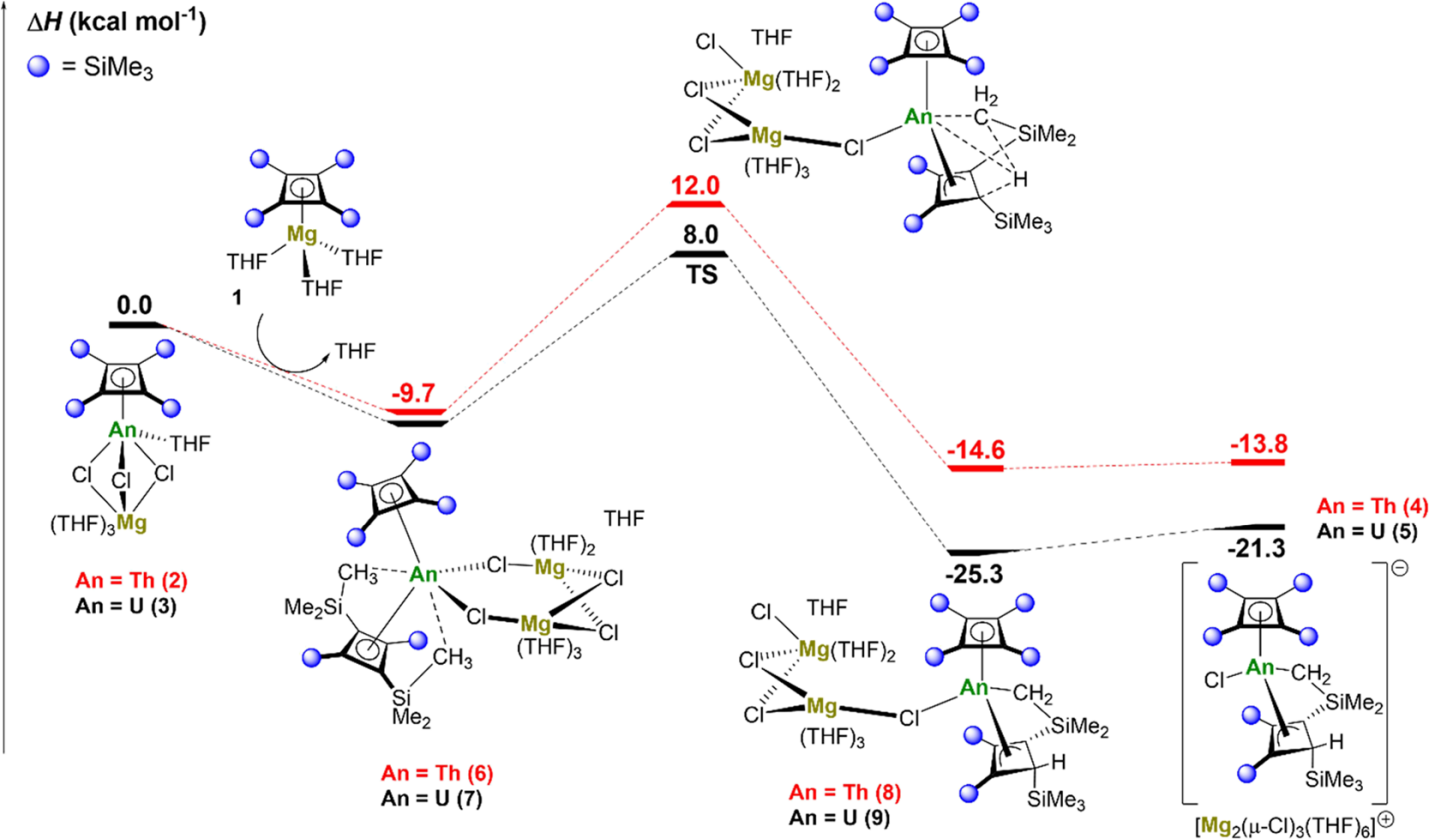
Computed enthalpy profile at room temperature for the formation of **4** and **5** from **2** and **3**, respectively. Energies are stated in kcal mol^–1^.

Alluded to in the introduction, the mechanism through which a trimethylsilyl substituent on the Cb'''' ligand becomes activated and the proton is transferred to the four-membered ring has not previously been elucidated. To gain an understanding of this process, density functional theory (DFT) was used to compute reaction enthalpy profiles for the formation of **4** and **5** at the B3PW91 level of theory (Figure 3).

The reaction was investigated starting from complexes **2** and **3**, which begins with each half-sandwich complex reacting with a molecule of **1**. This reaction is computed to be favorable by 9.7 kcal mol^–1^ for thorium and 10.2 kcal mol^–1^ for uranium. The two intermediates, labeled **6** and **7** in Figure 3, have bent metallocene-like structures with two μ-chloride ligands bridging to magnesium. In agreement with the structure proposed from the NMR experiments (Scheme 2), two close contacts between methyl groups from the same Cb'''' ligand and the actinide center are also computed. From these intermediates, it was possible to locate a C–H activation transition state (TS). The barrier is 21.7 kcal mol^–1^ for thorium and 18.2 kcal mol^–1^ in the case of uranium. Although the difference between these two barriers is within the precision of the method, there is a slight kinetic preference for C–H activation at uranium compared to thorium. At the TS, the former actinide–methyl interactions are broken, and the hydrogen begins transferring as a proton to a Cb'''' ring carbon. Following the intrinsic reaction coordinate from the TS yields intermediates **8** and **9**. For uranium, the molecular orbital involved is the highest occupied molecular orbital, which clearly shows a 5f orbital at uranium overlapping with the cyclometalated carbon (Figures S50 and S51). The transformation of uranium complex **7** into **9** is strongly exothermic by 15.1 kcal mol^–1^, indicating that this reaction is almost thermodynamically irreversible. This is in line with the experimental observation that the reaction is relatively fast for uranium (i.e., 3 h at room temperature). In the case of thorium, formation of complex **8** from **6** is only exothermic by 4.9 kcal mol^–1^. Therefore, within the precision of the method, this step can be considered as an equilibrium between **6** and **8**. This finding is also in line with the experimental conditions, where an overnight reaction at 55 °C was needed. In the last step, the dimagnesium complex [Mg_2_Cl_3_(THF)_6_]^+^ is released from the actinide coordination sphere. This final step is slightly endothermic in both cases, making the overall formation of the thorium complex **4** from **2** exothermic by 13.8 kcal mol^–1^ and the formation of the uranium complex **5** from **3** exothermic by 21.3 kcal mol^–1^.

## Conclusions

In summary, we have shown that intact transfer of one equivalent of the tetrakis(trimethylsilyl)cyclobutadienyl ligand Cb'''' to thorium(IV) and uranium(IV) can be achieved using the magnesium reagent [(η^4^-Cb'''')Mg(THF)_3_] with ThCl_4_ and UCl_4_ as the actinide starting materials, giving the half-sandwich complexes **2** and **3**. Transfer of a second equivalent of the ligand is accompanied by cyclometalation of a trimethylsilyl substituent with transfer of a proton to the same Cb'''' ring, resulting in the formation of the heteroleptic cyclobutadienyl-allyl sandwich complexes **4** and **5**. Consistent with experimental observations, a DFT study of the reaction mechanism revealed the reactivity to be energetically more favorable for uranium. Complicated solution-phase dynamics of the mixed-ligand uranium complex **5** was revealed by multinuclear NMR spectroscopy, with the potential involvement of a bis(cyclobutadienyl)uranium sandwich complex as a minor species alongside **5**. Also consistent with experimental structural proposals, the mechanistic DFT analysis identified such a sandwich complex as an intermediate species. Calculation of a TS revealed that cyclometalation of the trimethylsilyl group involves C–H activation via the actinide center, with intramolecular transfer of a proton to a Cb'''' ligand ultimately generating the observed complexes **4** and **5**. The DFT analysis gives an insight, at least in principle, into how a complex of the type [U(η^4^-C_4_R_4_)_2_] might be stabilized, such as through the use of cyclobutadienyl substituents without C–H bonds in α-positions relative to silicon.

## Data Availability

Additional research data supporting this publication are available at https://doi.org/10.25377/sussex.21641795.v1.

## Supporting Information Available

Synthesis, spectroscopic characterization, crystallography details, and computational details (PDF)
