# Supplementary material for: Thorium- and Uranium-Mediated C–H Activation of a Silyl-Substituted Cyclobutadienyl Ligand
Source: Inorg Chem. 2022 Dec 9;61(50):20629–35. doi: 10.1021/acs.inorgchem.2c03534 (PMC9768750; doi:10.1021/acs.inorgchem.2c03534)
Supplement: Supplementary file 1 — ic2c03534_si_001.pdf [file ic2c03534_si_001.pdf]

Supporting Information

Thorium- and Uranium-Mediated C–H Activation of a Silyl-Substituted Cyclobutadienyl Ligand

Nikolaos Tsoureas,†[a] Thayalan Rajeshkumar,[b] Oliver P. E. Townrow,[c]
Laurent Maron,[b,*] and Richard A. Layfield[a,*]

[a] Department of Chemistry, School of Life Sciences, University of Sussex, Brighton, BN1 9QJ, U.K.

[b] Laboratoire de Physique et Chimie des Nano-objets, Institut National des Sciences Appliquées, 31077 Cedex 4 Toulouse, France

[c] Department of Chemistry, University of Oxford, Chemistry Research Laboratory, 12 Mansfield Road, Oxford, OX1 3TA, U.K.

General Considerations

All manipulations were carried out using glovebox or standard Schlenk techniques. All glassware, cannulas and glass microfibre filters were dried at 160°C overnight prior to use. THF and heptane were dried over molten potassium, distilled under a nitrogen atmosphere and stored in ampoules over activated 4 Å molecular sieves under Ar (BOC PureShield). THF was further dried by stirring overnight at RT over sodium-potassium alloy until a blue hue was observed and vacuum transferred over activated 4 Å molecular sieves. *n*-Heptane was further dried over activated 4 Å molecular sieves overnight followed by transferring to an ampoule containing a fresh potassium mirror. Deuterated THF was degassed by three freeze-thaw cycles, dried by refluxing over molten potassium for three days, vacuum distilled and stored in Young's ampoules over 4 Å molecular sieves under N_2 in a glovebox. Published procedures were used to synthesize UCl_4[1] and [(η^4-Cb'''')Mg(THF)$_3$].[2] $ThCl_4$ was also prepared by a literature method[3] and the product was subjected to sublimation under high vacuum (10^{-5}-10^{-7} mbar) at *ca* 50°C to remove the hexachlorobenzene by-product. ^{1}H, ^{13}C{^{1}H} and ^{29}Si{^{1}H}-NMR spectra, correlation experiments and 1D-NOESY spectra were recorded either on a Varian VNMR S400 or a Bruker AV500 spectrometer operating at 400 MHz and 500 MHz (^{1}H frequency), respectively, at 30°C unless otherwise stated. ^{25}Mg{^{1}H} NMR spectra were acquired on a Bruker AV500 spectrometer. The ^{1}H spectra were referenced internally to the l proton signals of the solvent or the signals of the solvent (^{13}C{^{1}H}). ^{29}Si{^{1}H} NMR spectra were referenced externally relative to $SiMe_4$ and ^{25}Mg{^{1}H} NMR spectra were referenced externally relative to 11 M $MgCl_2$ in D_2O. NMR spectra were recorded in Young's NMR tubes. IR spectra were recorded inside an argon glovebox using a Bruker Alpha FT-IR spectrometer in ATR mode. Elemental analyses were performed by Microanalytisches Labor Pascher, Germany.

Safety Consideration

Thorium (primary isotope ^{232}Th) and depleted uranium (primary isotope ^{238}U) are weak α-emitters (4.197 MeV) with half-lives of 14.05 billion and 4.47 billion years, respectively. Manipulations and reactions should be carried out in monitored fume hoods or in an inert glovebox in a radiation laboratory equipped with α- and β-counting equipment.

Synthesis of [(η^4-Cb'''')ThCl(THF)(μ-Cl)$_3$Mg(THF)$_3$] (2)

$ThCl_4$ (50 mg, 0.13 mmol) and [(η^4-Cb'''')Mg(THF)$_3$] (**1**) (77 mg, 0.13 mmol) were placed in an ampoule with a glass-coated stirrer bar and the two solids cooled to –35°C. THF-D$_8$ (1-1.5 ml), pre-cooled to –35°C was then transferred to the solids. Stiring the mixture produced a pale-yellow suspension, which gradually turns bright orange solution upon warming to room temperature. Stirring was continued for 15 minutes, after which time complete consumption of **1** was revealed by ^{1}H NMR spectroscopy (Figure S1). The reaction mixture was filtered through a glass microfibre filter into a 20 mL scintillation vial inside a glove-box and was filtered again with THF (5 ml) to removing any remaining precipitate. An equal volume of n-heptane was added to this bright orange solution and the mixture quickly filtered. The vial was capped with a rubber septum equipped

with a venting needle and left in the glovebox at room temperature to slowly evaporate. After two days, large orange crystals of **2** were formed, which were then isolated by removing the supernatant with a pipette, washed with n-heptane, dried in a stream of argon in the glovebox, followed by drying in vacuo for 15 minutes. If an oil formed during crystallisation or the solution became cloudy, dropwise addition of THF until a bright orange solution was obtained, followed by repeating the slow evaporation, allowed crystals of **2** to form (89 mg, 65%).

Analytical data for 2

^{1}H NMR (δ/ppm, THF-D_8): 0.23 (s, 36H, $SiMe_3$); integration of peaks due to the coordinated THF-D_8/THF peaks is approximately 3:1. **^{13}C{^{1}H}-NMR** (δ/ppm, THF-D_8): 141.98 (cyclobutadienyl carbon); 5.20 ($SiMe_3$). **^{29}Si{^{1}H}-NMR** (δ/ppm, THF-D_8): –21.30 ($SiMe_3$). **Elemental Analysis:** Calculated for $C_{32}H_{44}Cl_4MgOSi_4Th{\cdot}3C_4D_8O$: C 38.31, H 4.42. Found: C 38.32, H 4.65.

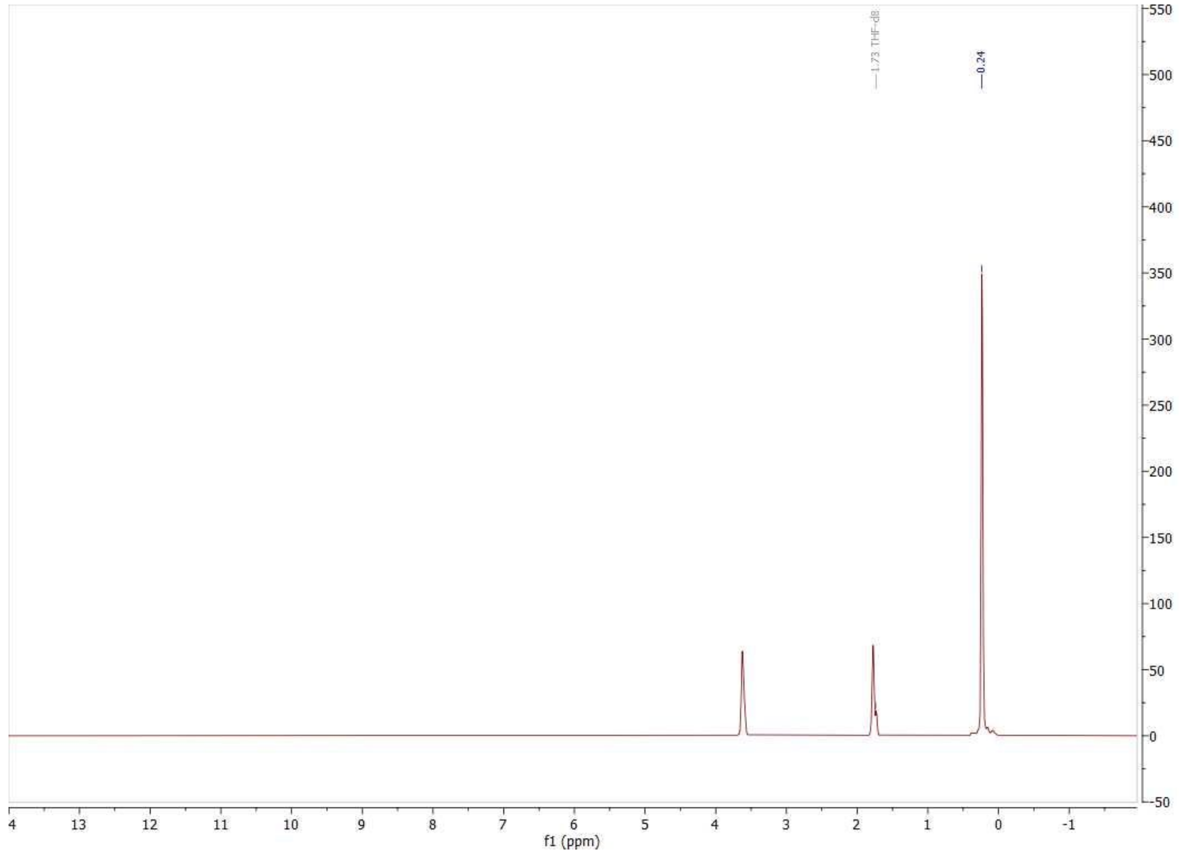

Figure S1. ^{1}H NMR spectrum of the 1:1 reaction between $ThCl_4$ and [(η^4-Cb'''')Mg(THF)$_3$] in THF-D_8.

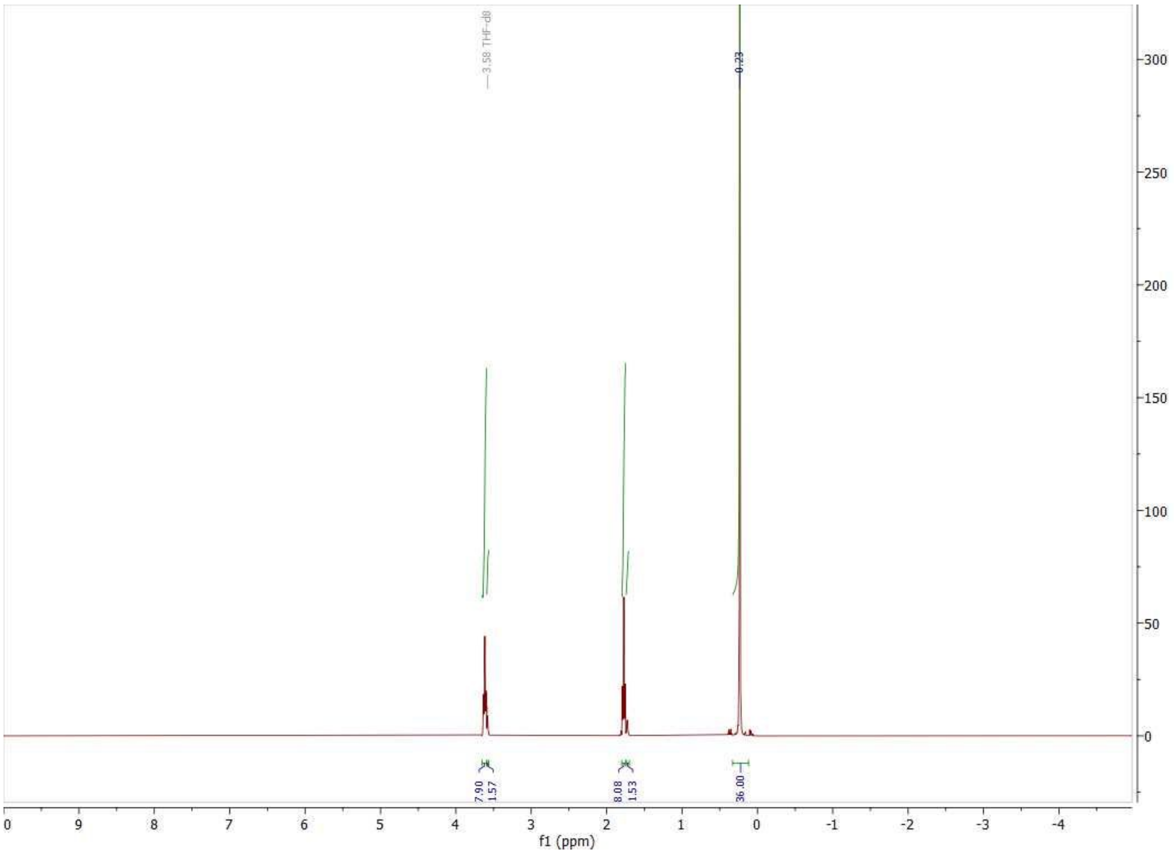

Figure S2. ^{1}H NMR spectrum of **2** in THF-D_8.

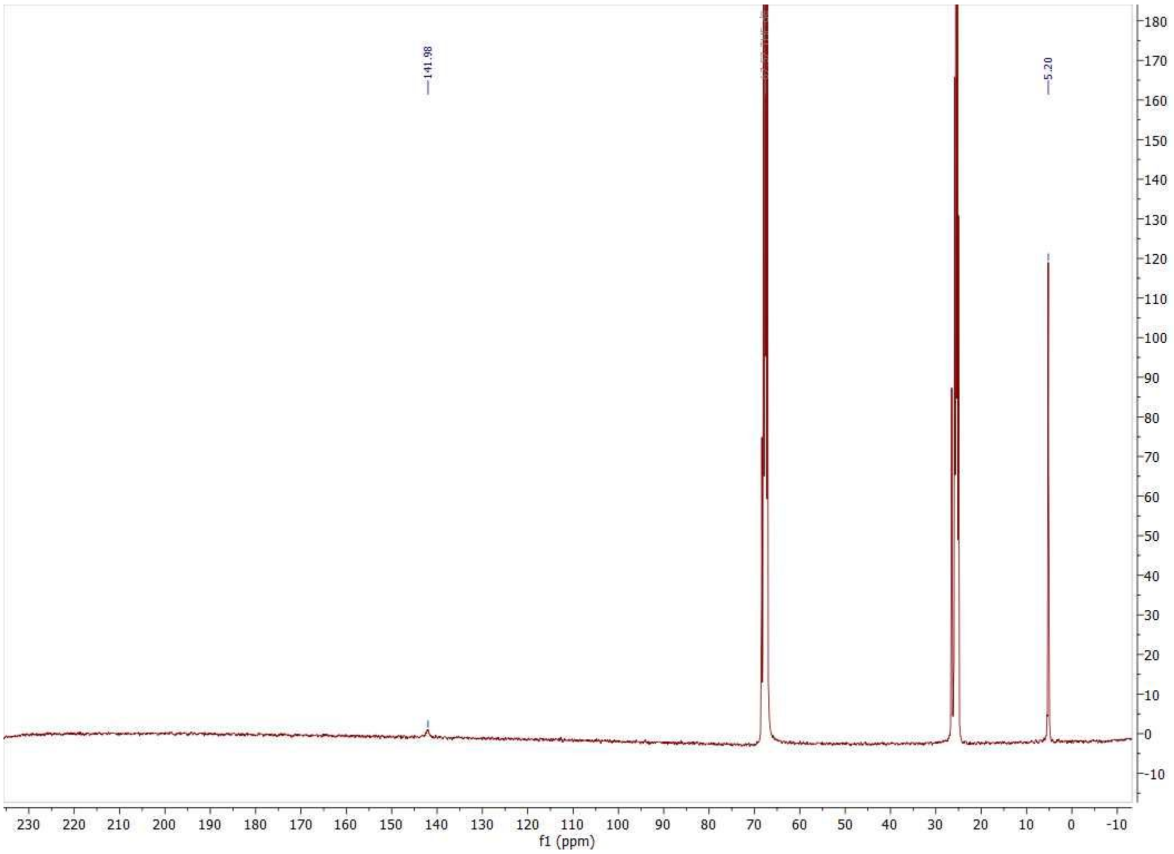

Figure S3. $^{13}C\{^{1}H\}$ NMR spectrum of **2** in THF-D_8.

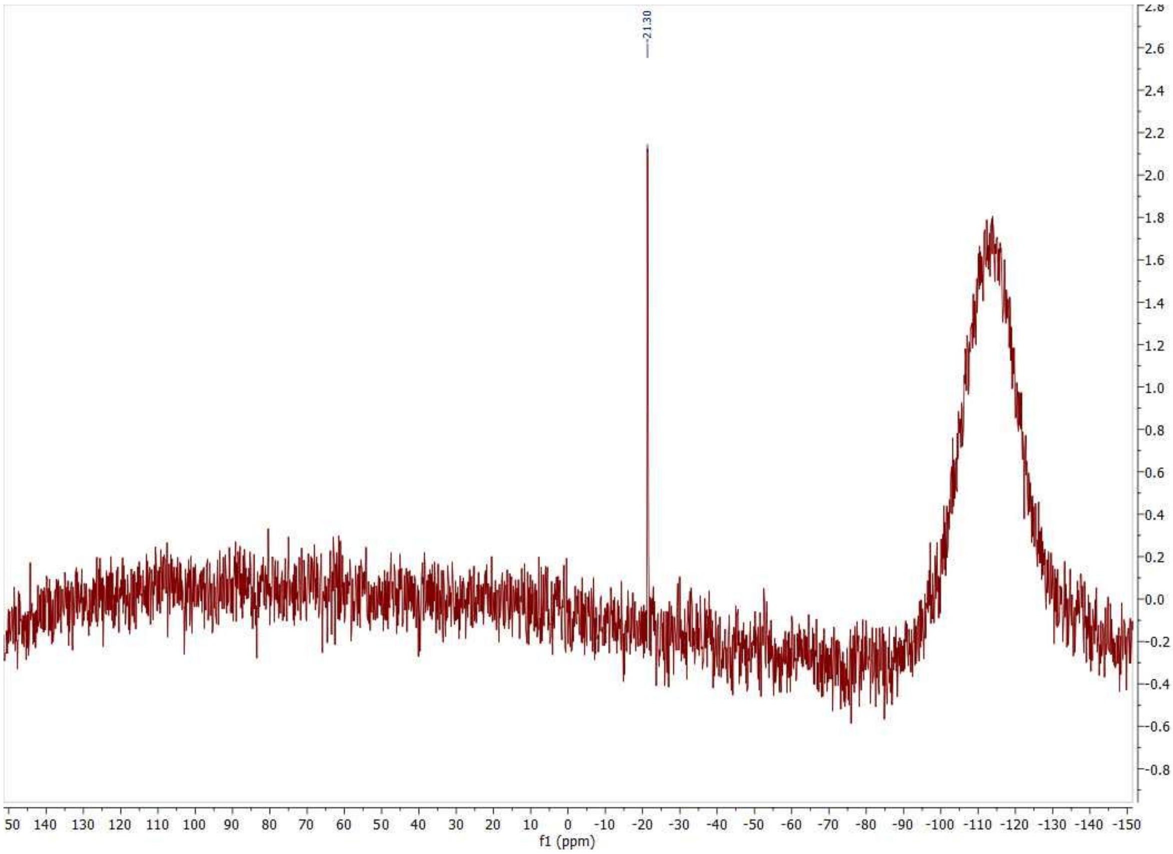

Figure S4. $^{29}Si\{^{1}H\}$ NMR spectrum of **2** in THF-D_8.

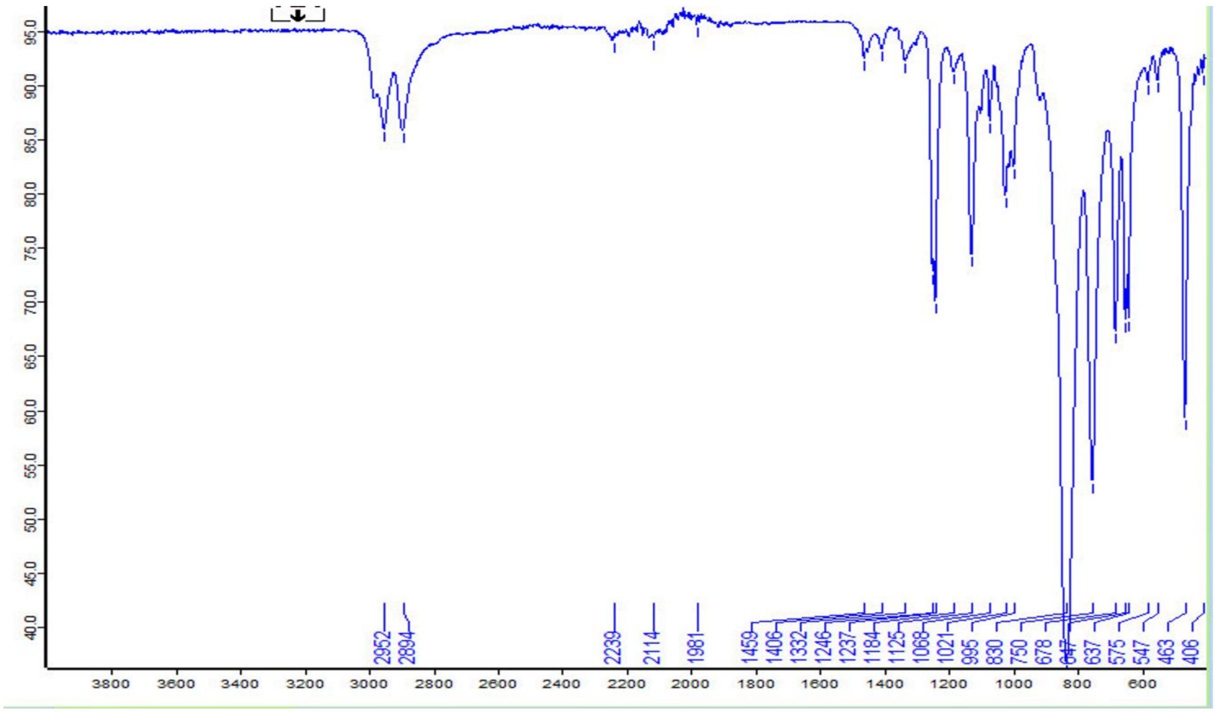

Figure S5. IR spectrum of **2**.

Synthesis of [(η⁴-Cb'''')UCl(THF)(μ-Cl)$_3$Mg(THF)$_3$] (3)

Compound **3** was prepared using a method analogous to that described for **2**, using UCl_4 (81 mg, 0.21 mmol) and [(η⁴-Cb'''')Mg(THF)$_3$] (123 mg, 0.21 mmol) in THF-D_8 (1.5 ml) at –35°C. The reaction developed a dark brown-green colour and was stirred for five minutes at room temperature. Work-up and recrystallization produced **3** as brown crystals (105 mg, 48%).

Analytical data for 3

^{1}H NMR (δ/ppm, THF-D_8): –4.37 (s, 36H, $SiMe_3$); integration of peaks due to the coordinated THF-D_8/THF peaks is approximately 2.5:1.5. **^{29}Si{^{1}H}-NMR** (δ/ppm, THF-D_8): –183.77 (s, $SiMe_3$). **Elemental Analysis:** Calculated for $C_{16}H_{36}Cl_4Si_4U{\cdot}2.5(C_4H_8O){\cdot}1.5(C_4D_8O)$: C 36.76, H 4.39. Found: C 36.59, H 4.89.

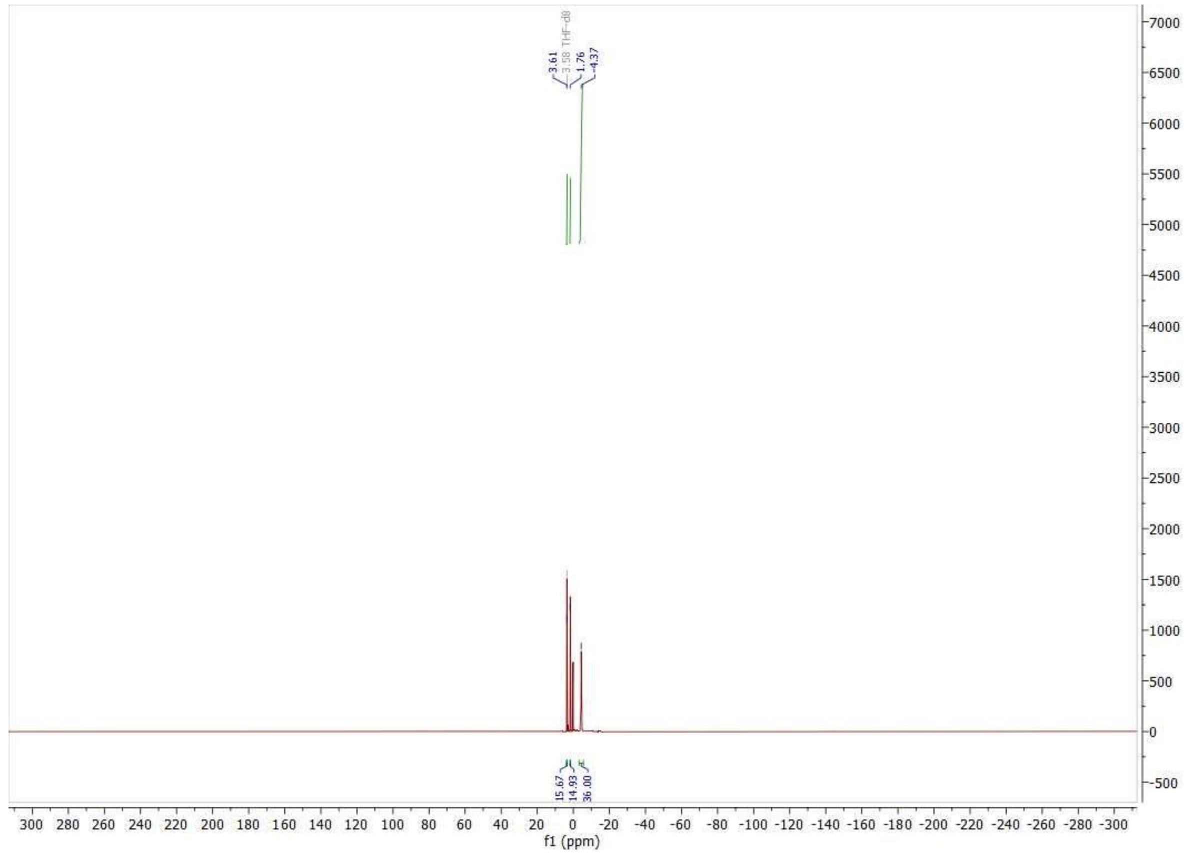

Figure S6. ^{1}H NMR spectrum of **3** in THF-D_8.

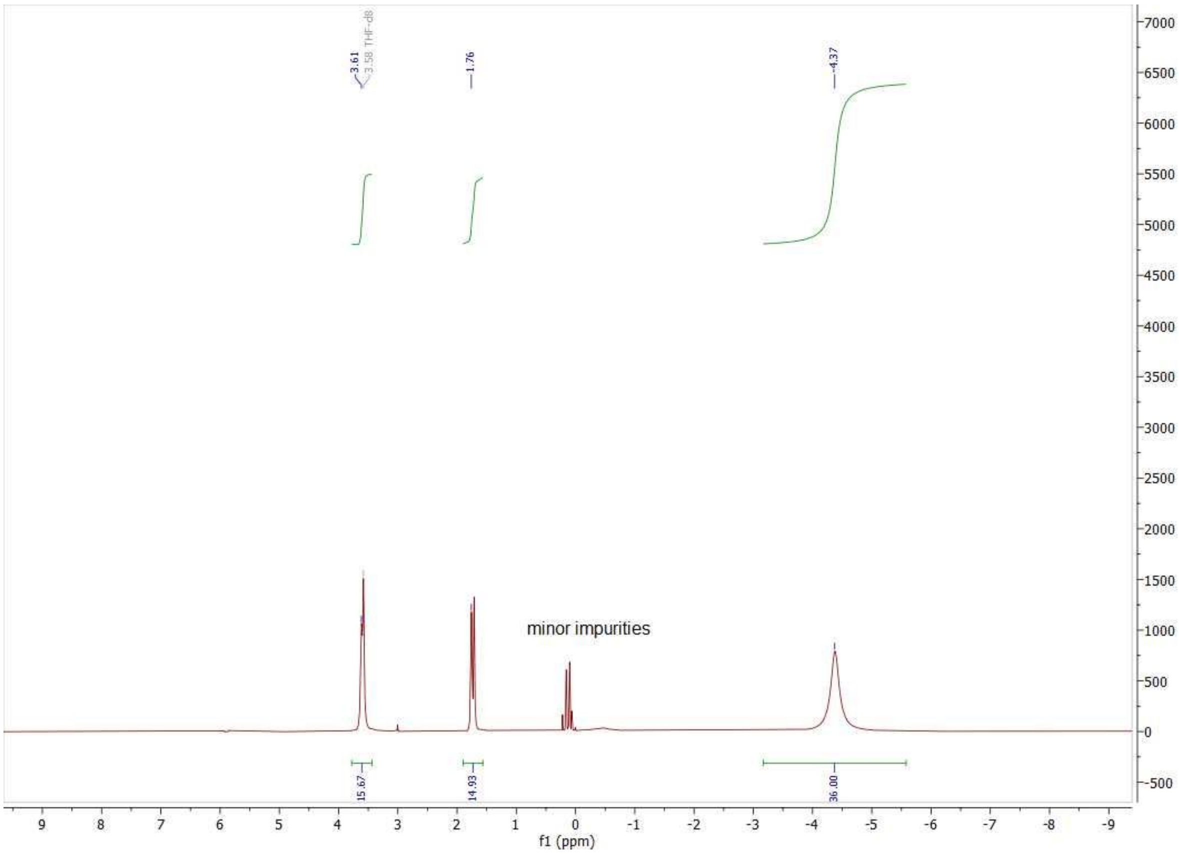

Figure S7. Expanded ^{1}H NMR spectrum of **3** in THF-D_8.

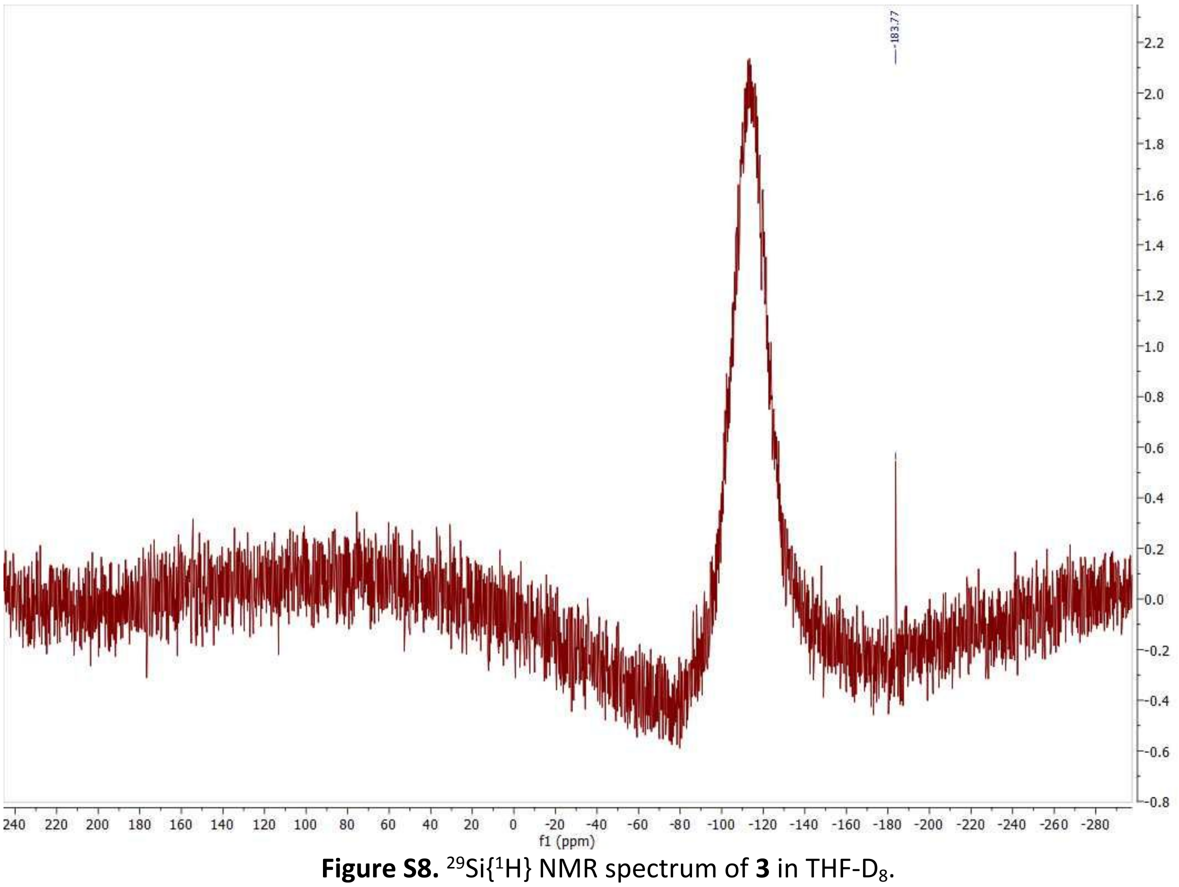

Figure S8. $^{29}Si\{^{1}H\}$ NMR spectrum of **3** in THF-D_8.

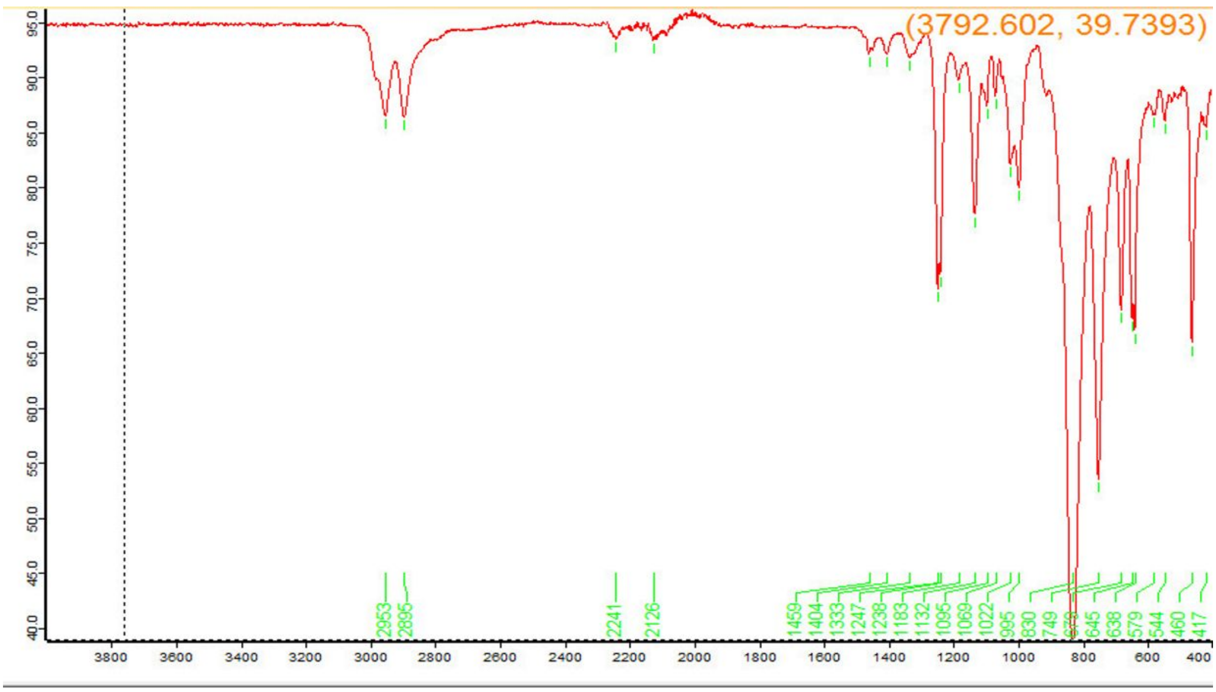

Figure S9. IR spectrum of **3**.

Synthesis of $[Mg_2Cl_3(THF)_6][(\eta^4\text{-Cb''''})Th(\eta^3\text{-}C_4H(SiMe_3)_3\text{-}\kappa\text{-}(CH_2SiMe_2)(Cl)]$ ($[Mg_2Cl_3(THF)_6][4]$)

$ThCl_4$ (100 mg, 0.27 mmol) and $[(\eta^4\text{-Cb''''})Mg(THF)_3]$ (311 mg, 0.54 mmol) were combined as solids and THF-D_8 (2 ml) was added at –35°C. After warming to room temperature and stirring for one hour, an aliquot of the reaction mixture was analysed by ^{1}H NMR spectroscopy, which revealed a 1:1 mixture of **1** and **2** (Figure S11). The reaction mixture was then heated at 55°C overnight, producing a slightly cloudy orange solution. Analysis of the reaction mixture by ^{1}H NMR spectroscopy showed that the major component is the title compound (Figure S12). Filtration of the reaction mixture through micro glass-fibre filter paper followed by extraction into THF (5 ml) produced an orange solution, which was diluted with an equal volume of n-heptane. The vial was capped with a septum equipped with a venting needle and left in the glovebox at room temperature to slowly evaporate for two days. The title compound formed as yellow-orange crystals (270 mg, 66%).

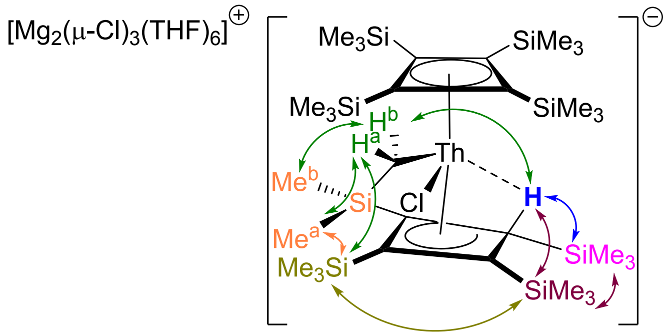

Figure S10. Structure of $[Mg_2Cl_3(THF)_6]$[**4**] with arrows depicting key NOESY correlations.

Analytical data for $[Mg_2Cl_3(THF)_6][4]$

^{1}H NMR (δ/ppm, THF-D_8, +30°C): 1.54 (d, 1H, $^2J_{HH}$ = 11.7 Hz, diastereotopic CH_2); 0.65 (broad s, 1H, FWHM = 7.71 Hz, methine CH); 0.35 (s, 9H, η^3-$SiMe_3$); 0.26 (s, 3H, diastereotopic $SiMe_2$); 0.21 (s, 36H, η^4-$C_4\{SiMe_3\}$); 0.19 (s, 9H, η^3-$SiMe_3$); 0.18 (s, 9H, η^3-Si*Me_3*); 0.13 (s, 3H, diastereotopic $SiMe_2$); –2.57 (d, 1H, $^2J_{HH}$ = 11.7 Hz, diastereotopic CH_2). **$^{13}C\{^1H\}$ NMR** (δ/ppm, THF-D_8, +30°C): 139.13 (η^4-$\underline{C}_4\{SiMe_3\}$); 68.38 (THF); 26.49 (THF); 25.80 (CH_2); 10.41 (diastereotopic $SiMe_2$); 5.73 (η^4-$C_4\{Si\underline{Me_3}\}$); 5.15 (diastereotopic $SiMe_2$); 5.13, 3.24, 3.29 (η^3-$SiMe_3$). **$^{29}Si\{^1H\}$ NMR** (δ/ppm, THF-D_8, +30°C): –6.35, –15.89, –20.17, (η^3-$SiMe_3$); –22.31 (η^4-$C_4\{SiMe_3\}$); –25.13 ($SiMe_2CH_2$).

^{1}H NMR (δ/ppm, THF-D_8, –30°C): 1.57 (d, 1H, $^2J_{HH}$ = 11.9 Hz, diastereotopic H^b); 0.62 (broad s, 1H, FWHM = 2.14 Hz, methine CH); 0.33 (s, 9H, η^3-$SiMe_3$); 0.25 (s, 3H, diastereotopic $SiMe^a$); 0.19 (s, 36H, η^4-$C_4\{SiMe_3\}$); 0.172 and 0.167 (overlapping singlets, 18H, C(H)Si$\underline{Me_3}$ and *meso*-$SiMe_3$, respectively); 0.10 (s, 3H, diastereotopic $SiMe^b$); –2.67 (d, 1H, $^2J_{HH}$ = 11.9 Hz, diastereotopic H^a). **$^{13}C\{^1H\}$ NMR** (δ/ppm, THF-D_8, –30°C): 175.67 (central allylic carbon); 138.37 (η^4-$\underline{C}_4\{SiMe_3\}$); 121.95, 120.14 (terminal allylic carbons); 55.21 (methine carbon, $^1J_{CH}$ = 110.87 Hz); 25.47 (CH_2); 10.57 (diastereotopic $SiMe_2$); 5.60 (η^4-$C_4\{Si\underline{Me_3}\}$); 5.02 (diastereotopic $SiMe_2$); 4.95, 3.14, 3.12 (η^3-$SiMe_3$). **$^{29}Si\{^1H\}$ NMR** (δ/ppm, THF-D_8, +30°C): –22.10 (η^4-$C_4\{SiMe_3\}$); –25.19 ($SiMe_2CH_2$); –20.06 ($SiMe_3$ on terminal allylic carbon); –15.69 ($SiMe_3$ on central allylic carbon); –6.17 (C(H)$SiMe_3$).

Elemental Analysis: Calculated for $C_{56}H_{120}Cl_4Mg_2O_6Si_8Th.0.1C_7H_{16}$: C 44.03. H 7.92; Found: C 44.21, H 7.83.

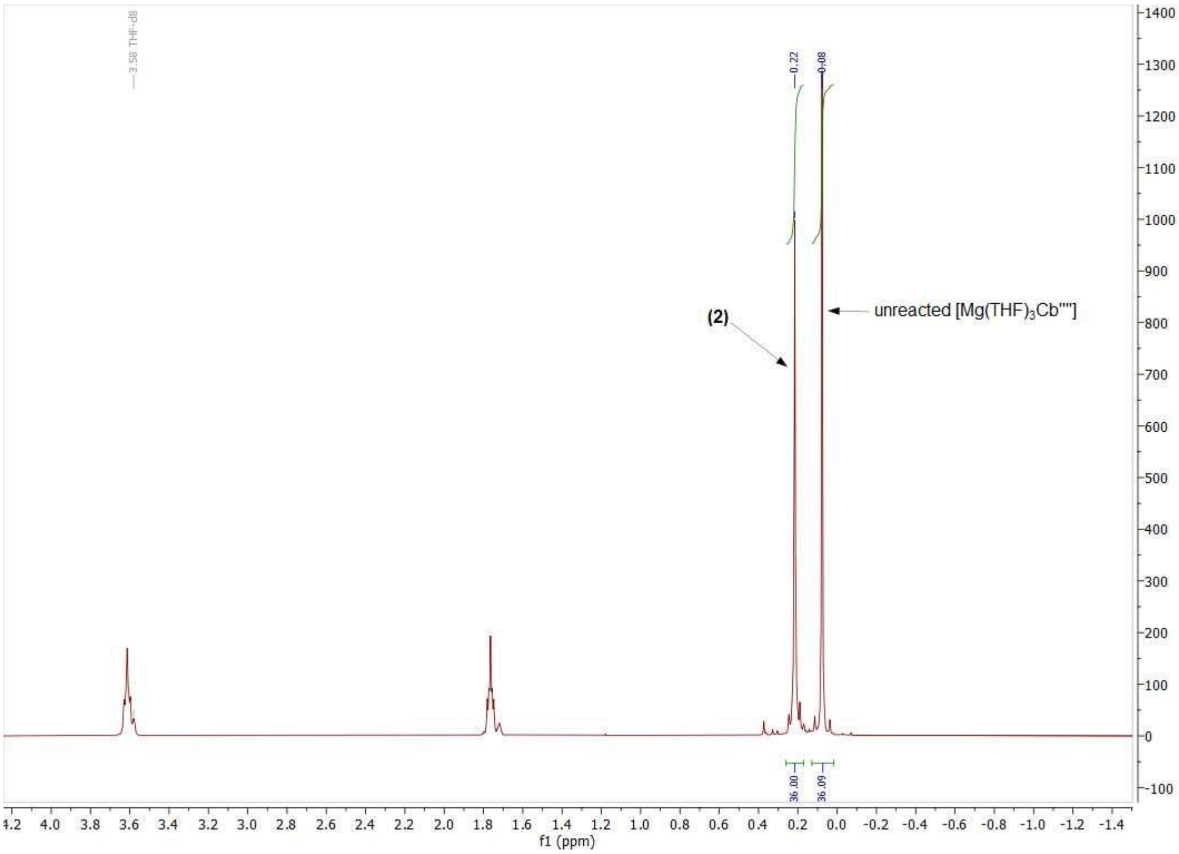

Figure S11. ^1H NMR spectrum of the 2:1 reaction of **1** and $ThCl_4$ before heating in THF-D_8.

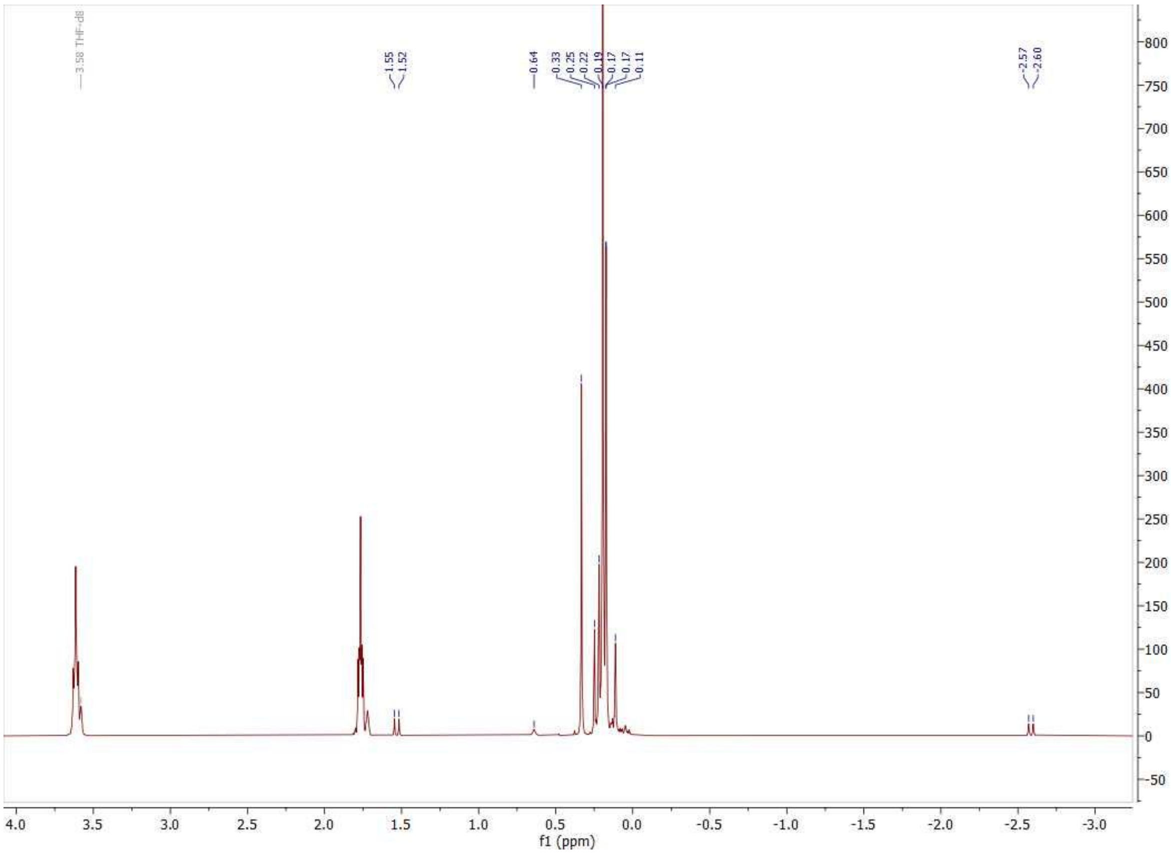

Figure S12. ^1H NMR spectrum of the 2:1 reaction of **1** and $ThCl_4$ after heating at 55°C overnight in THF-D_8.

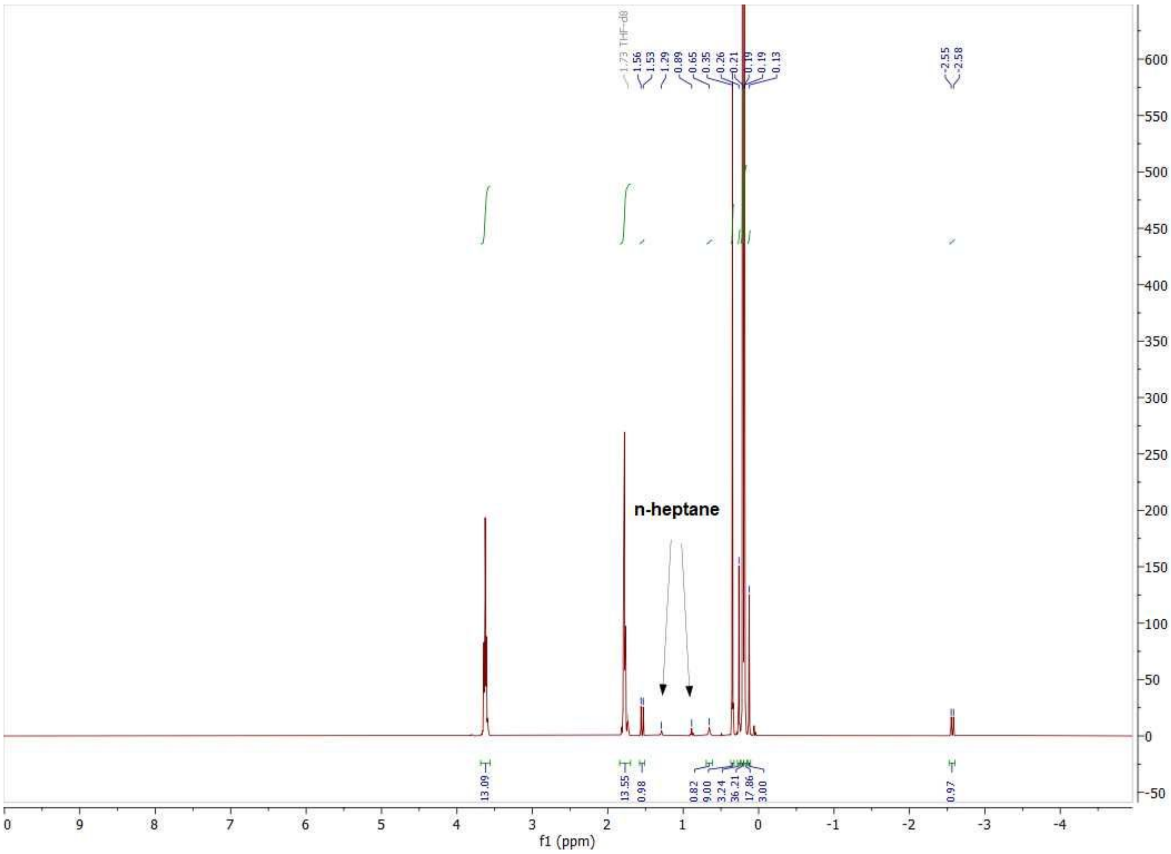

Figure S13. ^{1}H NMR spectrum of $[Mg_2Cl_3(THF)_6]$[**4**] in THF-D_8 at 30°C.

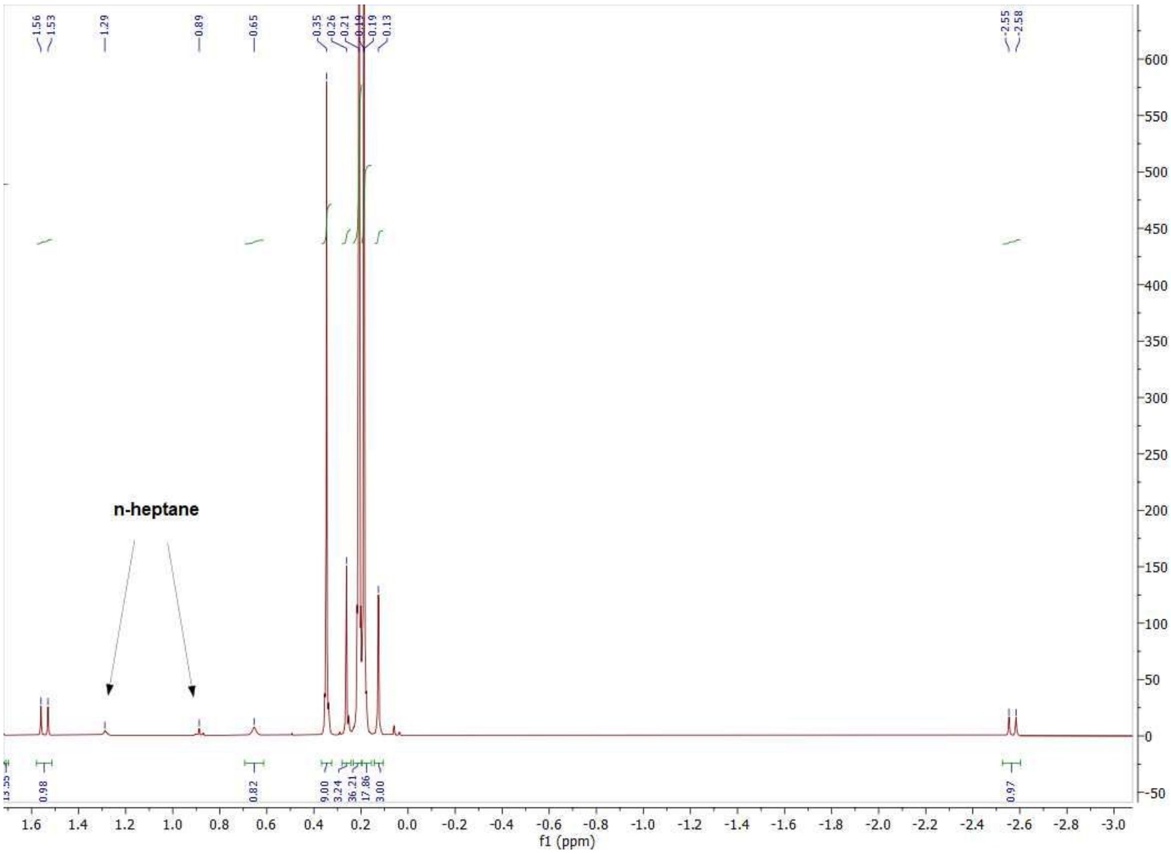

Figure S14. Expanded ^{1}H NMR spectrum of $[Mg_2Cl_3(THF)_6]$[**4**] in THF-D_8 at 30°C.

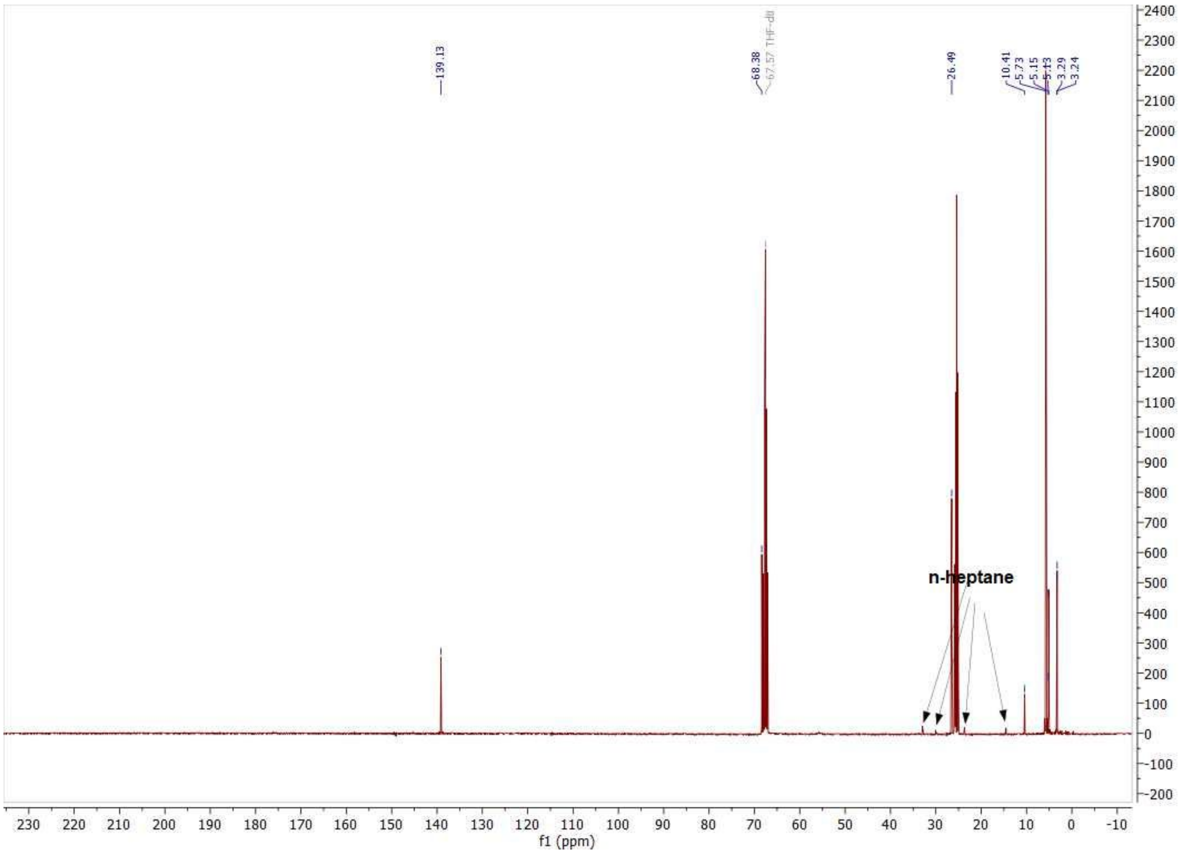

Figure S15. $^{13}C\{^{1}H\}$ NMR spectrum of $[Mg_2Cl_3(THF)_6][\mathbf{4}]$ in THF-D_8 at 30°C.

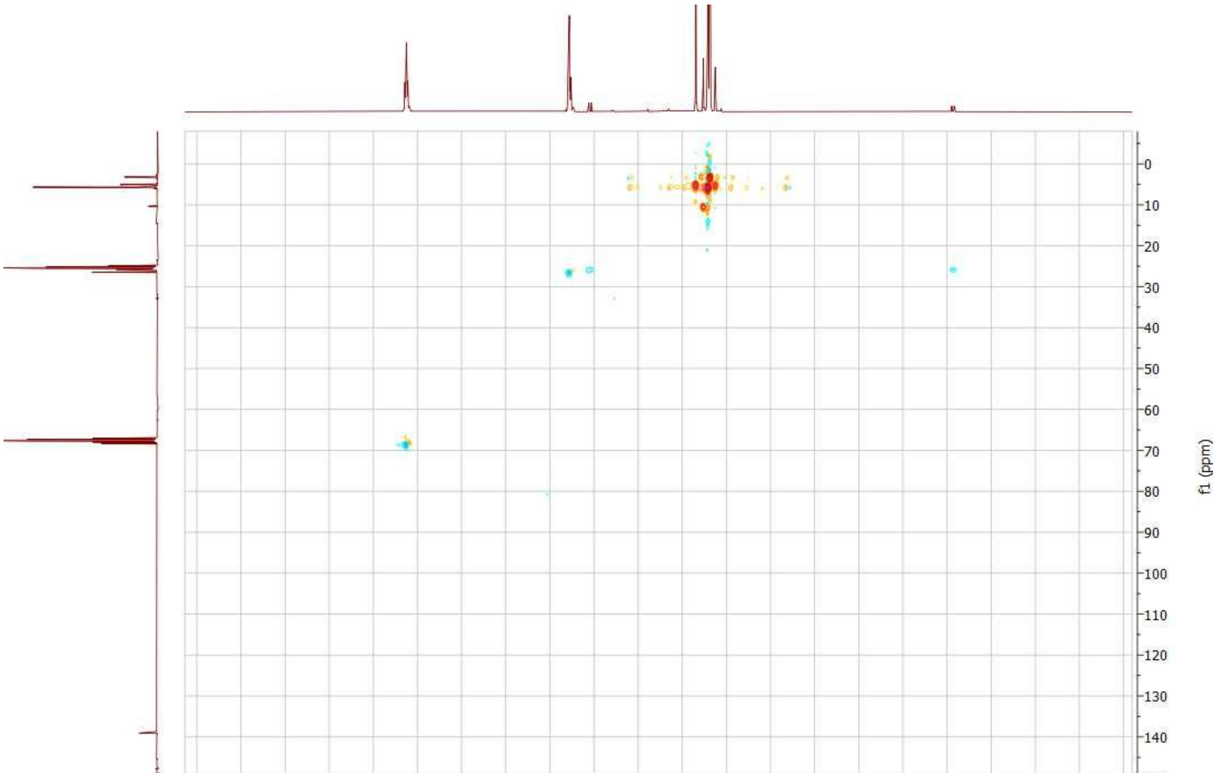

Figure S16. gHSQC ^{13}C-^{1}H correlation spectrum of $[Mg_2Cl_3(THF)_6][\mathbf{4}]$ in THF-D_8 at 30°C.

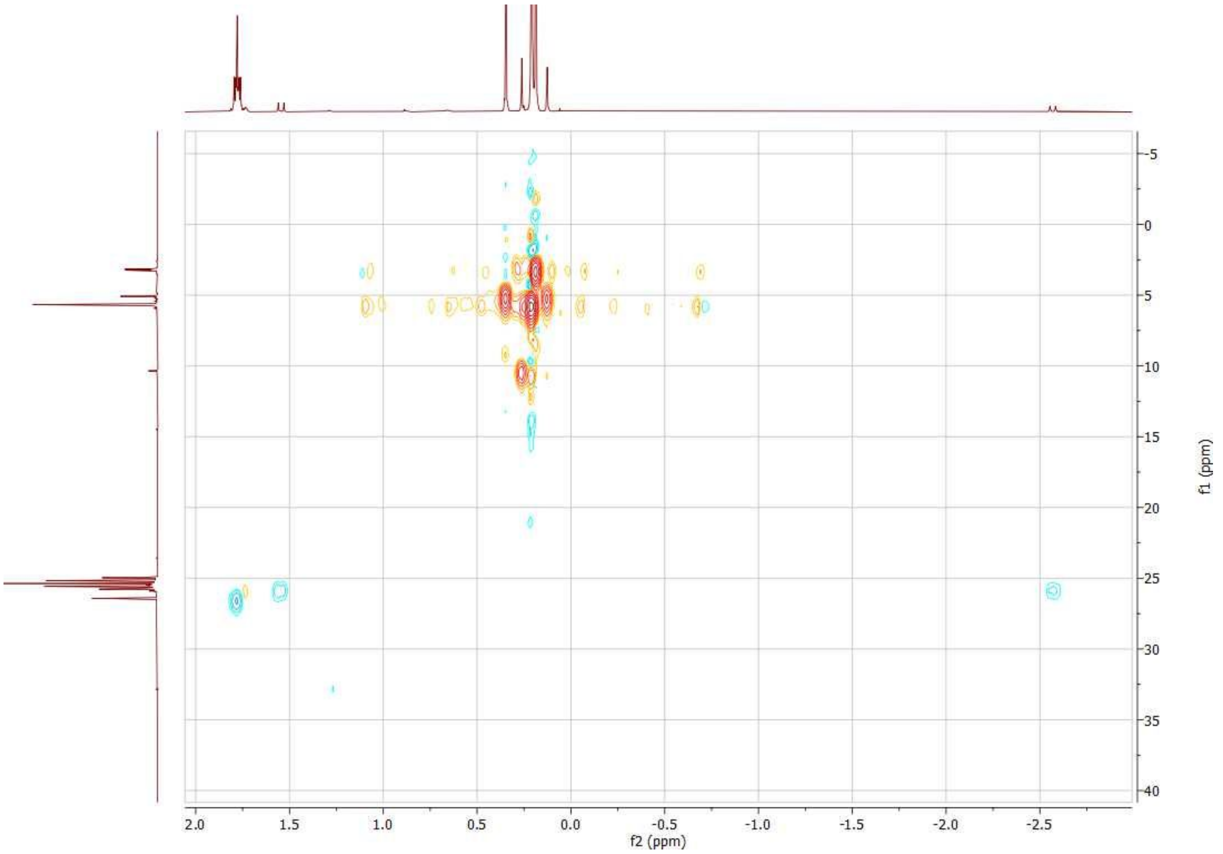

Figure S17. Expanded gHSQC ^{13}C-^{1}H correlation spectrum of $[Mg_2Cl_3(THF)_6]$[**4**] in THF-D_8 at 30°C.

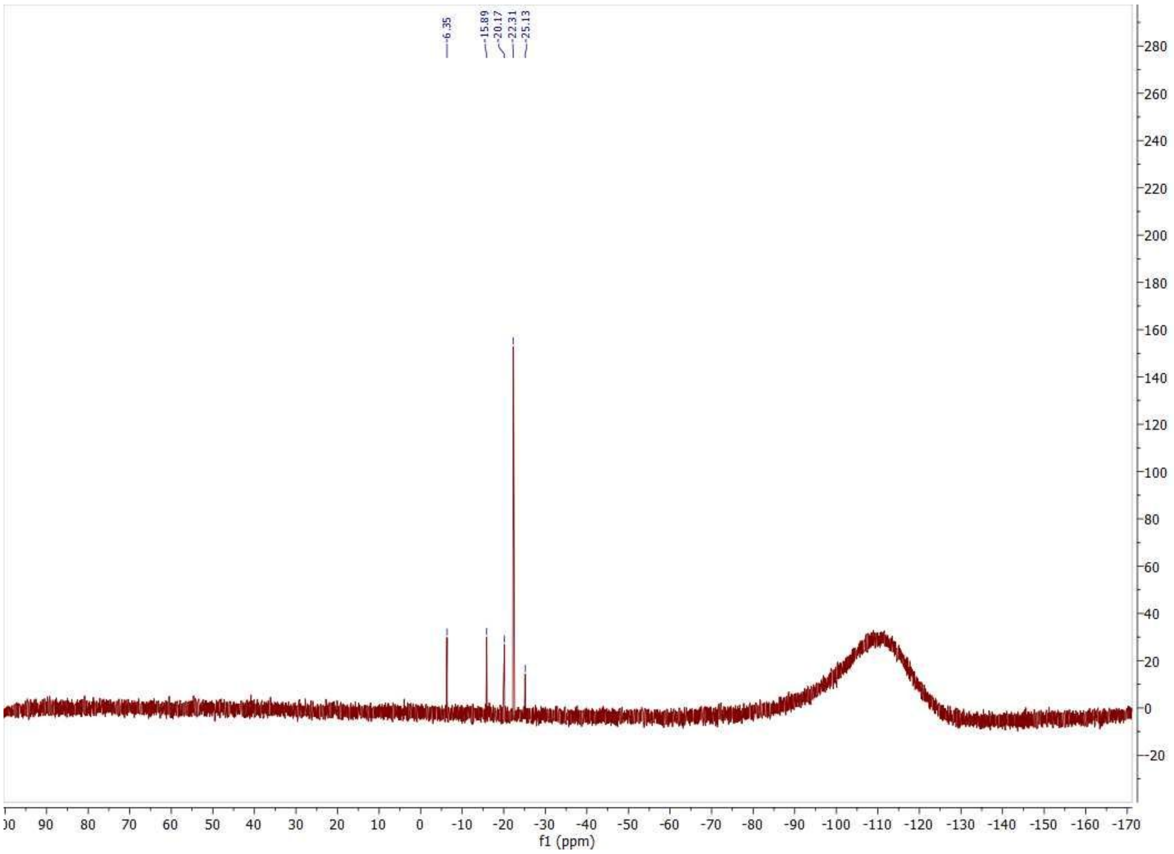

Figure S18. $^{29}Si\{^{1}H\}$ NMR spectrum of $[Mg_2Cl_3(THF)_6]$[**4**] in THF-D_8 at 30°C.

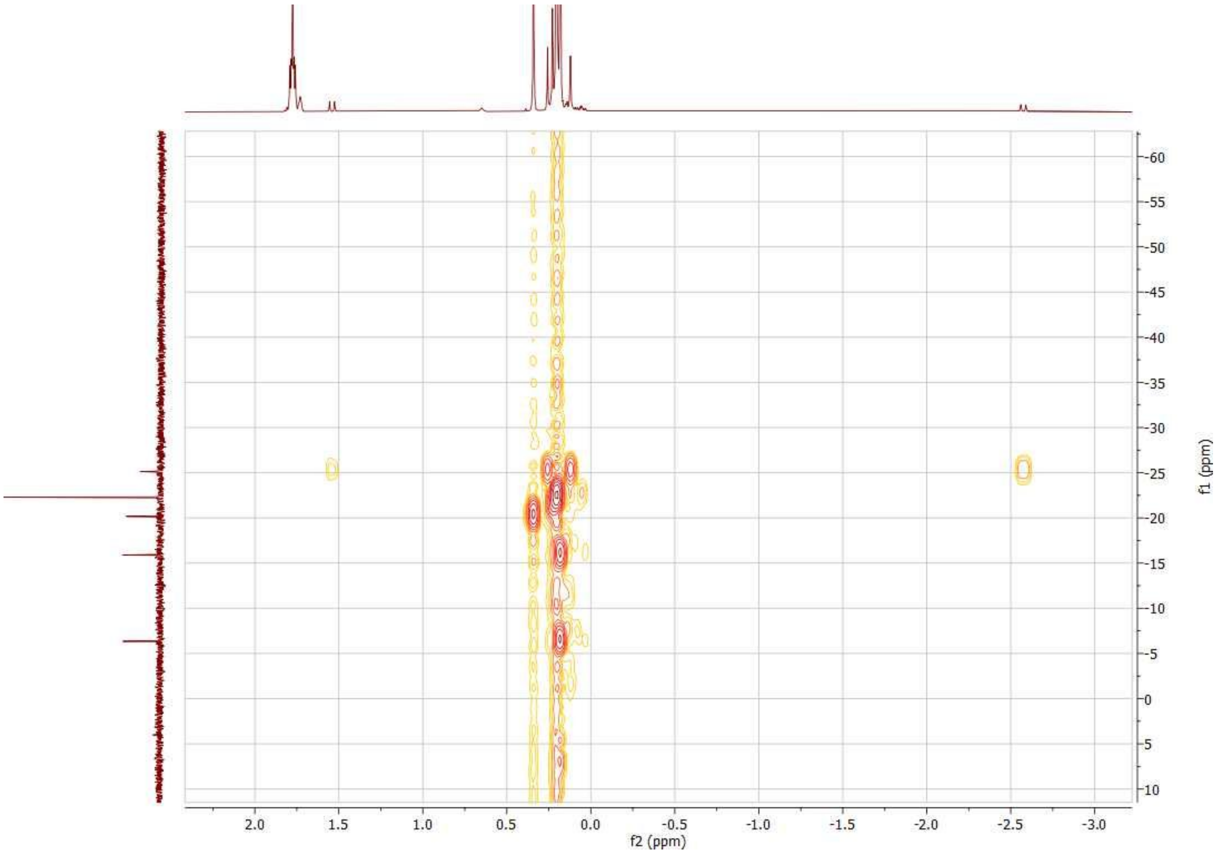

Figure S19. gHSQC ^{29}Si-^{1}H correlation spectrum of $[Mg_2Cl_3(THF)_6][\mathbf{4}]$ in THF-D_8 at 30°C.

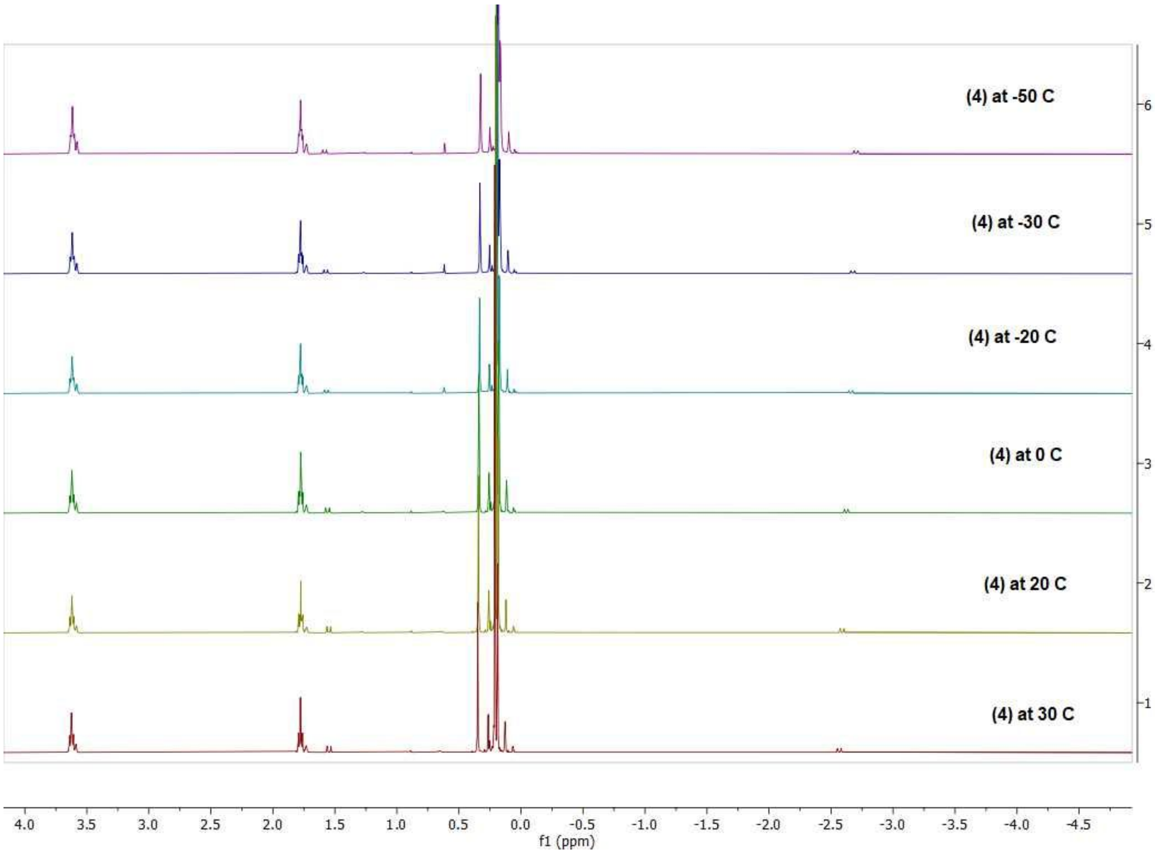

Figure S20. Variable-temperature ^{1}H NMR spectrum of $[Mg_2Cl_3(THF)_6][\mathbf{4}]$ in THF-D_8.

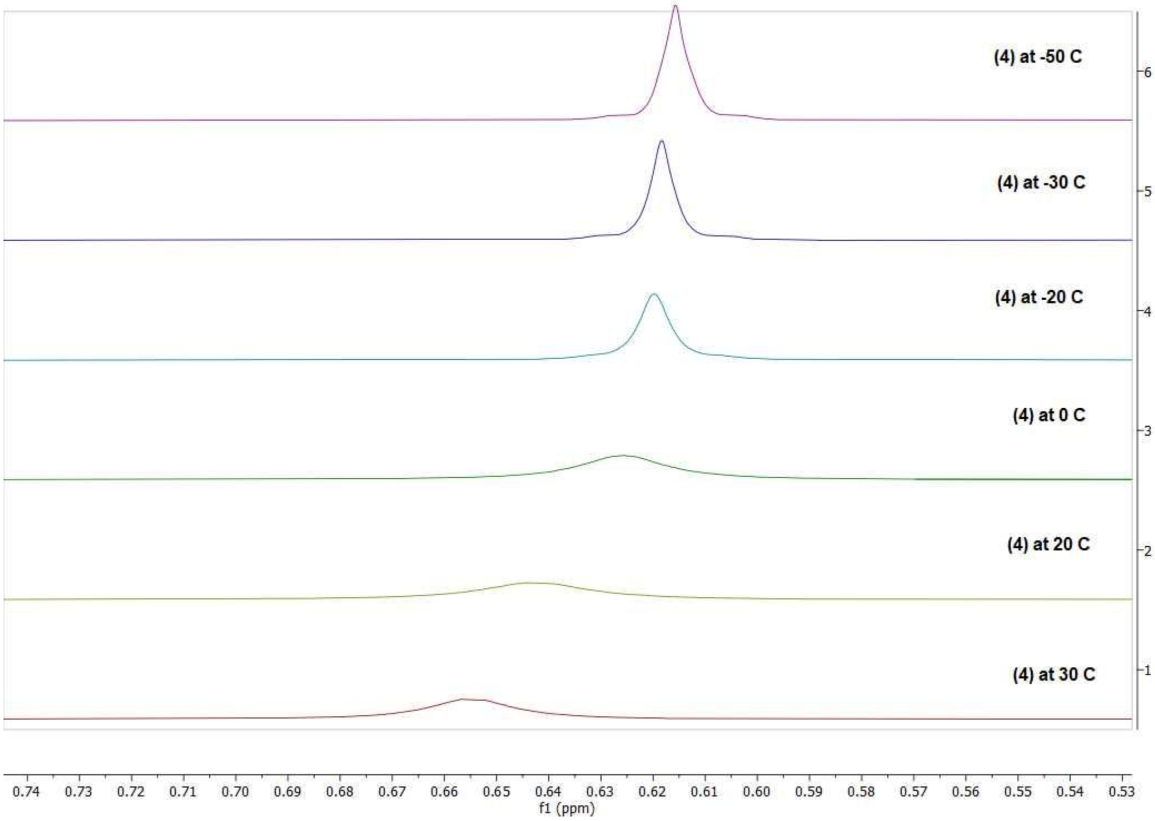

Figure S21. Variable-temperature ^{1}H NMR spectrum of $[Mg_2Cl_3(THF)_6][\mathbf{4}]$ in THF-D_8.

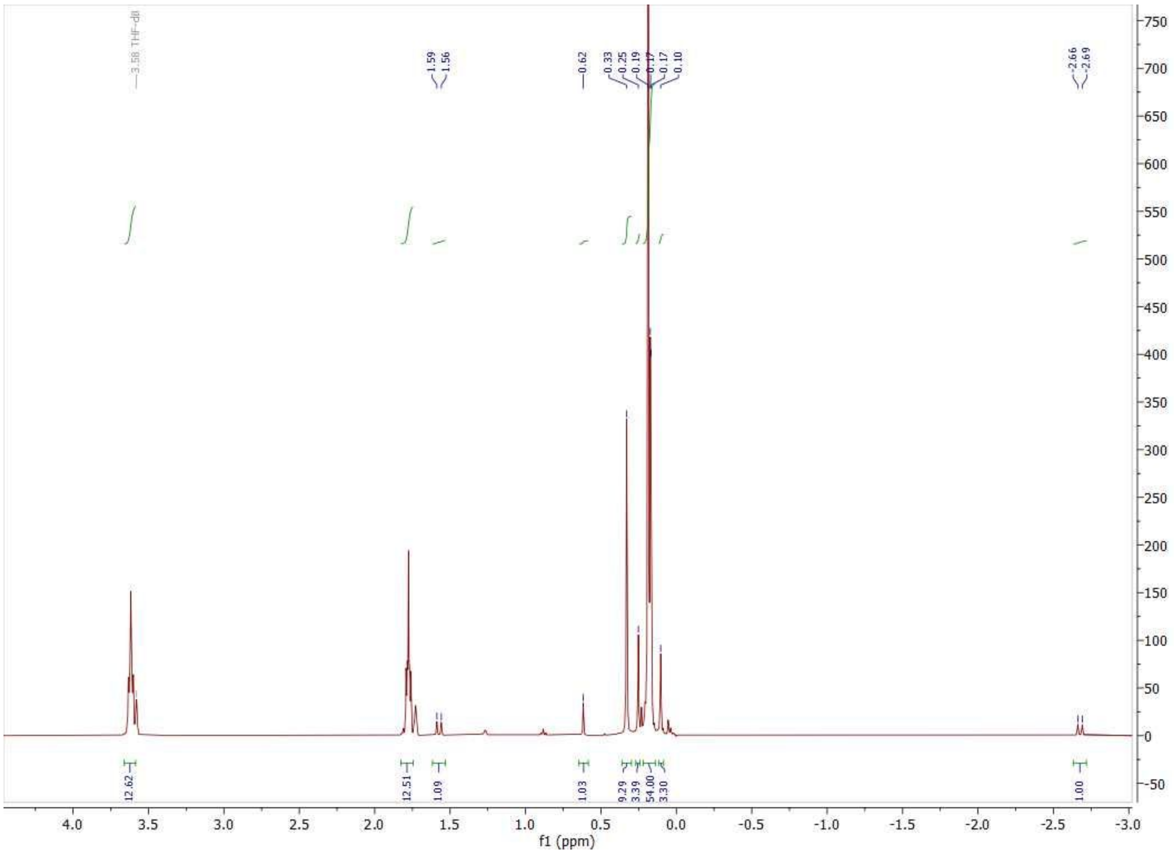

Figure S22. ^{1}H NMR spectrum of $[Mg_2Cl_3(THF)_6][\mathbf{4}]$ in THF-D_8 at –30°C.

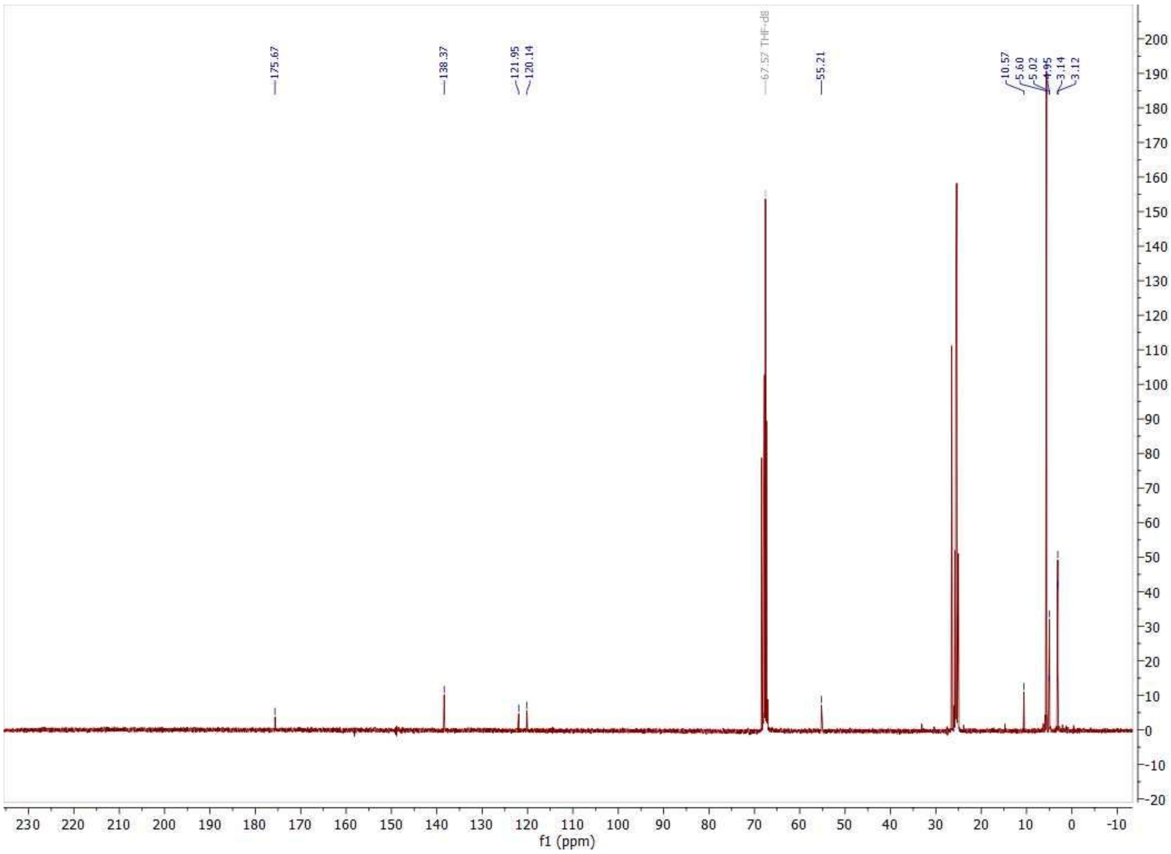

Figure S23. $^{13}C\{^{1}H\}$ NMR spectrum of $[Mg_2Cl_3(THF)_6]$[**4**] in THF-D_8 at –30°C.

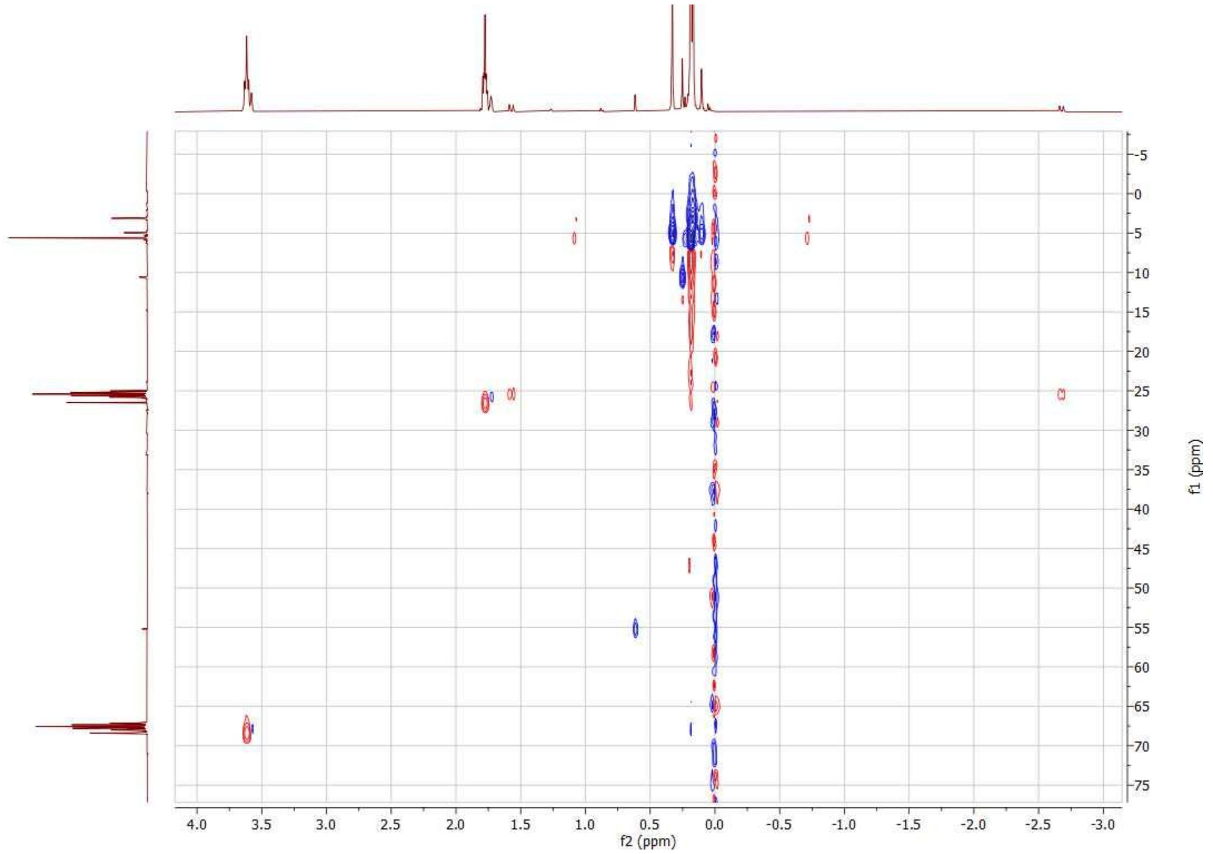

Figure S24. gHSQC (^{13}C-^{1}H) correlation spectrum of $[Mg_2Cl_3(THF)_6]$[**4**] in THF-D_8 at –30°C.

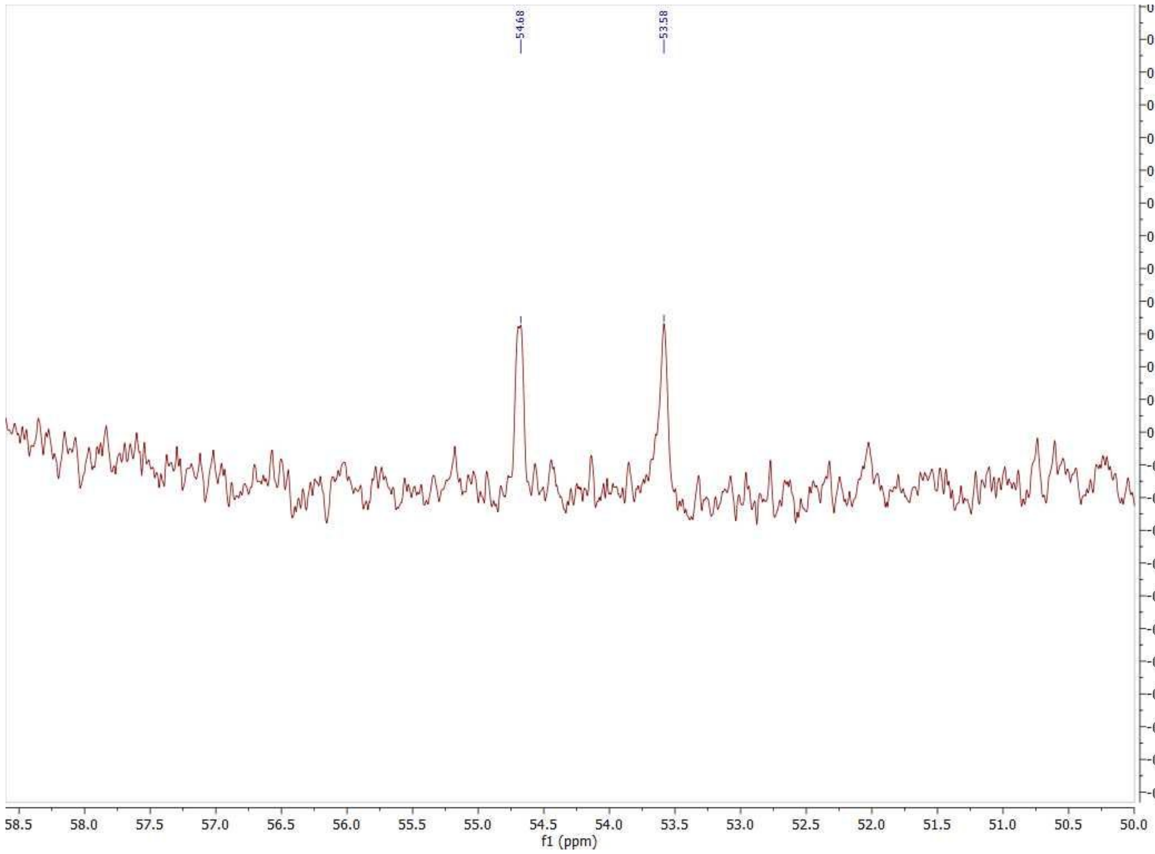

Figure S25. ^{1}H-coupled ^{13}C NMR spectrum of the methine carbon of $[Mg_2Cl_3(THF)_6][\mathbf{4}]$ in THF-D_8 at –30°C.

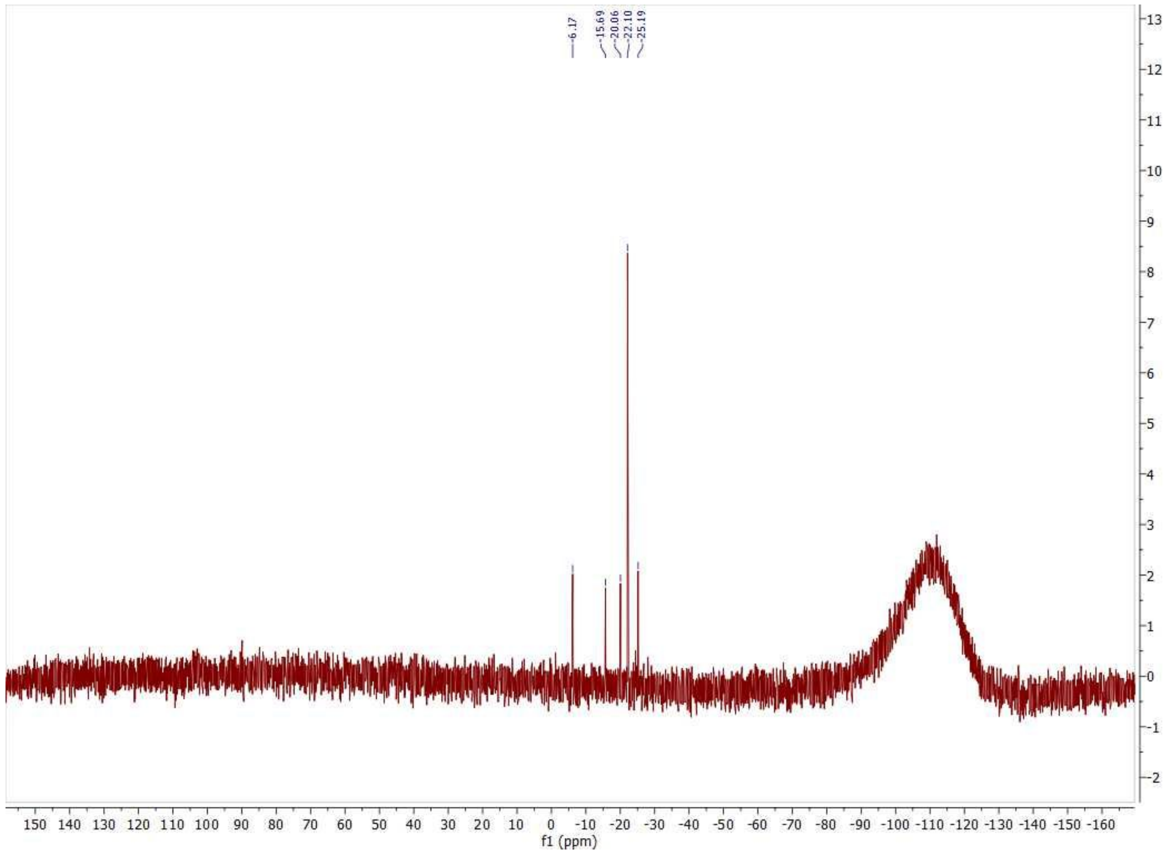

Figure S26. $^{29}Si\{^{1}H\}$ NMR spectrum of $[Mg_2Cl_3(THF)_6][\mathbf{4}]$ in THF-D_8 at –30°C.

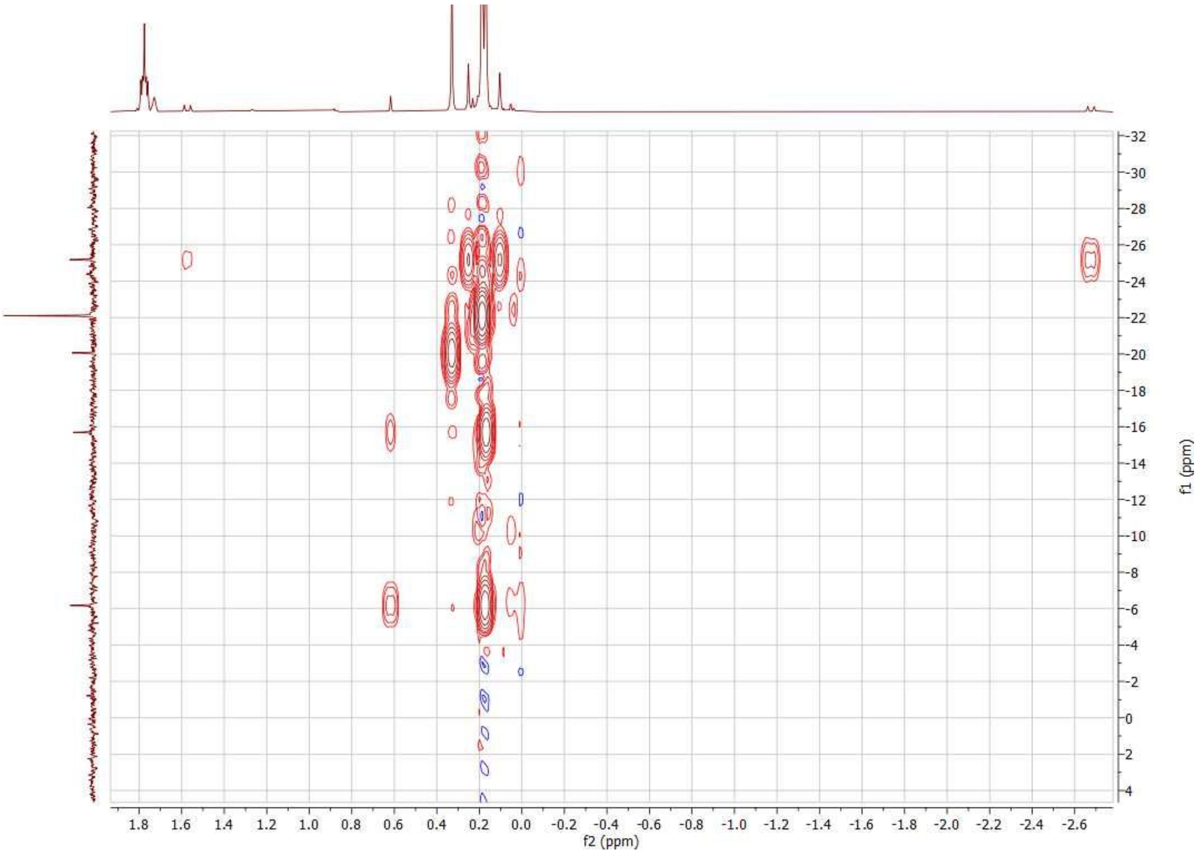

Figure S27. gHMBC ^{29}Si-^{1}H correlation spectrum of $[Mg_2Cl_3(THF)_6]$[**4**] in THF-D_8 at –30°C.

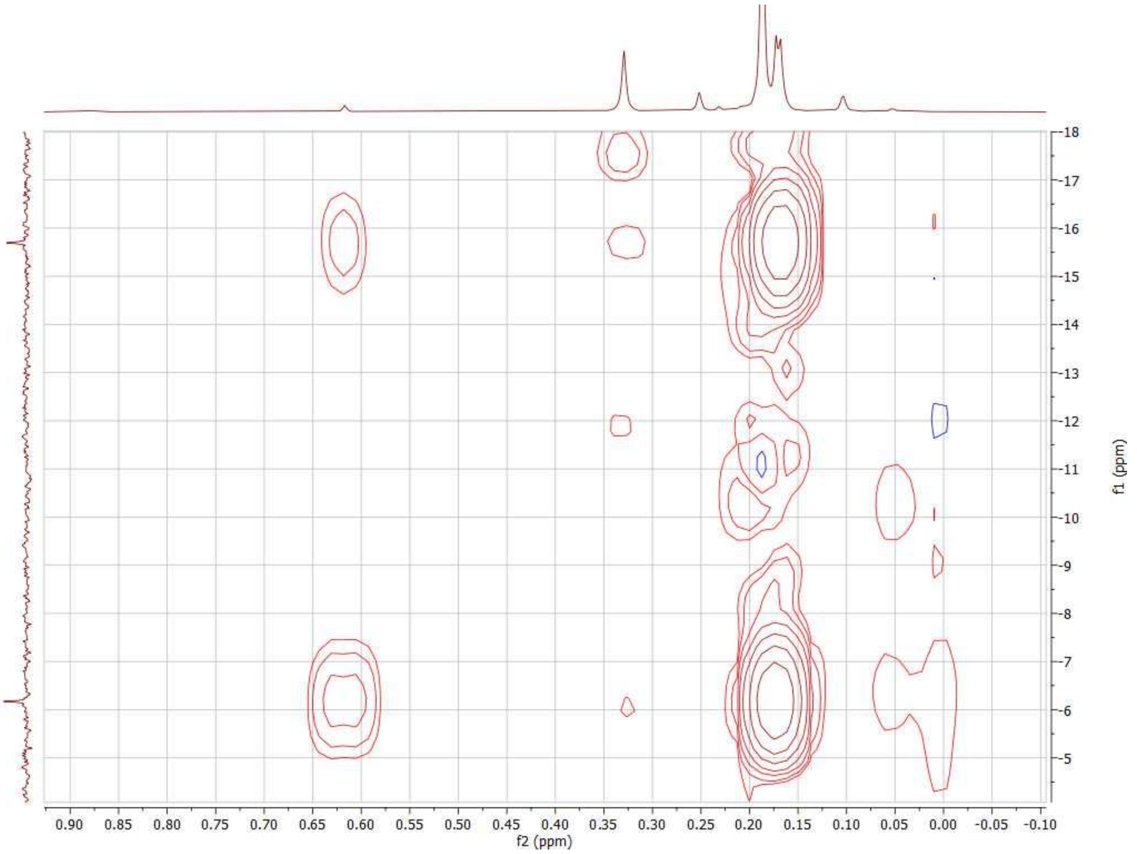

Figure S28. Expanded gHMBC ^{29}Si-^{1}H correlation spectrum of $[Mg_2Cl_3(THF)_6]$[**4**] in THF-D_8 at –30°C.

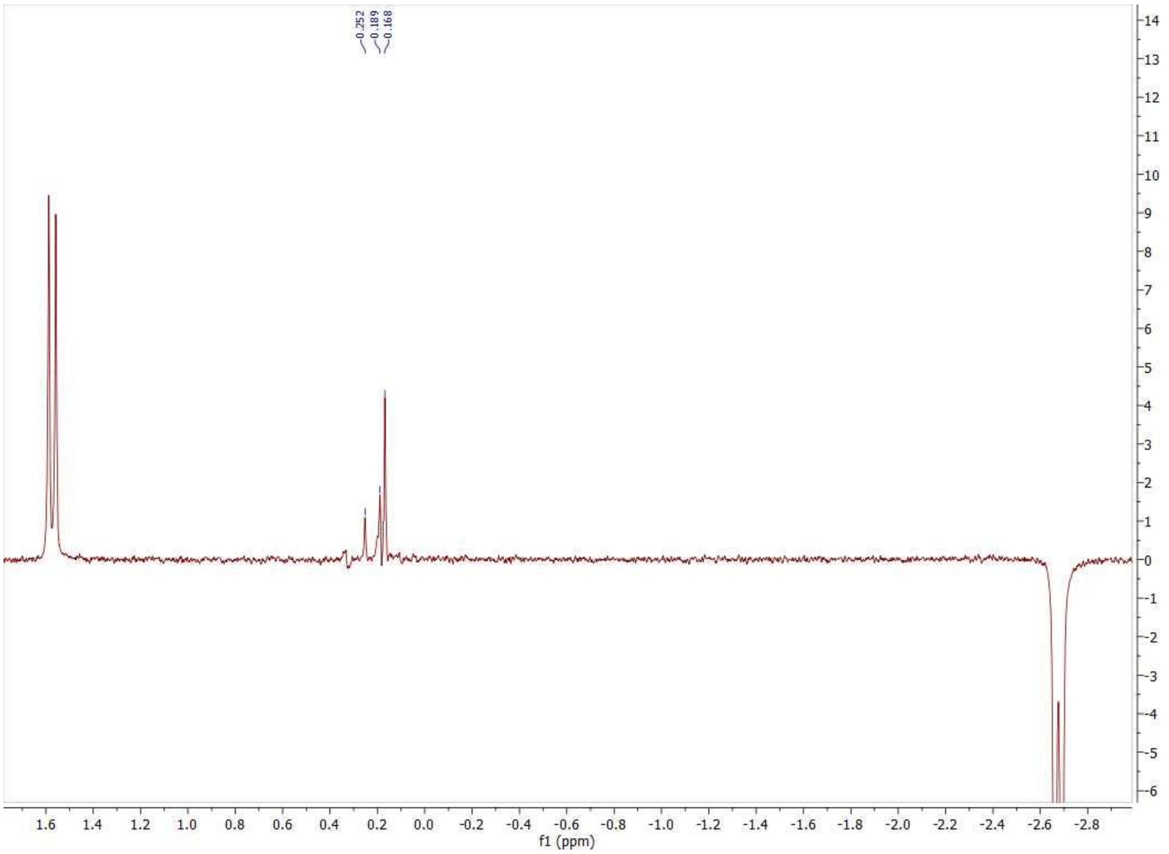

Figure S29. 1D NOESY spectrum of $[Mg_2Cl_3(THF)_6]$[**4**] THF-D_8 at –30°C, irradiating at δ = –2.67 ppm.

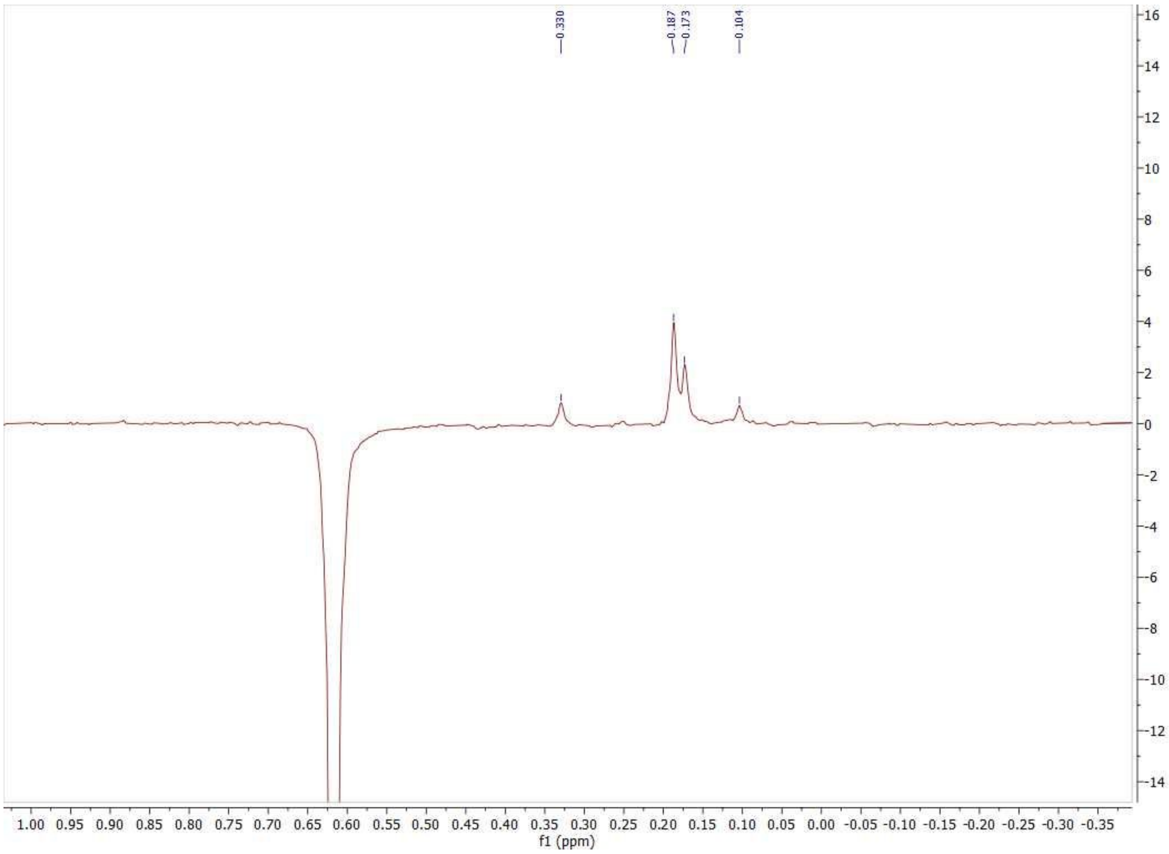

Figure S30. 1D NOESY spectrum of $[Mg_2Cl_3(THF)_6]$[**4**] THF-D_8 at –30°C, irradiating at δ = 0.62 ppm.

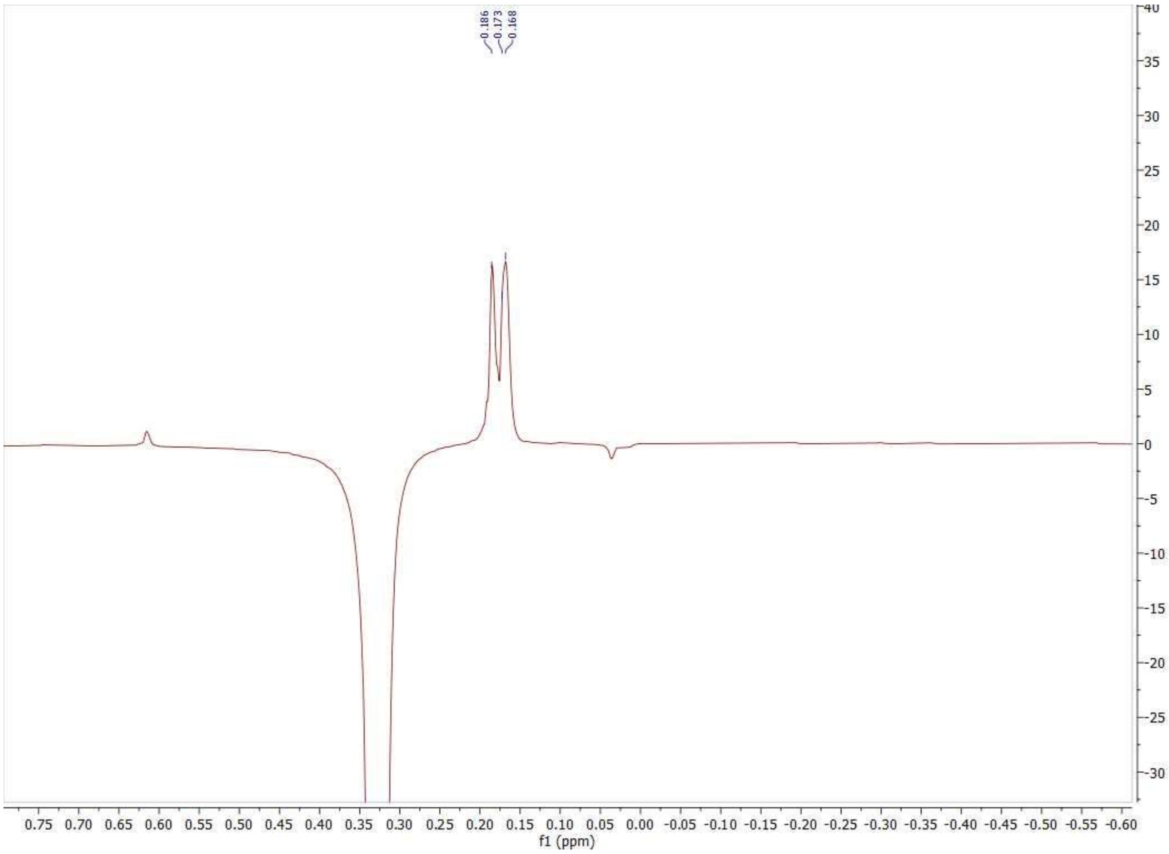

Figure S31. 1D NOESY spectrum of $[Mg_2Cl_3(THF)_6]$[**4**] THF-D_8 at –30°C, irradiating at δ = 0.33 ppm.

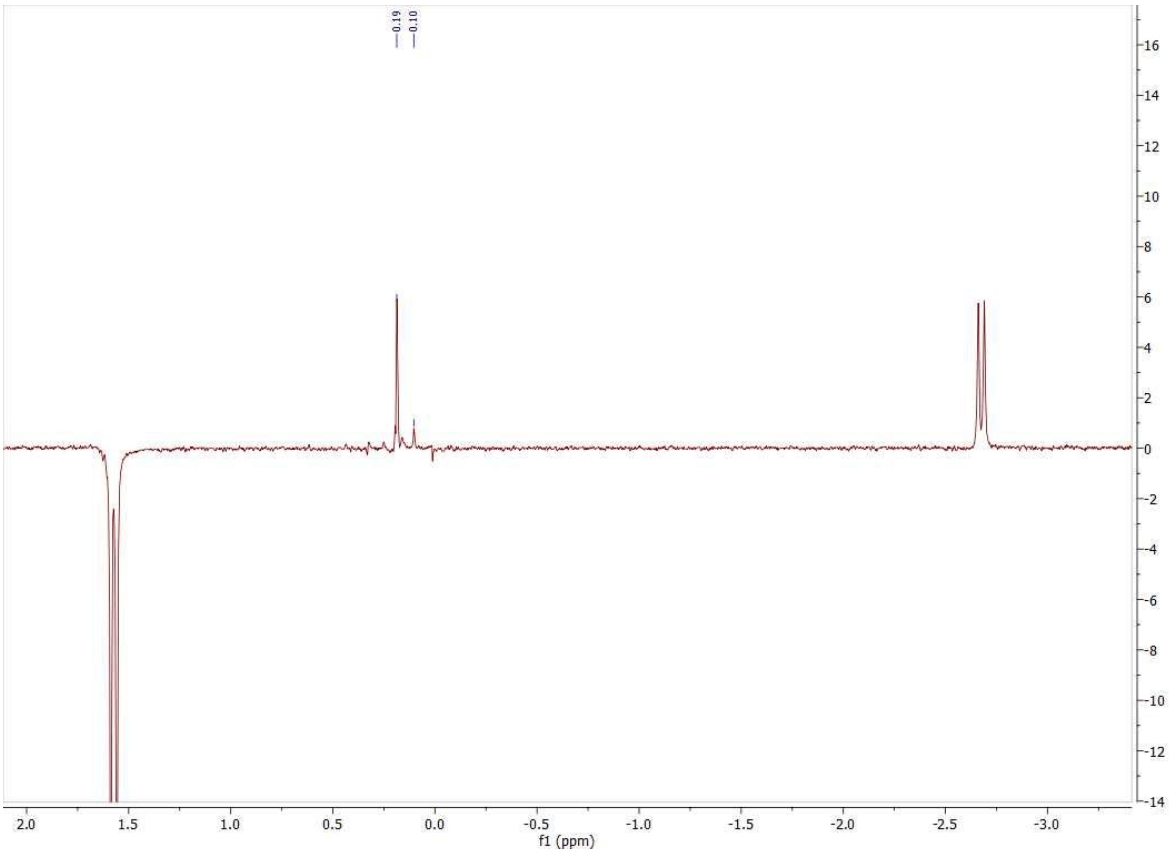

Figure S32. 1D NOESY spectrum of $[Mg_2Cl_3(THF)_6]$[**4**] THF-D_8 at –30°C, irradiating at δ = 1.57 ppm.

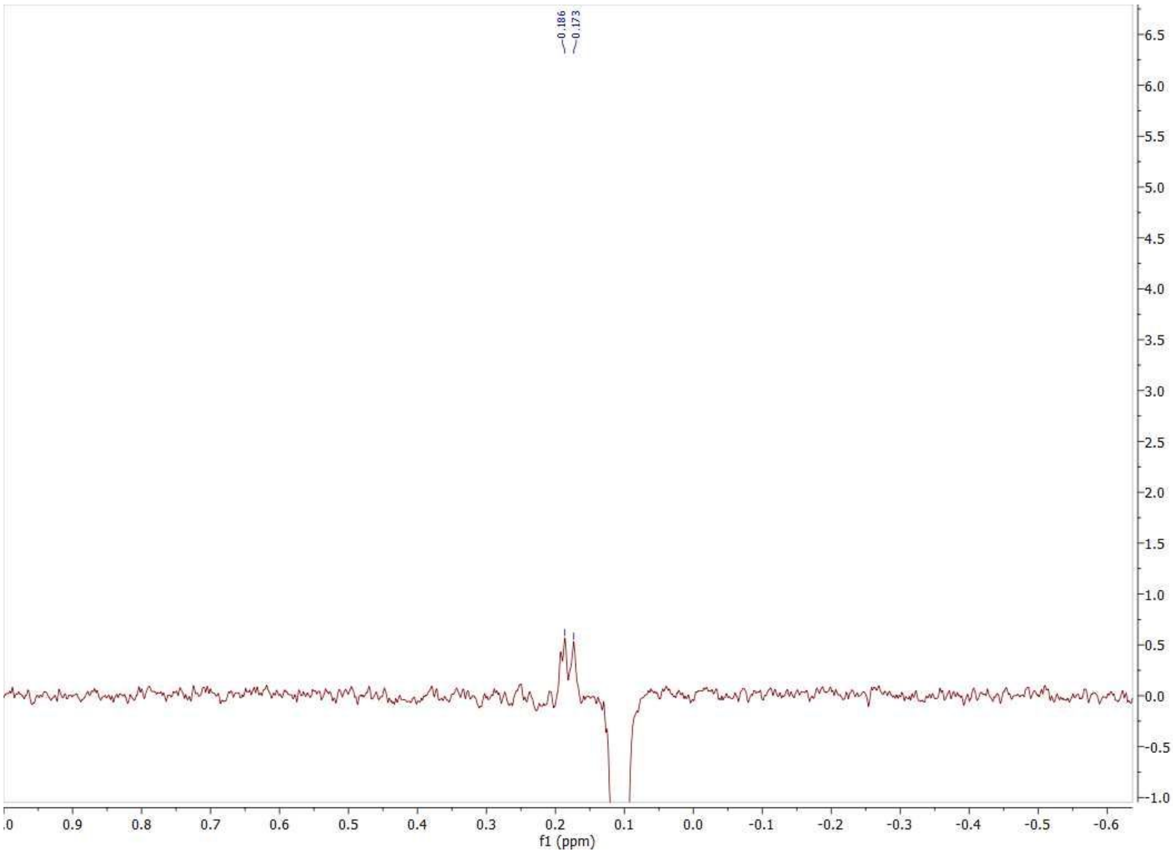

Figure S33. 1D NOESY spectrum of $[Mg_2Cl_3(THF)_6]$[**4**] THF-D_8 at –30°C, irradiating at δ = 0.10 ppm.

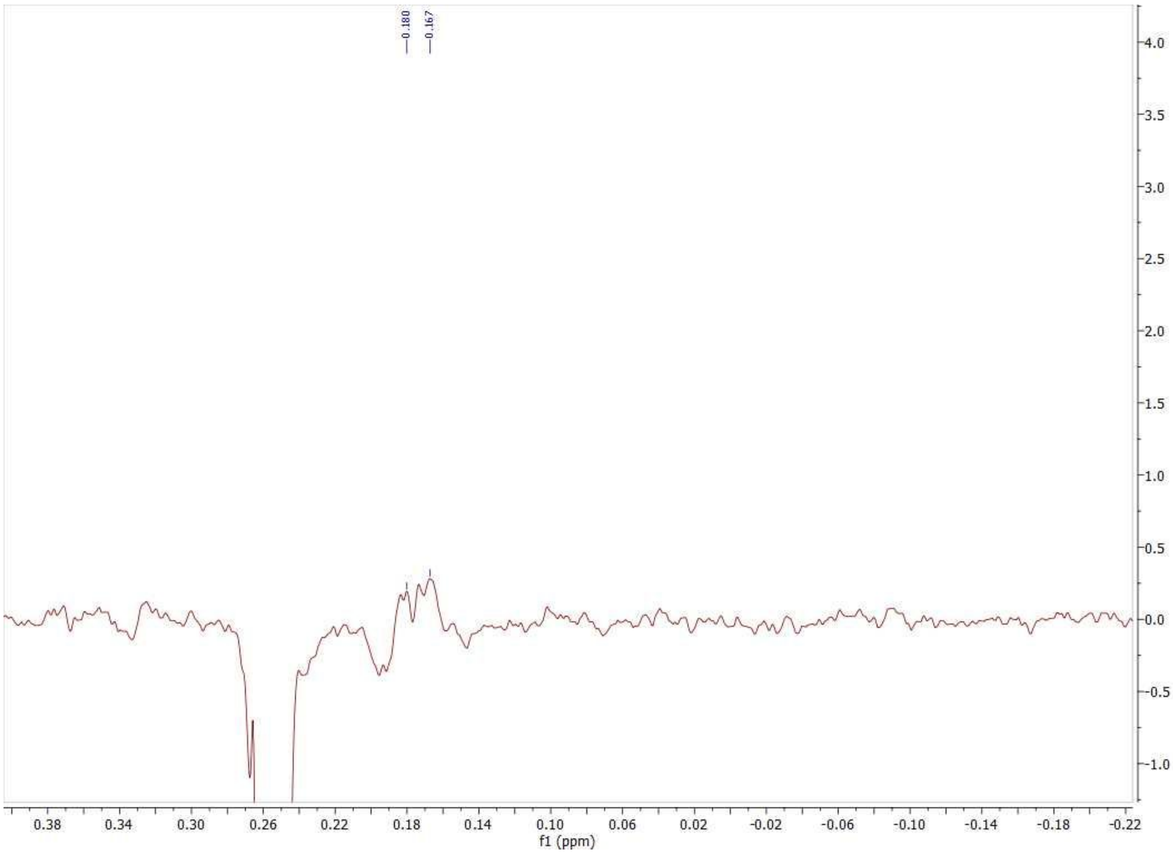

Figure S34. 1D NOESY spectrum of $[Mg_2Cl_3(THF)_6]$[**4**] THF-D_8 at –30°C, irradiating at δ = 0.25 ppm.

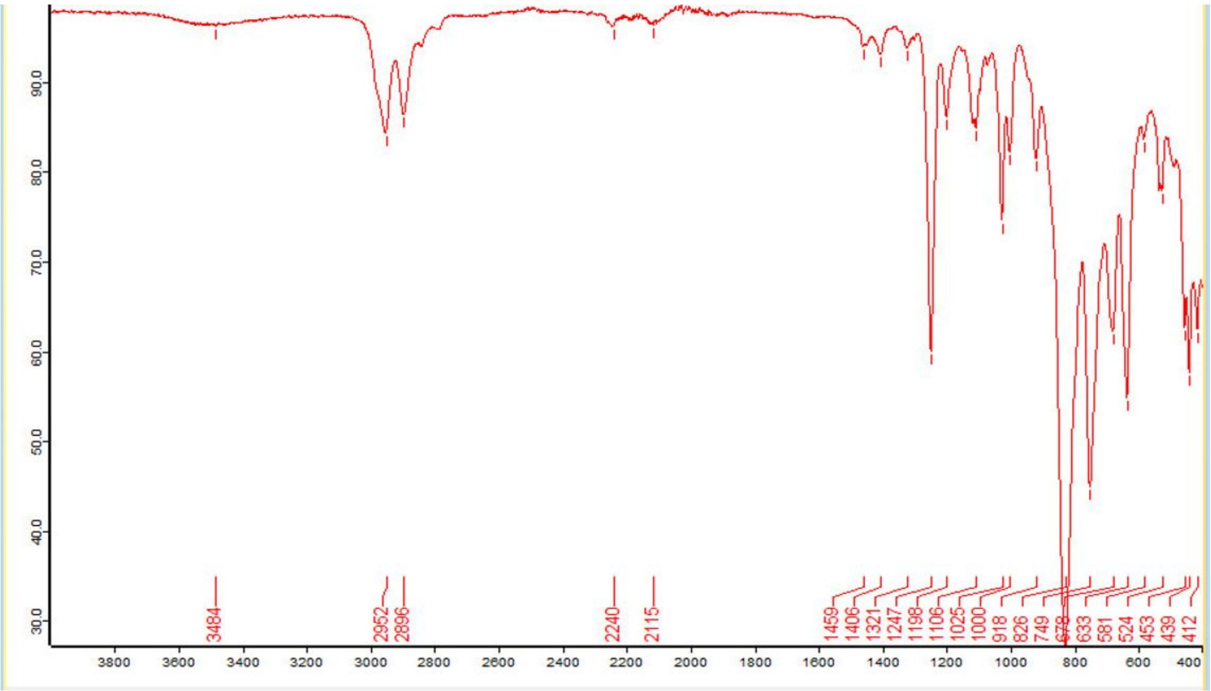

Figure S35. IR spectrum of $[Mg_2Cl_3(THF)_6]$[**4**].

Synthesis of $[Mg_2Cl_3(THF)_6][(\eta^4\text{-Cb''''})U(\eta^3\text{-}C_4H(SiMe_3)_3\text{-}\kappa\text{-}(CH_2SiMe_2)(Cl)]$ ($[Mg_2Cl_3(THF)_6][\mathbf{5}]$)

This compound was synthesized using the method described for $[Mg_2Cl_3(THF)_6]$[**4**] UCl_4 (80 mg, 0.21 mmol) and **1** (245 mg, 0.42 mmol). In this case, no heating was required, and after three hours at room temperature a dark brown solution was obtained. Work-up and recrystallization gave $[Mg_2Cl_3(THF)_6]$[**4**] (170 mg, 52%).

NMR data at +30°C

Major species $[Mg_2Cl_3(THF)_6]$[5]. ^{1}H NMR (δ/ppm, THF-D_8): 15.76 (s, 3H, diastereotopic CH_3); 10.73 (s, 9H, $SiMe_3$); 6.56 (s, 9H, $SiMe_3$); –1.65 (s, 36H, $SiMe_3$); –8.09 (s, 9H, $SiMe_3$); –23.81 (s, 3H, diastereotopic CH_3); –77.35 (s, 1H); –108.62 (s, 1H); –140 (s, 1H). **^{29}Si{^{1}H} NMR** (δ/ppm, THF-D_8): 127.22, 59.90, –103.98, –176.81, –199.45 (assigned to the major species in solution owing to their relatively high intensity). **^{25}Mg{^{1}H} NMR** (δ/ppm, THF-D_8): 2.95 (s, FWHM = 97.0 Hz).

Minor species (approx. 7%). ^{1}H NMR (δ/ppm, THF-D_8): 15.17, –0.69, –9.63 (s, relative integrations 1:9:3). **^{29}Si{^{1}H} NMR** (δ/ppm, THF-D_8): 81.18, –152.79 (assigned to the minor species in solution owing to their relatively low intensity).

NMR data at –77°C

Major species $[Mg_2Cl_3(THF)_6]$[5]. ^{1}H NMR (δ/ppm, THF-D_8): 22.97 (s, 3H, diastereotopic CH_3); 18.58 (s, 9H, $SiMe_3$); 12.17 (s, 9H, $SiMe_3$); –2.62 (s, 36H, $SiMe_3$, FWHM = 1065.8 Hz); –42.12 (s, 3H, diastereotopic CH_3); –141.62 (s, 1H); –163.68 (s, 1H); –243.88 (s, 1H). **^{29}Si{^{1}H} NMR** (δ/ppm, THF-D_8): 284.79, 110.11, –166.66, –227.35, –312.19 (assigned to the major species in solution owing to their relatively high intensity). **^{25}Mg{^{1}H} NMR** (δ/ppm, THF-D_8): no observable signals.

Minor species (approx. 7%). ^{1}H NMR (δ/ppm, THF-D_8): 53.43 (s, 2 × 3H, $Me_2SiC\underline{H_3}\cdots U$); 25.11 (9H, s, $SiMe_3$); 24.57 (s, 18H, 2 × $SiMe_3$); –8.99 (s, 18H, 2 × $SiMe_3$); –14.31 (9H, s, $SiMe_3$); –60.44 (s, 2 × 6H, $\underline{Me_2}SiCH_3\cdots U$). **^{29}Si{^{1}H} NMR** (δ/ppm, THF-D_8): 340.75, 143.80, –236.10 (assigned to the minor species in solution owing to their relatively low intensity). **^{25}Mg{^{1}H} NMR** (δ/ppm, THF-D_8): no observable signals.

Elemental Analysis: Calculated for $C_{56}H_{120}Cl_4Mg_2O_6Si_8U$: C 43.60, H 7.84. Found: C 43.35, H 7.75.

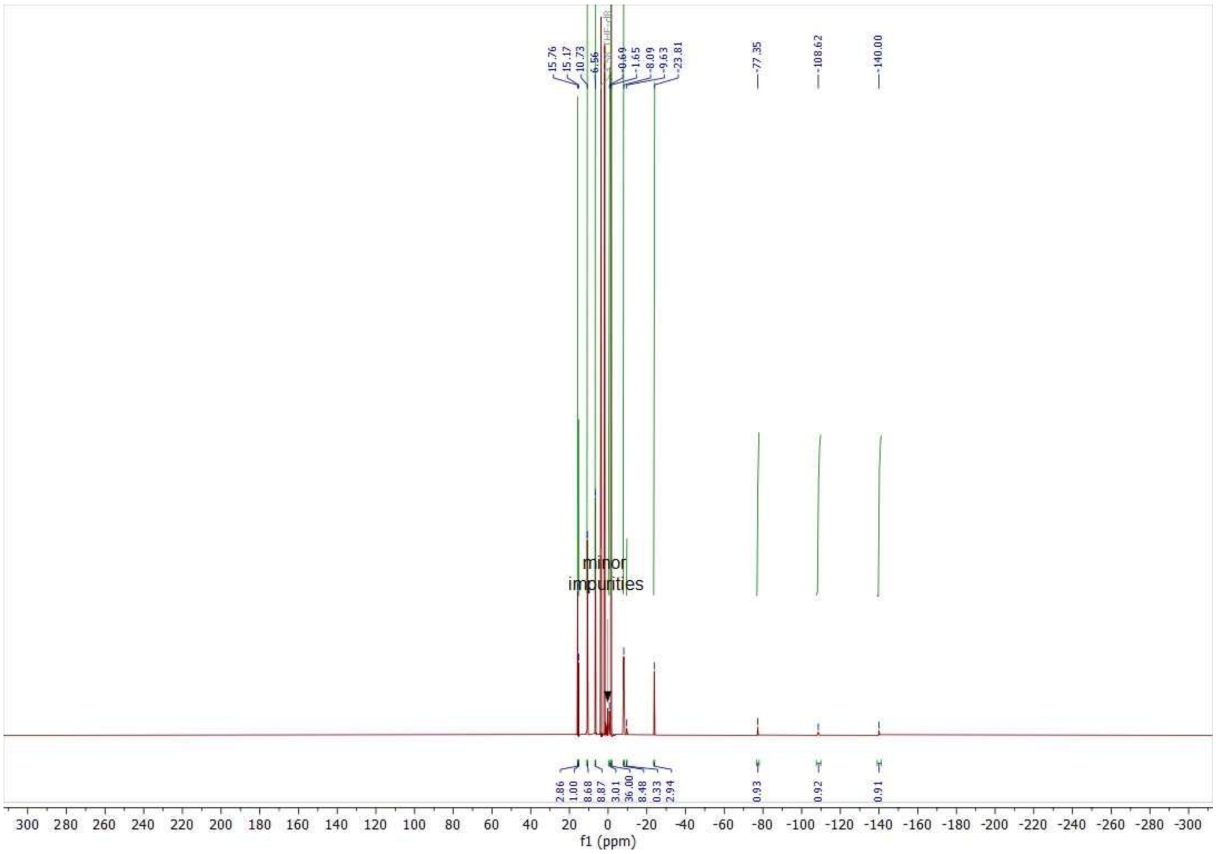

Figure S36. ^{1}H NMR spectrum of $[Mg_2Cl_3(THF)_6]$[**5**] in THF-D_8 at 30°C.

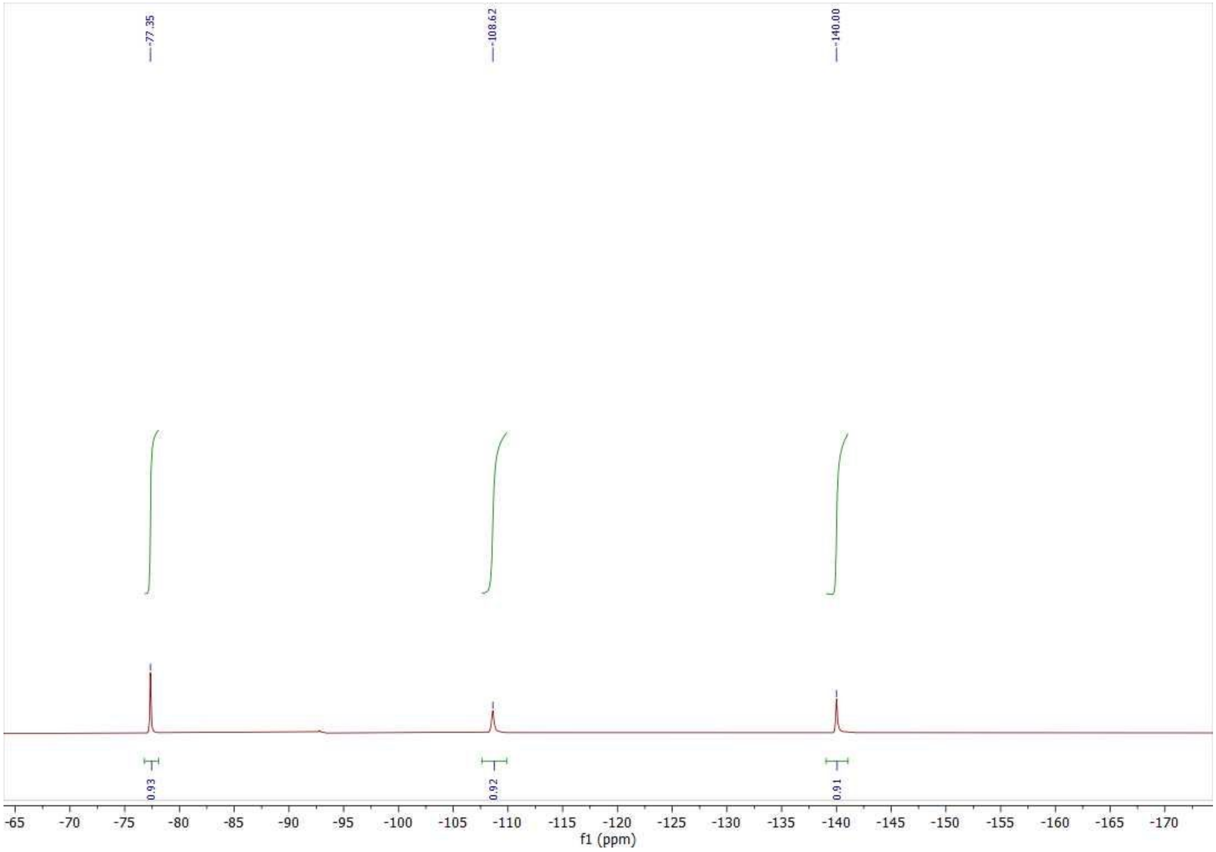

Figure S37. Expanded ^{1}H NMR spectrum of $[Mg_2Cl_3(THF)_6]$[**5**] in THF-D_8 at 30°C.

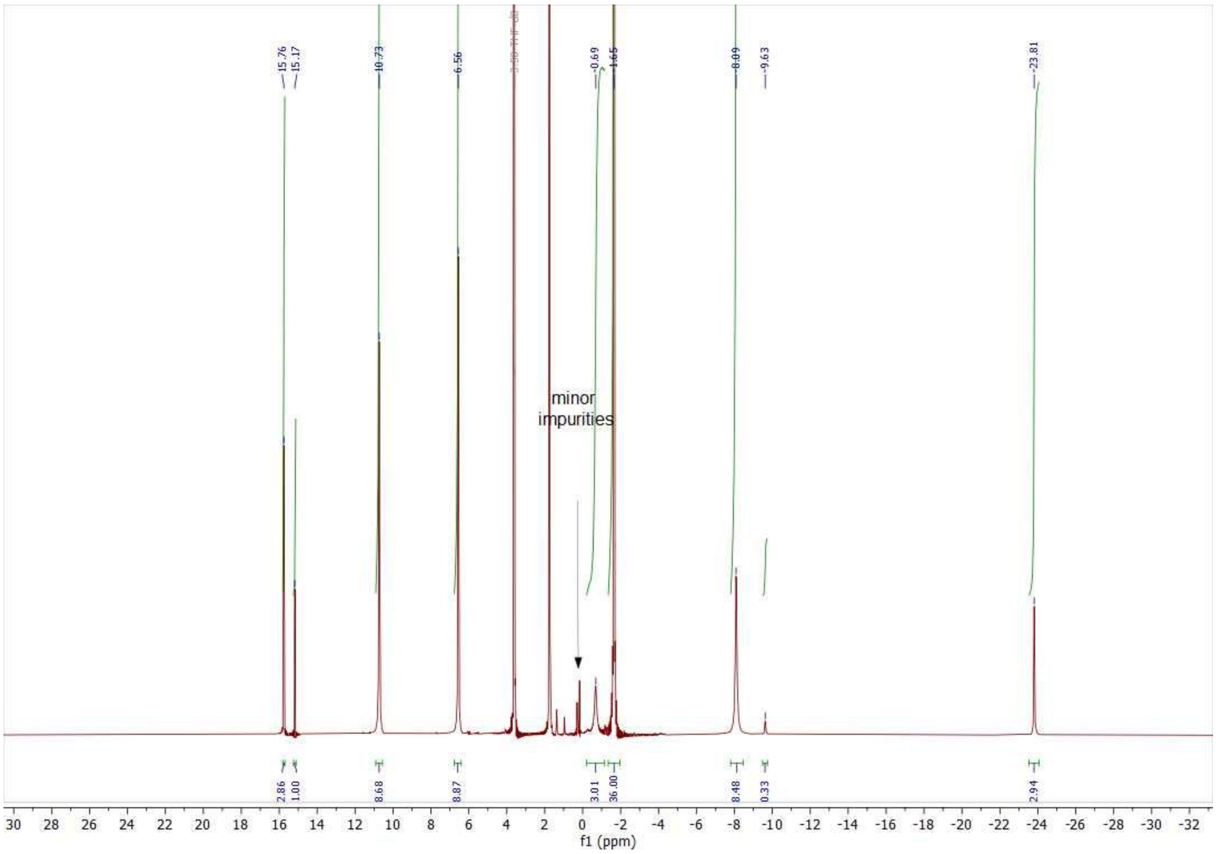

Figure S38. Expanded ^{1}H NMR spectrum of $[Mg_2Cl_3(THF)_6][\mathbf{5}]$ in THF-D_8 at 30°C.

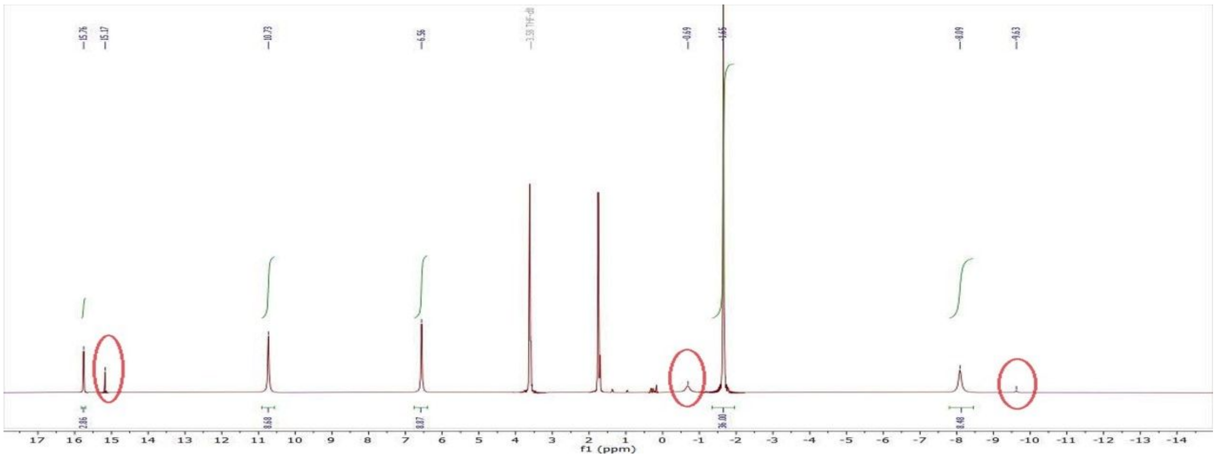

Figure S39. Expanded ^{1}H NMR spectrum of $[Mg_2Cl_3(THF)_6][\mathbf{5}]$ in THF-D_8 at 30°C. Highlighted peaks correspond to the minor species.

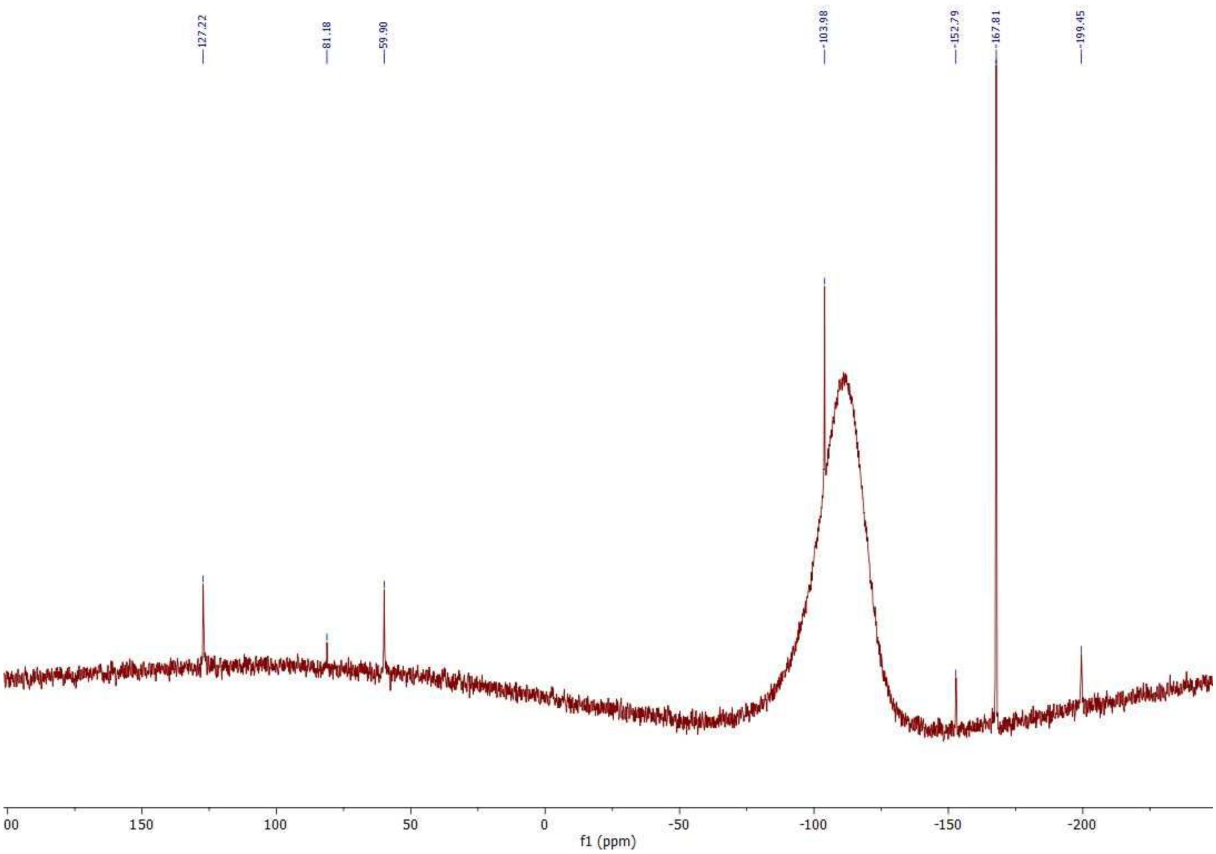

Figure S40. $^{29}Si\{^1H\}$ NMR spectrum of $[Mg_2Cl_3(THF)_6][\mathbf{5}]$ in THF-D_8 at 30°C.

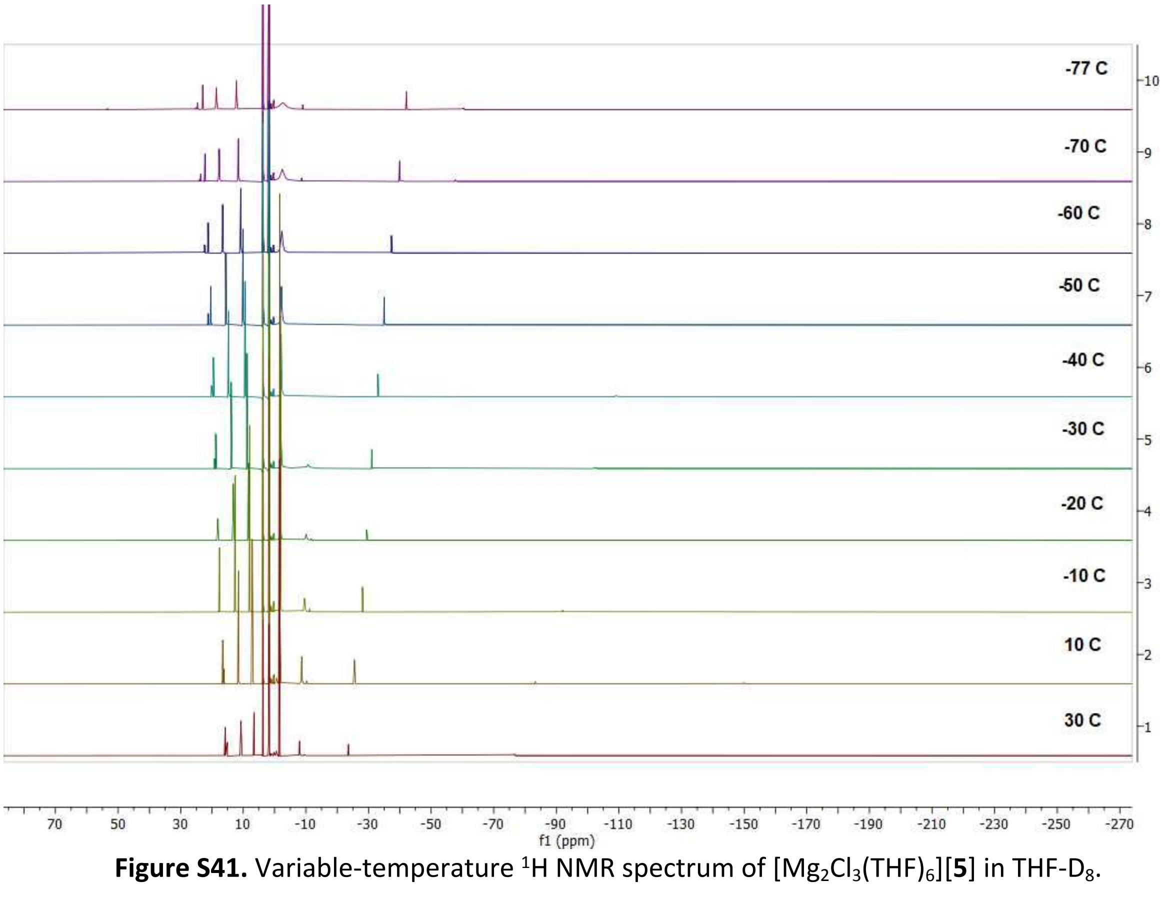

Figure S41. Variable-temperature ^1H NMR spectrum of $[Mg_2Cl_3(THF)_6][\mathbf{5}]$ in THF-D_8.

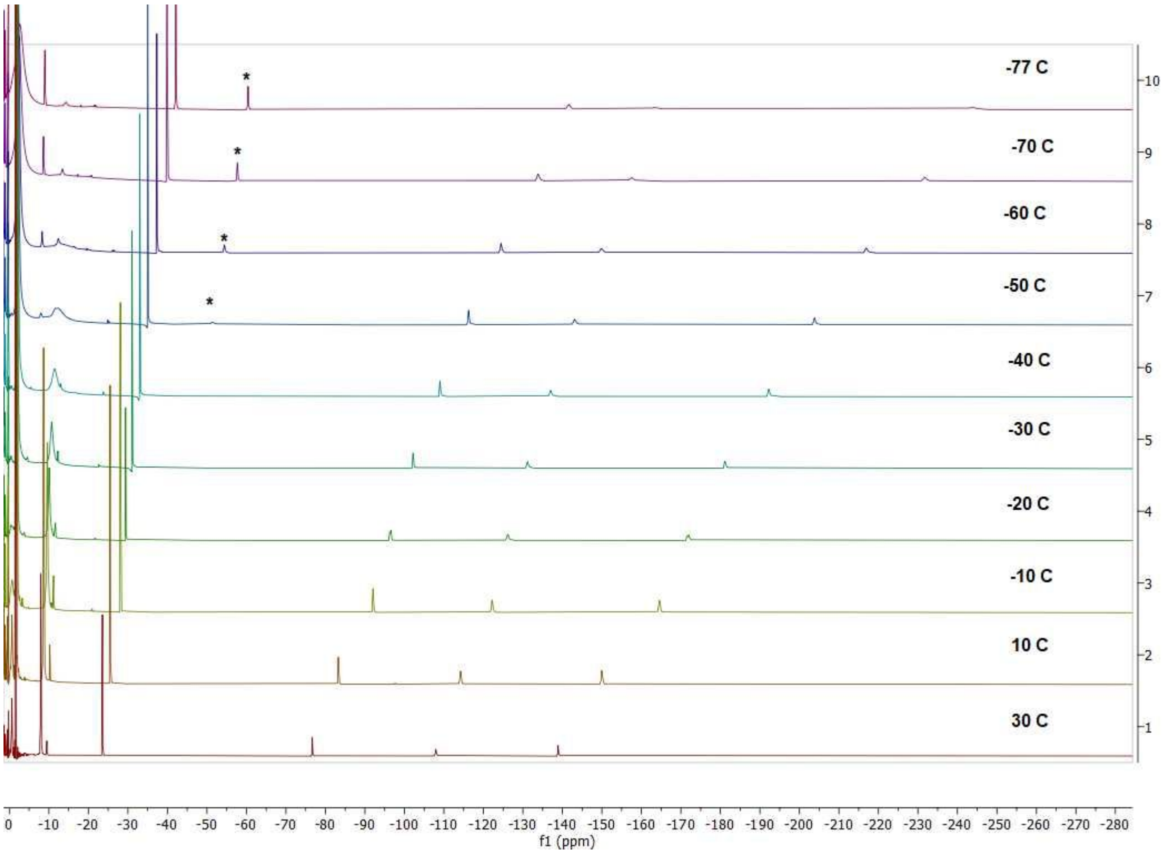

Figure S42. Expanded variable-temperature ^{1}H NMR spectrum of $[Mg_2Cl_3(THF)_6]$[**5**] in THF-D_8. A peak attributable to the minor species is denoted by *.

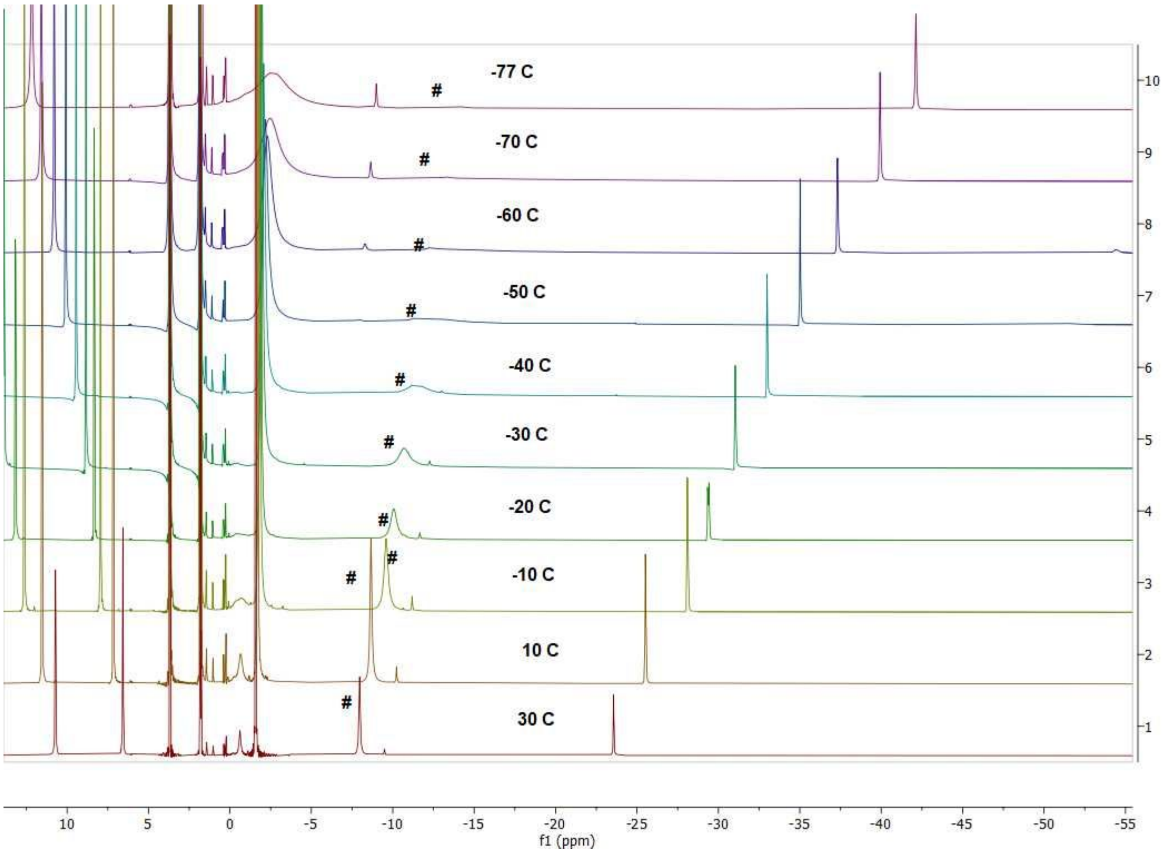

Figure S43. Expanded variable-temperature ^{1}H NMR spectrum of $[Mg_2Cl_3(THF)_6]$[**5**] in THF-D_8. Broadening of the peak starting at –8.09 ppm is denoted by #.

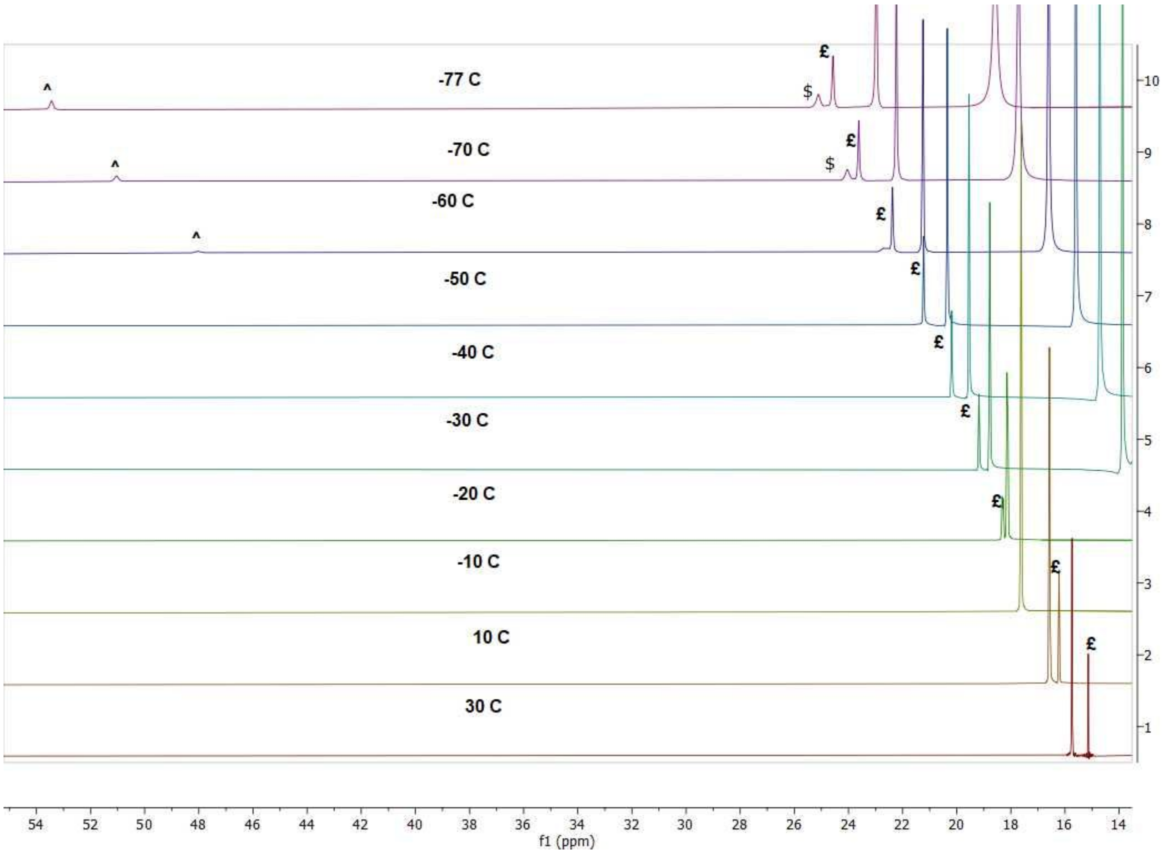

Figure S44. Expanded variable-temperature ^{1}H NMR spectrum of $[Mg_2Cl_3(THF)_6]$[**5**] in THF-D_8. Peaks due to the minor species are denoted with £, $ and ^.

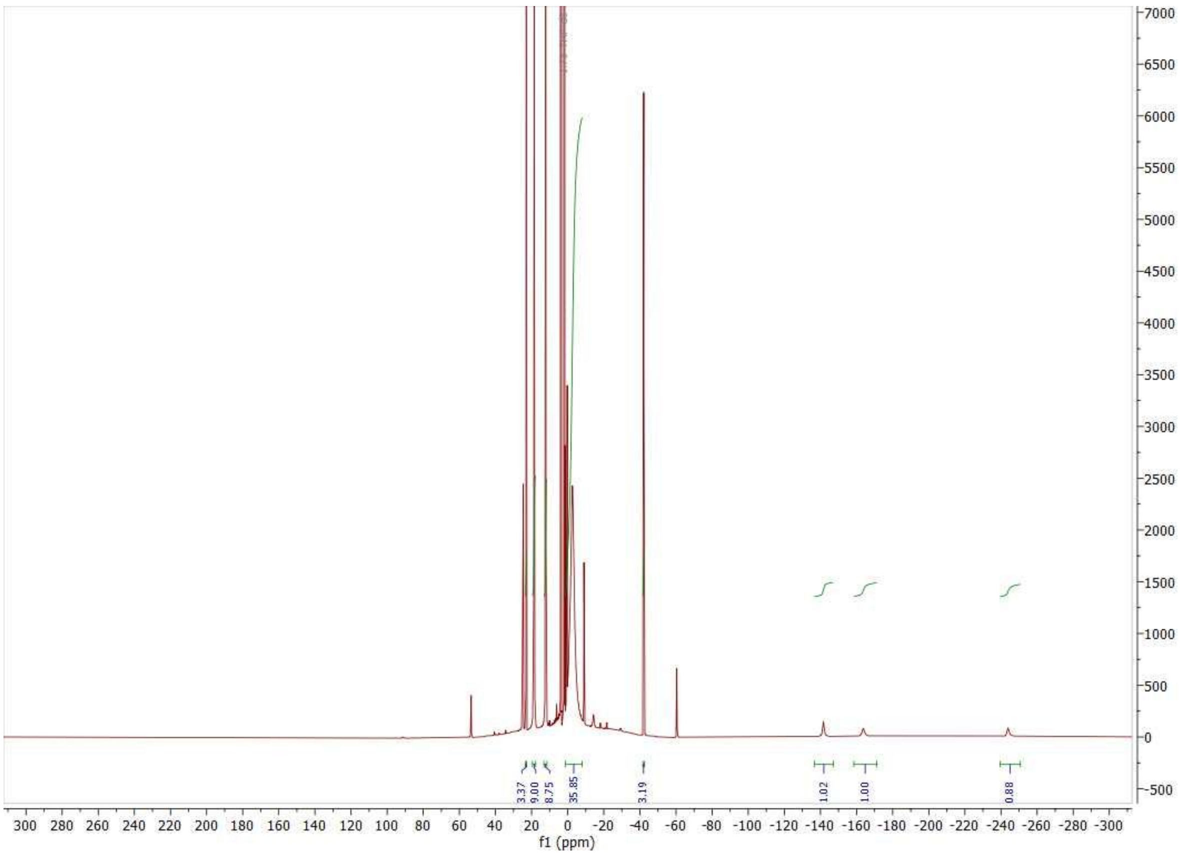

Figure S45. ^{1}H NMR spectrum of $[Mg_2Cl_3(THF)_6]$[**5**] in THF-D_8 at –77 °C with integration of peaks corresponding only to the major species.

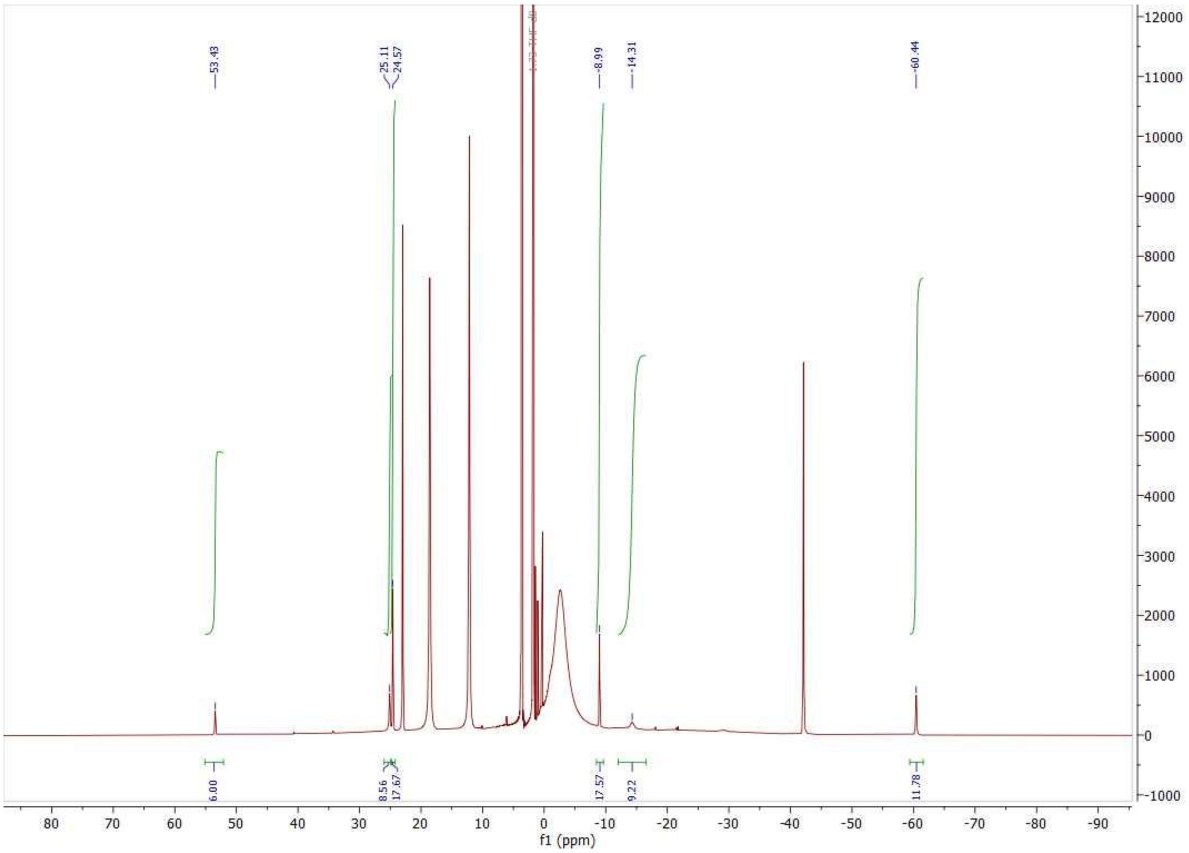

Figure S46. ^{1}H NMR spectrum of $[Mg_2Cl_3(THF)_6][\mathbf{5}]$ in THF-D_8 at –77 °C with integration of peaks corresponding only to the minor species.

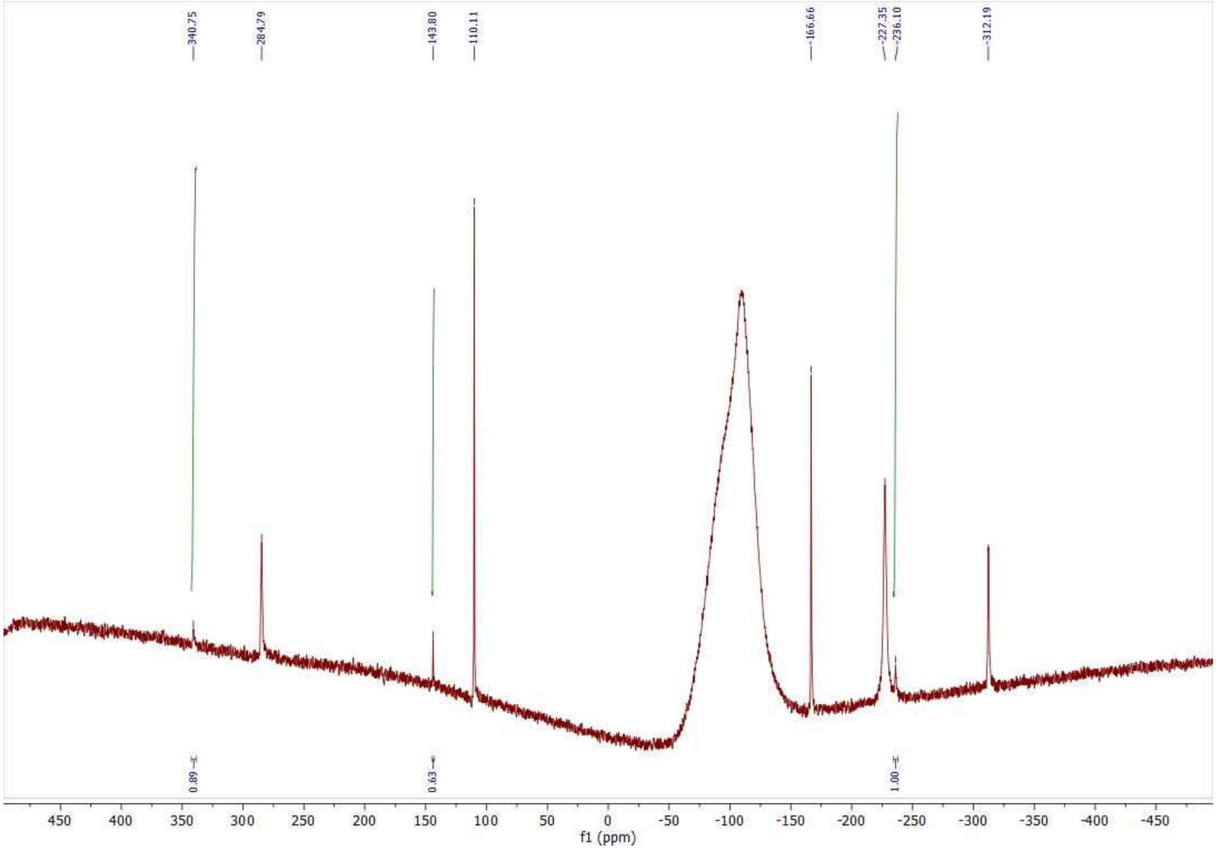

Figure S47. $^{29}Si\{^1H\}$ NMR spectrum of $[Mg_2Cl_3(THF)_6][\mathbf{5}]$ in THF-D_8 at –77 °C. Relative integrals are shown for the minor species.

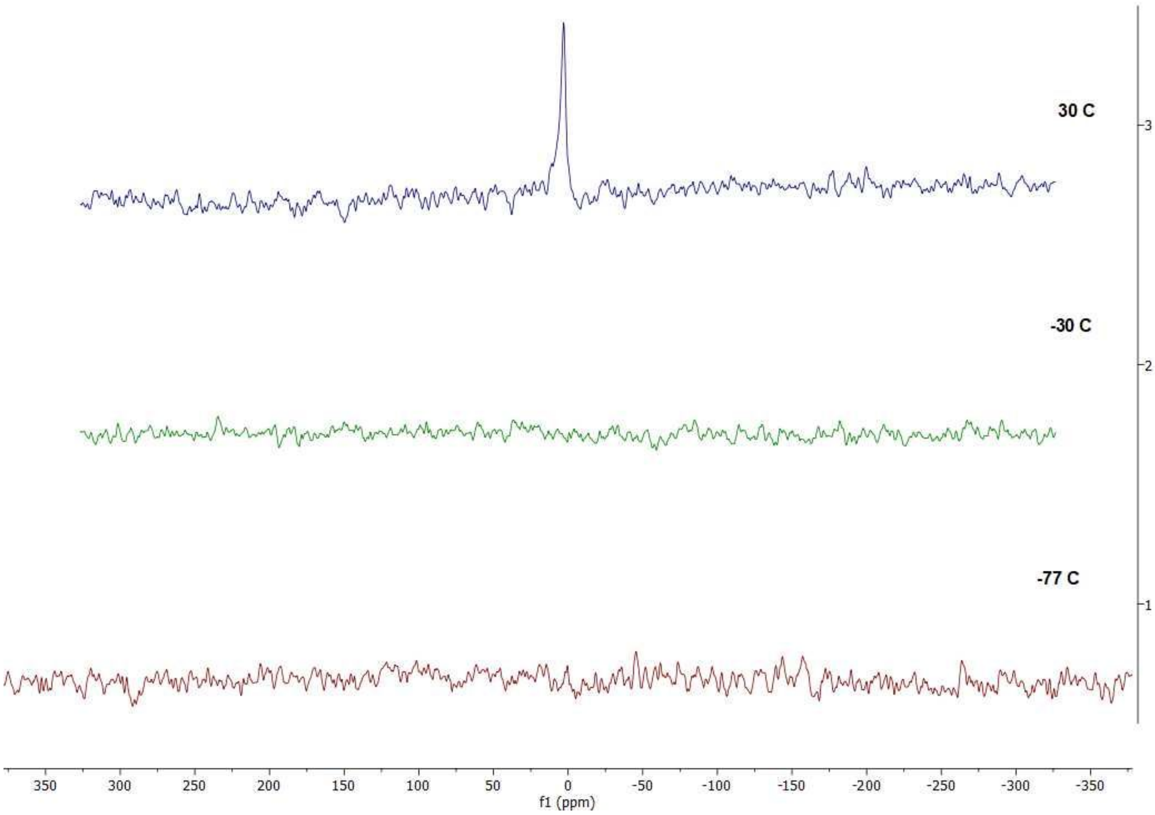

Figure S48. Variable-temperature $^{25}Mg\{^1H\}$ NMR spectrum of $[Mg_2Cl_3(THF)_6][\mathbf{5}]$ in THF-D_8.

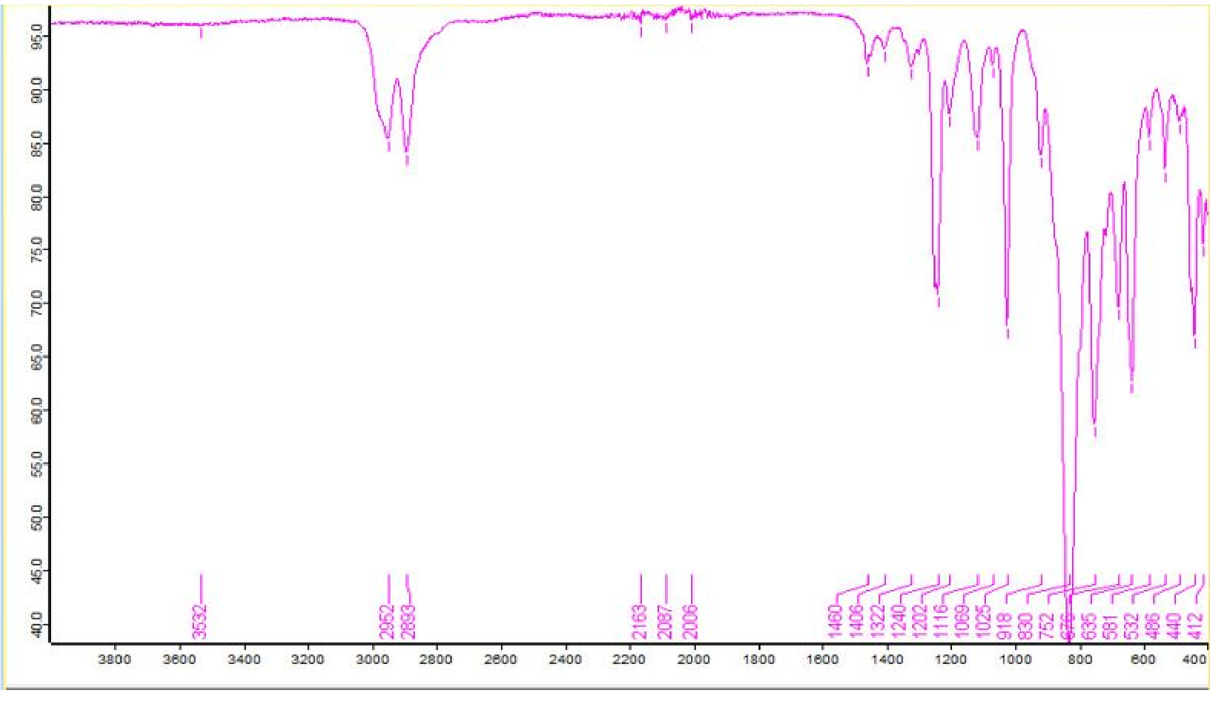

Figure S49. IR spectrum of $[Mg_2Cl_3(THF)_6][\mathbf{5}]$.

X-ray Crystallography

Data on **2** were collected on a Rigaku FR-007HF rotating anode equipped with a Saturn 724+ CCD area detector and a quarter-chi goniometer performing ω scans to fill the Ewald sphere at 100 K, using Cu/Kα radiation. The power of the anode was reduced to 0.7 kW, since at its nominal operating power (1.2 KW) the crystals suffered from extended radiation damage. Data collections for **3**, [$Mg_2Cl_3(THF)_6$][**4**] and [$Mg_2Cl_3(THF)_6$][**5**] were carried out on a Rigaku Gemini Ultra diffractometer equipped with an EOS-CCD detector and a four-circle kappa goniometer performing ω scans to fill the Ewald sphere at 100 K, using Cu/Kα radiation. Crystals were mounted on MiTiGen loops from pump oil kept over activated 4 Å molecular sieves in a glove box. Data collection and processing was handled by CrysAlis Pro. Data for **2** and [$Mg_2Cl_3(THF)_6$][**4**] were collected to 0.83 Å resolution, while for **3** and [$Mg_2Cl_3(THF)_6$][**5**] to 0.82 Å resolution. In the case of **3**, [$Mg_2Cl_3(THF)_6$][**4**] and [$Mg_2Cl_3(THF)_6$][**5**] an analytical absorption correction based on crystal faces (Clark and Reid[4]) was applied. Structure solution and model refinement were performed using the Olex2 package[5] and all software within.

In the case of [$Mg_2Cl_3(THF)_6$][**4**], the model shows significant disorder in both the anionic and cationic part. Specifically, in the [$Mg_2(\mu\text{-}Cl)_3(THF)_6$]$^+$ counter-cation all the chlorides and most of the coordinated THF molecules show disorder between two positions. These disorders were modelled using the PART command and treated with SADI restraints. In the case of the THF molecules, some carbon and oxygen atoms had to be refined isotropically using the ISOR command for the refinement to converge and be stable. Furthermore, the anisotropic displacement parameter of atom C51a had to be constrained to the ADP of its PART 1 partner C51 using the EADP command. The anionic part **4** also features significant disorder with the η^4-Cb'''' ligand, which was modelled as occupying two positions. In order to achieve a stable and converging refinement a RIGU restraint had to be used. All other disorder was treated using SADI and, where necessary, ISOR restraints. An electron density peak of 3.1 e Å^{-3} 1.603 Å away from the thorium atom was observed; this has no chemical meaning and is attributed to absorption effects. The protons of the cyclometallated CH_2 group were found in the electron density map and refined freely.

In the case of [$Mg_2Cl_3(THF)_6$][**5**], the dataset was treated as a two component non-merohedral twin (180˚ rotation along the 0 1 0 direct space axis) with a 90:10 component fraction. Only the major component of the twin was used to generate the hkl4 file, which was then used for the initial solution and refinement. For the final refinement the hkl5 file was used (see Table S1). A common scaling factor was used for both components. Furthermore, a highly disordered half-molecule of THF occupying a special position was identified in the electron density difference map, which could not be appropriately modelled and was excluded using the solvent mask utility in Olex2. Minimal disorder was observed in two of the coordinated THF molecules in the [$Mg_2(\mu\text{-}Cl)_3(THF)_6$]$^+$ cation which was treated using SADI and, where appropriate, ISOR restraints. The final model exhibits an electron density peak of 4.4 e Å^{-3} 1.02 Å from the uranium atom, which has no chemical meaning and is attributed to absorption from the uranium atom and poor separation of the reflections stemming from the two components especially at low resolutions. The cyclometallated carbon C1 was refined isotropically. A model where the dataset was not treated as a twin gave better final R_1 and wR_2 (5.01 and 13.05), but it displayed a somewhat high *b* parameter for the weighting scheme (35.9). The final model displayed also an electron density peak of 2.5 e Å^{-3} 2.724 Å from uranium and 0.88 Å away from the cyclometallated carbon C1 (which has an oblate ellipsoid). Treatment of the dataset as the two-component twin described above resulted in the latter electron density peak disappearing. Furthermore, the *b* factor of the weighting scheme was almost zero.

Table S1. Crystal data and structure refinement.

	**2**	**3**	**4**	**5**
Colour, habit	Yellow, plate	Brown, plate	Yellow, plate	Brown, plate
Size/mm	0.3 × 0.2 × 0.08	0.196 × 0.096 × 0.063	0.16 × 0.12 × 0.04	0.179 × 0.129 × 0.053
Empirical formula	$C_{36}H_{76}Cl_4MgO_5Si_4Th$	$C_{36}H_{76}Cl_4MgO_5Si_4U$	$C_{56}H_{120}Cl_4Mg_2O_6Si_8U$	$C_{56}H_{120}Cl_4Mg_2O_6Si_8U$
FW	1099.48	1105.47	1536.69	1542.68
Crystal system	Monoclinic	Monoclinic	Triclinic	Monoclinic
Space group	$P2_1/c$	$P2_1/c$	*P*–1	$P2_1/c$
a/Å	13.3943(1)	13.3675(2)	12.8836(4)	13.3309(2)
b/Å	15.5141(1)	15.4703(3)	17.0564(6)	17.7676(2)
c/Å	23.9524(2)	23.5661(4)	18.6427(5)	33.4350(5)
α/°	90	90	108.840(3)	90
β/°	91.861(1)	92.7719(17)	90.263(2)	90.6810(10)
γ/°	90	90	91.142(3)	90
V/Å^3	4974.70(4)	4867.75(15)	3876.2(2)	7918.79(19)
Z	4	4	2	4
μ/mm^{-1}	12.966	12.758	9.126	8.631
T/K	100	100	100	100
θ min/max	3.301/68.161	3.756/70.068	3.431/68.251	3.315/70.062
Completeness to θ max (%)	98.7 to 68.161	99.6 to 70.068	99.4 to 68.251	99.7 to 70.062
Reflections Total/ Independent	8983/8764	9215/8526	14120/12331	21288/15631
Parameters	502	502	1161	738
R_{int}	0.0250	0.0462	0.0487	n/a
Final *R*1, *wR*2	0.0239/0.0639	0.0314/0.076	0.0415/0.1093	0.0828/0.2452
Goof	1.028	1.038	1.026	1.014
Largest peak, hole/eÅ^{-3}	1.3/–1.5	1.1/–3.1	3.1/–1.1	4.4/–4.5
ρ_{calc}/g cm^{-3}	1.468	1.508	1.316	1.294
CSD reference	2210714	2210715	2210716	2210717

Computational details

Optimizations of thorium and uranium complexes were carried out by employing the DFT hybrid functional (B3PW91)[6] along with the small core pseudopotential Stuttgart basis set for uranium, thorium, chlorine, and silicon atoms with additional polarization functions for chlorine and silicon atoms.[7] Pople basis sets (6-311+G** basis set for magnesium and 6-31G** for carbon, nitrogen, oxygen, hydrogen atoms) were employed for other atoms.[8] Frequency calculations were performed to locate minima (maxima for transition states) for the optimized structures. All the calculations were performed using Gaussian 09 suite of programs.[9]

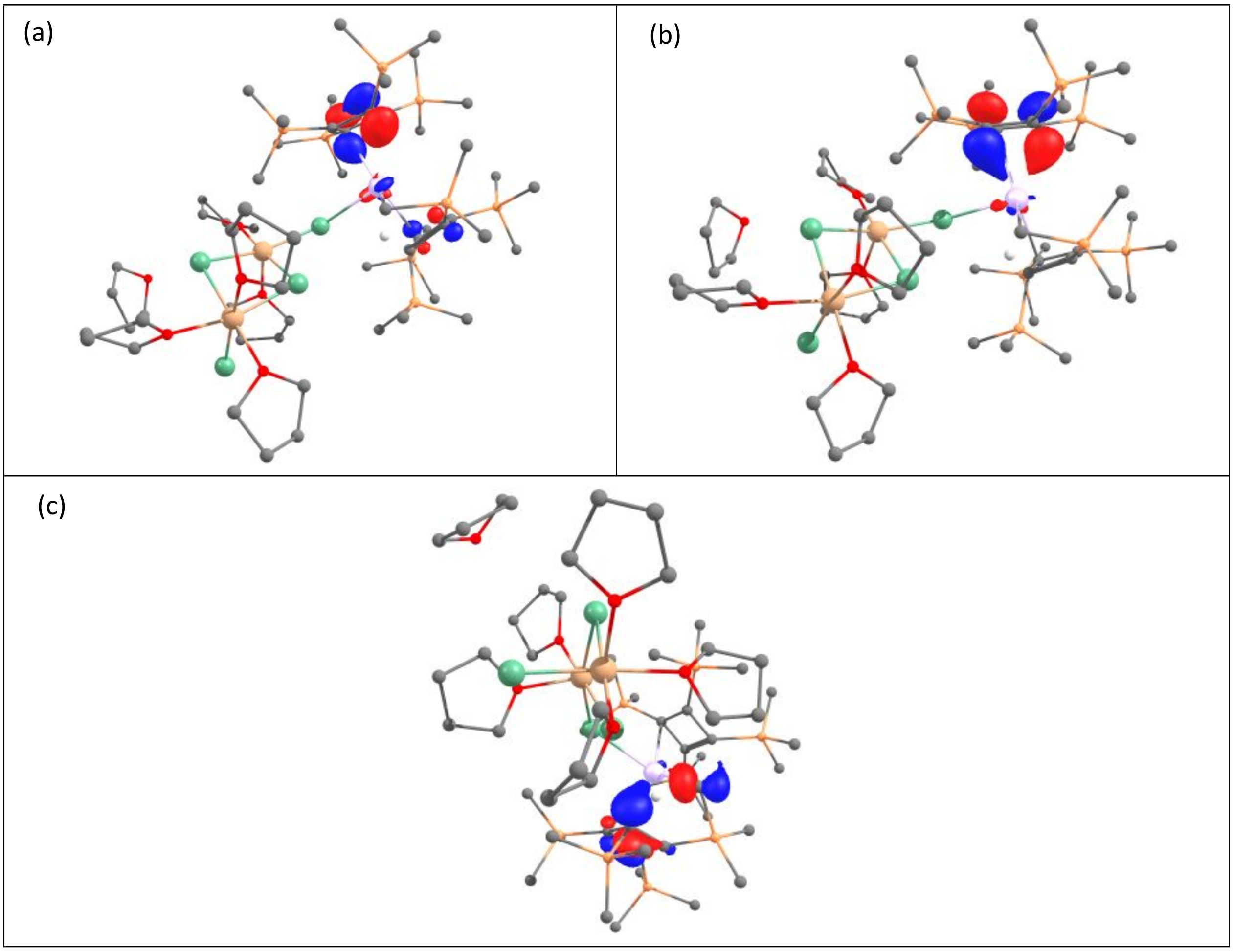

Figure S50. DFT-computed MOs for the thorium transition state: (a) HOMO; (b) HOMO–1; (c) HOMO–2. Isosurface value (0.075). Hydrogen atoms except the atom involved in HAT are excluded for clarity.

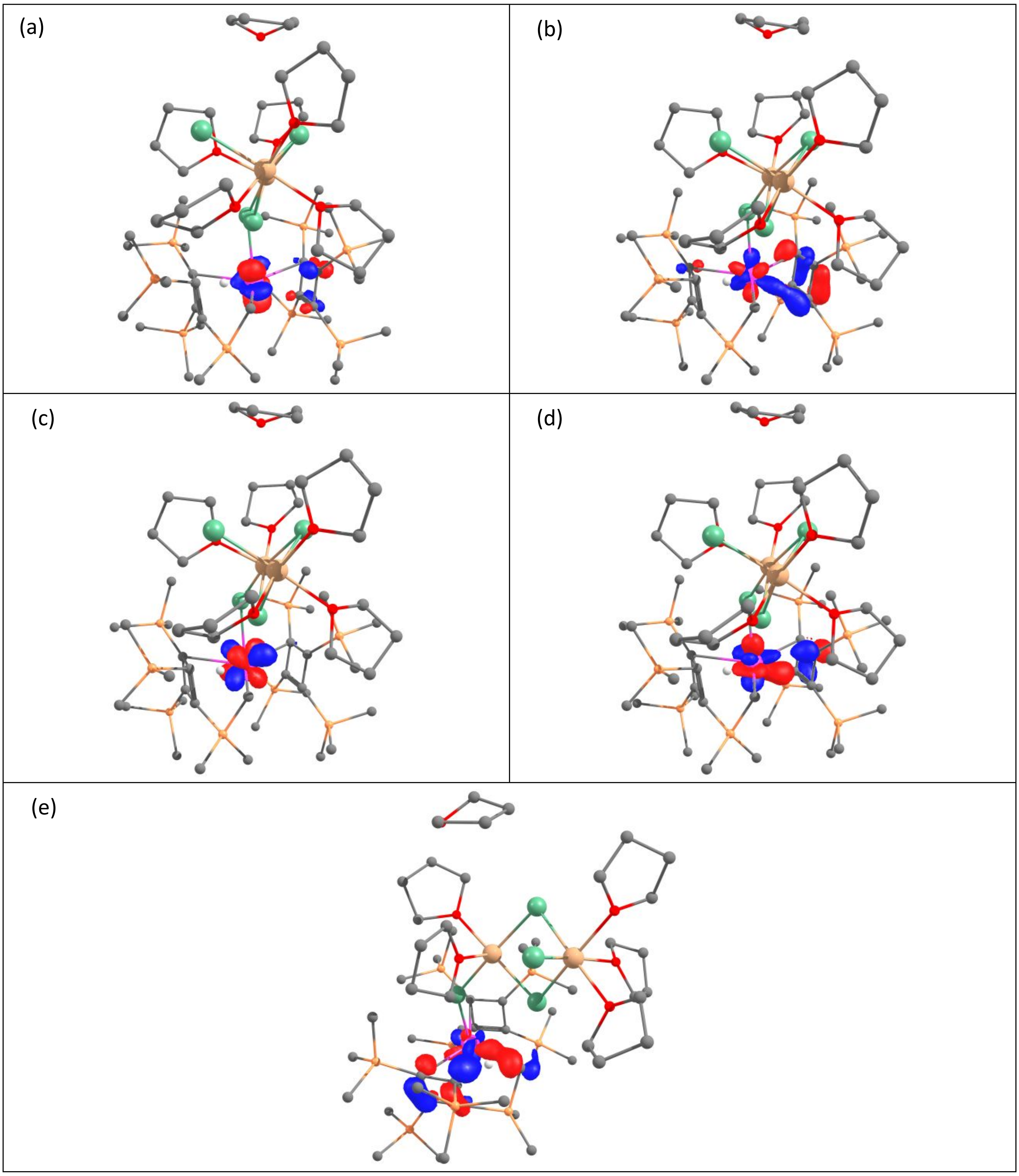

Figure S51. DFT-computed MOs for the uranium transition state: (a) HOMO; (b) HOMO–1; (c) HOMO–2; (d) HOMO–3; (e) HOMO–4. Isosurface value (0.075). Hydrogen atoms except the atom involved in HAT are excluded for clarity.

Table S2. Bonding orbitals from NBO analysis for **2**.

BD (1)Cl 101 -Th 114<br>(85.84%) 0.9265*Cl 101 s(74.16%)p 0.35(25.82%)d 0.00(0.02%)<br>(14.16%) 0.3763*Th 114 s(8.49%)p 2.27(19.30%)d 4.86(41.25%)f 3.65(30.96%)g 0.00(0.01%)
BD (1)Cl 102 -Th 114<br>(85.32%) 0.9237*Cl 102 s(74.47%)p 0.34(25.51%)d 0.00(0.02%)<br>(14.68%) 0.3832*Th 114 s(12.29%)p 1.34(16.44%)d 3.59(44.13%)f 2.21(27.13%)g 0.00(0.01%)
BD (1)Cl 103 -Th 114<br>(86.05%) 0.9276*Cl 103 s(72.66%)p 0.38(27.32%)d 0.00(0.02%)<br>(13.95%) 0.3735*Th 114 s(12.97%)p 1.33(17.32%)d 3.04(39.39%)f 2.34(30.31%)g 0.00(0.01%)
BD (1)Cl 104 -Th 114<br>(84.39%) 0.9187*Cl 104 s(76.50%)p 0.31(23.46%)d 0.00(0.04%)<br>(15.61%) 0.3951*Th 114 s(13.75%)p 1.20(16.51%)d 3.23(44.39%)f 1.84(25.32%)g 0.00(0.02%)

Table S3. Second order perturbation analysis for **2**.

Donor NBO	Acceptor NBO	E(2) kcal/mol
BD (1)Cl 101 -Th 114<br>(85.84%) 0.9265*Cl 101 s(74.16%)p 0.35(25.82%)d 0.00(0.02%)<br>(14.16%) 0.3763*Th 114 s(8.49%)p 2.27(19.30%)d 4.86(41.25%)f 3.65(30.96%)g 0.00(0.01%)	LP*(1)Mg 105<br>s(52.98%)p 0.89(47.01%)d 0.00(0.01%)	65.67
BD (1)Cl 101 -Th 114<br>(85.84%) 0.9265*Cl 101 s(74.16%)p 0.35(25.82%)d 0.00(0.02%)<br>(14.16%) 0.3763*Th 114 s(8.49%)p 2.27(19.30%)d 4.86(41.25%)f 3.65(30.96%)g 0.00(0.01%)	LP*(3)Mg 105<br>s(0.37%)p99.99(98.45%)d 3.16(1.18%)	22.23
BD (1)Cl 102 -Th 114<br>(85.32%) 0.9237*Cl 102 s(74.47%)p 0.34(25.51%)d 0.00(0.02%)<br>(14.68%) 0.3832*Th 114 s(12.29%)p 1.34(16.44%)d 3.59(44.13%) f 2.21(27.13%)g 0.00(0.01%)	LP*(1)Mg 105<br>s(52.98%)p 0.89(47.01%)d 0.00(0.01%)	68.65
BD (1)Cl 102 -Th 114<br>(85.32%) 0.9237*Cl 102 s(74.47%)p 0.34(25.51%)d 0.00(0.02%)<br>(14.68%) 0.3832*Th 114 s(12.29%)p 1.34(16.44%)d 3.59(44.13%) f 2.21(27.13%)g 0.00(0.01%)	LP*(2)Mg 105<br>s(0.11%)p99.99(98.81%)d 9.50(1.08%)	28.77
BD (1)Cl 103 -Th 114<br>(86.05%) 0.9276*Cl 103 s(72.66%)p 0.38(27.32%)d 0.00(0.02%)<br>(13.95%) 0.3735*Th 114 s(12.97%)p 1.33(17.32%)d 3.04(39.39%)f 2.34(30.31%)g 0.00(0.01%)	LP*(1)Mg 105<br>s(52.98%)p 0.89(47.01%)d 0.00(0.01%)	76.70
BD (1)Cl 103 -Th 114<br>(86.05%) 0.9276*Cl 103 s(72.66%)p 0.38(27.32%)d 0.00(0.02%)	LP*(2)Mg 105<br>s(0.11%)p99.99(98.81%)d 9.50(1.08%)	12.85

(13.95%) 0.3735*Th 114 s(12.97%)p 1.33(17.32%)d 3.04(39.39%)f 2.34(30.31%)g 0.00(0.01%)		
BD (1)Cl 103 -Th 114<br>(86.05%) 0.9276*Cl 103 s(72.66%)p 0.38(27.32%)d 0.00(0.02%)<br>(13.95%) 0.3735*Th 114 s(12.97%)p 1.33(17.32%)d 3.04(39.39%)f 2.34(30.31%)g 0.00(0.01%)	LP*(3)Mg 105<br>s(0.37%)p99.99(98.45%)d 3.16(1.18%)	15.28
LP (1)Cl 101<br>s(25.22%)p 2.96(74.76%)d 0.00(0.02%)	LP*(3)Mg 105<br>s(0.37%)p99.99(98.45%)d 3.16(1.18%)	10.11
LP (3)Cl 101<br>s(0.60%)p99.99(99.35%)d 0.07(0.04%)	LP*(3)Mg 105<br>s(0.37%)p99.99(98.45%)d 3.16(1.18%)	9.49
LP (1)Cl 102<br>s(23.87%)p 3.19(76.11%)d 0.00(0.02%)	LP*(2)Mg 105<br>s(0.11%)p99.99(98.81%)d 9.50(1.08%)	10.43
LP (3)Cl 102<br>s(1.47%)p66.79(98.48%)d 0.03(0.04%)	LP*(2)Mg 105<br>s(0.11%)p99.99(98.81%)d 9.50(1.08%)	6.58
LP (1)Cl 103<br>s(26.63%)p 2.75(73.35%)d 0.00(0.01%)	LP*(2)Mg 105<br>s(0.11%)p99.99(98.81%)d 9.50(1.08%)	5.80
LP (1)Cl 103<br>s(26.63%)p 2.75(73.35%)d 0.00(0.01%)	LP*(3)Mg 105<br>s(0.37%)p99.99(98.45%)d 3.16(1.18%)	5.62
LP (3)Cl 103<br>s(0.70%)p99.99(99.26%)d 0.05(0.04%)	LP*(3)Mg 105<br>s(0.37%)p99.99(98.45%)d 3.16(1.18%)	5.11

Table S4. Calculated Wiberg bond index for **2**.

Atom Label	Wiberg bond index	Atom Label	Wiberg bond index
Cl101	0.2559	Cl101	0.8491
Cl102	0.2511	Cl102	0.9254
Cl103	0.2647	Cl103	0.8221
Cl104	0.0055	Cl104	1.2710
Mg105	0.0000	Mg105	0.1511
Th114	0.1511	Th114	0.0000

Table S5. Computed natural charges for **2**.

Atom Label	Natural charges
Cl101	-0.31618
Cl102	-0.27575
Cl103	-0.32831
Cl104	-0.23449
Mg105	1.23916
Th114	0.23655

Table S6. Bonding orbitals from NBO analysis for **3**.

BD (1)Cl 101 - U 114<br>(85.20%) 0.9231*Cl 101 s(63.51%)p 0.57(36.47%)d 0.00(0.02%)<br>(14.80%) 0.3847* U 114 s(9.45%)p 2.19(20.74%)d 4.83(45.66%)f 2.56(24.15%)g 0.00(0.00%)
BD (1)Cl 102 - U 114<br>(84.30%) 0.9182*Cl 102 s(62.75%)p 0.59(37.23%)d 0.00(0.03%)<br>(15.70%) 0.3962* U 114 s(14.28%)p 1.14(16.27%)d 3.48(49.69%)f 1.38(19.75%)g 0.00(0.00%)
BD (1)Cl 103 - U 114<br>(86.22%) 0.9285*Cl 103 s(59.53%)p 0.68(40.45%)d 0.00(0.02%)<br>(13.78%) 0.3713* U 114 s(14.24%)p 1.26(18.01%)d 3.10(44.17%)f 1.66(23.57%)g 0.00(0.00%)
BD (1)Cl 104 - U 114<br>(82.27%) 0.9070*Cl 104 s(59.58%)p 0.68(40.35%)d 0.00(0.07%)<br>(17.73%) 0.4211* U 114 s(14.89%)p 1.10(16.30%)d 2.85(42.38%)f 1.77(26.42%)g 0.00(0.01%)

Table S7. Second order perturbation analysis for **3**.

Donor NBO	Acceptor NBO	E(2) kcal/mol
BD (1)Cl 101 - U 114<br>(85.20%) 0.9231*Cl 101 s(63.51%)p 0.57(36.47%)d 0.00(0.02%)<br>(14.80%) 0.3847* U 114 s(9.45%)p 2.19(20.74%)d 4.83(45.66%)f 2.56(24.15%)g 0.00(0.00%)	LP*(1)Mg 105<br>s(63.24%)p 0.58(36.72%)d 0.00(0.04%)	32.90
BD (1)Cl 101 - U 114<br>(85.20%) 0.9231*Cl 101 s(63.51%)p 0.57(36.47%)d 0.00(0.02%)<br>(14.80%) 0.3847* U 114 s(9.45%)p 2.19(20.74%)d 4.83(45.66%)f 2.56(24.15%)g 0.00(0.00%)	LP*(3)Mg 105<br>s(0.10%)p99.99(98.73%)d12.38(1.18%)	9.39
BD (1)Cl 102 - U 114<br>(84.30%) 0.9182*Cl 102 s(62.75%)p 0.59(37.23%)d 0.00(0.03%)<br>(15.70%) 0.3962* U 114 s(14.28%)p 1.14(16.27%)d 3.48(49.69%)f 1.38(19.75%)g 0.00(0.00%)	LP*(1)Mg 105<br>s(63.24%)p 0.58(36.72%)d 0.00(0.04%)	31.00
BD (1)Cl 102 - U 114<br>(84.30%) 0.9182*Cl 102 s(62.75%)p 0.59(37.23%)d 0.00(0.03%)<br>(15.70%) 0.3962* U 114 s(14.28%)p 1.14(16.27%)d 3.48(49.69%)f 1.38(19.75%)g 0.00(0.00%)	LP*(2)Mg 105<br>s(0.05%)p99.99(98.93%)d20.25(1.01%)	15.61
BD (1)Cl 103 - U 114<br>(86.22%) 0.9285*Cl 103 s(59.53%)p 0.68(40.45%)d 0.00(0.02%)<br>(13.78%) 0.3713* U 114 s(14.24%)p 1.26(18.01%)d 3.10(44.17%)f 1.66(23.57%)g 0.00(0.00%)	LP*(1)Mg 105<br>s(63.24%)p 0.58(36.72%)d 0.00(0.04%)	33.61
LP (3)Cl 101<br>s(2.04%)p47.95(97.92%)d 0.02(0.03%)	LP*(1)Mg 105<br>s(63.24%)p 0.58(36.72%)d 0.00(0.04%)	5.03
LP (3)Cl 101<br>s(2.04%)p47.95(97.92%)d 0.02(0.03%)	LP*(3)Mg 105	8.47

	s(0.10%)p99.99(98.73%)d12.38(1.18%)	
LP (1)Cl 102<br>s(36.05%)p 1.77(63.94%)d 0.00(0.01%)	LP*(2)Mg 105<br>s(0.05%)p99.99(98.93%)d20.25(1.01%)	5.88
LP (3)Cl 102<br>s(1.05%)p94.19(98.91%)d 0.04(0.04%)	LP*(2)Mg 105<br>s(0.05%)p99.99(98.93%)d20.25(1.01%)	8.06
LP (3)Cl 103<br>s(4.82%)p19.73(95.15%)d 0.01(0.03%)	LP*(1)Mg 105<br>s(63.24%)p 0.58(36.72%)d 0.00(0.04%)	5.45
LP (3)Cl 103<br>s(4.82%)p19.73(95.15%)d 0.01(0.03%)	LP*(2)Mg 105<br>s(0.05%)p99.99(98.93%)d20.25(1.01%)	5.48
LP (3)Cl 103<br>s(4.82%)p19.73(95.15%)d 0.01(0.03%)	LP*(3)Mg 105<br>s(0.10%)p99.99(98.73%)d12.38(1.18%)	6.08

Table S8. Calculated Wiberg bond index for **3**.

Atom Label	Wiberg bond index	Atom Label	Wiberg bond index
Cl101	0.2676	Cl101	0.7224
Cl102	0.2589	Cl102	0.8184
Cl103	0.2844	Cl103	0.6738
Cl104	0.0052	Cl104	1.2444
Mg105	0.0000	Mg105	0.1230
U114	0.1230	U114	0.0000

Table S9. Computed Natural charges for **3**.

Atom Label	Natural charges
Cl101	-0.37556
Cl102	-0.32451
Cl103	-0.40625
Cl104	-0.23369
Mg105	1.21920
U114	0.30411

Table S10. Bonding orbitals from NBO analysis for **4**.

BD (1) C 51 -Th 197<br>(83.75%) 0.9152* C 51 s(30.08%)p 2.32(69.92%)d 0.00(0.01%)<br>(16.25%) 0.4031*Th 197 s(12.41%)p 0.06(0.75%)d 6.19(76.80%)f 0.81(10.03%)g 0.00(0.02%)
BD (1)Cl 180 -Th 197<br>(84.88%) 0.9213*Cl 180 s(79.35%)p 0.26(20.61%)d 0.00(0.04%)<br>(15.12%) 0.3889*Th 197 s(12.98%)p 0.92(11.88%)d 4.12(53.48%)f 1.67(21.65%)g 0.00(0.02%)

Table S11. Second order perturbation analysis for **4**.

Donor NBO	Acceptor NBO	E(2) kcal/mol
BD (1) C 28 - C 33<br>(50.13%) 0.7080* C 28 s(24.64%)p 3.06(75.31%)d 0.00(0.05%)<br>(49.87%) 0.7062* C 33 s(25.98%)p 2.85(73.97%)d 0.00(0.05%)	LP*(4)Th 197<br>s(25.07%)p 0.16(3.91%)d 2.20(55.13%) f 0.63(15.88%)g 0.00(0.01%)	31.93
BD (1) C 28 - C 33<br>(50.13%) 0.7080* C 28 s(24.64%)p 3.06(75.31%)d 0.00(0.05%)<br>(49.87%) 0.7062* C 33 s(25.98%)p 2.85(73.97%)d 0.00(0.05%)	LP*(13)Th 197<br>s(1.81%)p16.45(29.77%)d 3.22(5.82%) f34.60(62.60%)g 0.00(0.01%)	7.88
BD (1) C 28 -Si 189<br>(76.42%) 0.8742* C 28 s(39.05%)p 1.56(60.92%)d 0.00(0.02%)<br>(23.58%) 0.4855*Si 189 s(27.54%)p 2.59(71.40%)d 0.04(1.06%)	LP*(2)Th 197<br>s(0.29%)p 5.87(1.68%)d99.99(89.30%)f30.53(8.73%)g 0.02(0.01%)	13.83
BD (1) C 28 -Si 189<br>(76.42%) 0.8742* C 28 s(39.05%)p 1.56(60.92%)d 0.00(0.02%)<br>(23.58%) 0.4855*Si 189 s(27.54%)p 2.59(71.40%)d 0.04(1.06%)	LP*(13)Th 197<br>s(1.81%)p16.45(29.77%)d 3.22(5.82%) f34.60(62.60%)g 0.00(0.01%)	17.63
BD (1) C 32 - C 39<br>(49.77%) 0.7055* C 32 s(25.04%)p 2.99(74.91%)d 0.00(0.05%)<br>(50.23%) 0.7087* C 39 s(28.00%)p 2.57(71.95%)d 0.00(0.05%)	LP*(4)Th 197<br>s(25.07%)p 0.16(3.91%)d 2.20(55.13%) f 0.63(15.88%)g 0.00(0.01%)	20.46
BD (1) C 32 - C 39<br>(49.77%) 0.7055* C 32 s(25.04%)p 2.99(74.91%)d 0.00(0.05%)<br>(50.23%) 0.7087* C 39 s(28.00%)p 2.57(71.95%)d 0.00(0.05%)	LP*(8)Th 197<br>s(5.00%)p 8.78(43.94%)d 1.71(8.53%) f 8.50(42.51%)g 0.00(0.01%)	19.97
BD (1) C 32 -Si 190<br>(76.63%) 0.8754* C 32 s(40.10%)p 1.49(59.87%)d 0.00(0.02%)<br>(23.37%) 0.4835*Si 190 s(27.09%)p 2.65(71.81%)d 0.04(1.10%)	LP*(5)Th 197<br>s(5.05%)p 1.01(5.11%)d 0.92(4.64%) f16.86(85.19%)g 0.00(0.00%)	20.47
BD (1) C 33 - C 39<br>(50.23%) 0.7087* C 33 s(27.81%)p 2.59(72.14%)d 0.00(0.05%)<br>(49.77%) 0.7055* C 39 s(27.42%)p 2.64(72.52%)d 0.00(0.05%)	LP*(4)Th 197<br>s(25.07%)p 0.16(3.91%)d 2.20(55.13%) f 0.63(15.88%)g 0.00(0.01%)	26.25

BD (1) C 33 - C 39<br>(50.23%) 0.7087* C 33 s(27.81%)p 2.59(72.14%)d 0.00(0.05%)<br>(49.77%) 0.7055* C 39 s(27.42%)p 2.64(72.52%)d 0.00(0.05%)	LP*(8)Th 197<br>s(5.00%)p 8.78(43.94%)d 1.71(8.53%) f 8.50(42.51%)g 0.00(0.01%)	18.18
BD (1) C 33 -Si 192<br>(76.83%) 0.8765* C 33 s(40.22%)p 1.49(59.76%)d 0.00(0.02%)<br>(23.17%) 0.4813*Si 192 s(26.07%)p 2.79(72.78%)d 0.04(1.15%)	LP*(4)Th 197<br>s(25.07%)p 0.16(3.91%)d 2.20(55.13%) f 0.63(15.88%)g 0.00(0.01%)	7.68
BD (1) C 33 -Si 192<br>(76.83%) 0.8765* C 33 s(40.22%)p 1.49(59.76%)d 0.00(0.02%)<br>(23.17%) 0.4813*Si 192 s(26.07%)p 2.79(72.78%)d 0.04(1.15%)	LP*(6)Th 197<br>s(8.35%)p 2.15(17.96%)d 1.02(8.50%) f 7.80(65.17%)g 0.00(0.01%)	30.54
BD (1) C 39 -Si 194<br>(76.51%) 0.8747* C 39 s(39.42%)p 1.54(60.55%)d 0.00(0.02%)<br>(23.49%) 0.4846*Si 194 s(25.86%)p 2.82(72.98%)d 0.04(1.16%)	LP*(8)Th 197<br>s(5.00%)p 8.78(43.94%)d 1.71(8.53%) f 8.50(42.51%)g 0.00(0.01%)	19.08
BD (1) C 39 -Si 194<br>(76.51%) 0.8747* C 39 s(39.42%)p 1.54(60.55%)d 0.00(0.02%)<br>(23.49%) 0.4846*Si 194 s(25.86%)p 2.82(72.98%)d 0.04(1.16%)	LP*(10)Th 197<br>s(0.04%)p99.99(38.89%)d99.99(6.45%)f99.99(54.58%)g 1.11(0.04%)	7.66
LP (1) C 28<br>s(12.73%)p 6.86(87.26%)d 0.00(0.02%)	LP*(2)Th 197<br>s(0.29%)p 5.87(1.68%)d99.99(89.30%)f30.53(8.73%)g 0.02(0.01%)	73.32
LP (1) C 28<br>s(12.73%)p 6.86(87.26%)d 0.00(0.02%)	LP*(4)Th 197<br>s(25.07%)p 0.16(3.91%)d 2.20(55.13%) f 0.63(15.88%)g 0.00(0.01%)	16.45
LP (1) C 32<br>s(10.79%)p 8.26(89.19%)d 0.00(0.02%)	LP*(1)Th 197<br>s(0.07%)p18.27(1.37%)d99.99(90.54%)f99.99(8.01%)g 0.11(0.01%)	35.54
LP (1) C 32<br>s(10.79%)p 8.26(89.19%)d 0.00(0.02%)	LP*(5)Th 197<br>s(5.05%)p 1.01(5.11%)d 0.92(4.64%) f16.86(85.19%)g 0.00(0.00%)	14.23
LP (1) C 33<br>s(6.05%)p15.52(93.92%)d 0.00(0.03%)	LP*(1)Th 197<br>s(0.07%)p18.27(1.37%)d99.99(90.54%)f99.99(8.01%)g 0.11(0.01%)	31.63
LP (1) C 33<br>s(6.05%)p15.52(93.92%)d 0.00(0.03%)	LP*(4)Th 197<br>s(25.07%)p 0.16(3.91%)d 2.20(55.13%) f 0.63(15.88%)g 0.00(0.01%)	18.36
LP (1) C 33<br>s(6.05%)p15.52(93.92%)d 0.00(0.03%)	LP*(6)Th 197<br>s(8.35%)p 2.15(17.96%)d 1.02(8.50%) f 7.80(65.17%)g 0.00(0.01%)	9.54
BD (1) C 35 - C 40<br>(51.37%) 0.7167* C 35 s(24.48%)p 3.08(75.47%)d 0.00(0.04%)<br>(48.63%) 0.6974* C 40 s(25.23%)p 2.96(74.72%)d 0.00(0.05%)	LP*(2)Th 197<br>s(0.29%)p 5.87(1.68%)d99.99(89.30%)f30.53(8.73%)g 0.02(0.01%)	12.20
BD (1) C 35 - C 40	LP*(3)Th 197	27.62

(51.37%) 0.7167* C 35 s(24.48%)p 3.08(75.47%)d 0.00(0.04%)<br>(48.63%) 0.6974* C 40 s(25.23%)p 2.96(74.72%)d 0.00(0.05%)	s(0.48%)p 8.94(4.28%)d99.99(68.16%)f56.60(27.07%)g 0.03(0.01%)	
BD (1) C 35 - C 40<br>(51.37%) 0.7167* C 35 s(24.48%)p 3.08(75.47%)d 0.00(0.04%)<br>(48.63%) 0.6974* C 40 s(25.23%)p 2.96(74.72%)d 0.00(0.05%)	LP*(4)Th 197<br>s(25.07%)p 0.16(3.91%)d 2.20(55.13%) f 0.63(15.88%)g 0.00(0.01%)	8.80
BD (1) C 35 - C 40<br>(51.37%) 0.7167* C 35 s(24.48%)p 3.08(75.47%)d 0.00(0.04%)<br>(48.63%) 0.6974* C 40 s(25.23%)p 2.96(74.72%)d 0.00(0.05%)	LP*(6)Th 197<br>s(8.35%)p 2.15(17.96%)d 1.02(8.50%) f 7.80(65.17%)g 0.00(0.01%)	7.81
BD (1) C 35 - C 40<br>(51.37%) 0.7167* C 35 s(24.48%)p 3.08(75.47%)d 0.00(0.04%)<br>(48.63%) 0.6974* C 40 s(25.23%)p 2.96(74.72%)d 0.00(0.05%)	LP*(9)Th 197<br>s(4.01%)p15.49(62.08%)d 1.68(6.74%) f 6.78(27.16%)g 0.00(0.01%)	18.88
BD (1) C 35 - C 47<br>(51.99%) 0.7210* C 35 s(24.08%)p 3.15(75.87%)d 0.00(0.05%)<br>(48.01%) 0.6929* C 47 s(23.34%)p 3.28(76.60%)d 0.00(0.07%)	LP*(1)Th 197<br>s(0.07%)p18.27(1.37%)d99.99(90.54%)f99.99(8.01%)g 0.11(0.01%)	12.77
BD (1) C 35 - C 47<br>(51.99%) 0.7210* C 35 s(24.08%)p 3.15(75.87%)d 0.00(0.05%)<br>(48.01%) 0.6929* C 47 s(23.34%)p 3.28(76.60%)d 0.00(0.07%)	LP*(9)Th 197<br>s(4.01%)p15.49(62.08%)d 1.68(6.74%) f 6.78(27.16%)g 0.00(0.01%)	24.94
BD (1) C 35 - C 47<br>(51.99%) 0.7210* C 35 s(24.08%)p 3.15(75.87%)d 0.00(0.05%)<br>(48.01%) 0.6929* C 47 s(23.34%)p 3.28(76.60%)d 0.00(0.07%)	LP*(12)Th 197<br>s(11.28%)p 1.73(19.55%)d 0.90(10.17%)f 5.23(58.99%)g 0.00(0.01%)	8.61
BD (1) C 35 - H 124<br>(63.78%) 0.7986* C 35 s(26.58%)p 2.76(73.37%)d 0.00(0.05%)<br>(36.22%) 0.6018* H 124 s(99.95%)p 0.00(0.05%)	LP*(9)Th 197<br>s(4.01%)p15.49(62.08%)d 1.68(6.74%) f 6.78(27.16%)g 0.00(0.01%)	20.92
BD (1) C 35 -Si 191<br>(79.02%) 0.8889* C 35 s(24.84%)p 3.02(75.14%)d 0.00(0.02%)<br>(20.98%) 0.4581*Si 191 s(25.99%)p 2.81(72.99%)d 0.04(1.02%)	LP*(9)Th 197<br>s(4.01%)p15.49(62.08%)d 1.68(6.74%) f 6.78(27.16%)g 0.00(0.01%)	7.41
BD (1) C 40 - C 49<br>(49.88%) 0.7063* C 40 s(25.59%)p 2.91(74.36%)d 0.00(0.05%)<br>(50.12%) 0.7079* C 49 s(29.63%)p 2.37(70.32%)d 0.00(0.05%)	LP*(3)Th 197<br>s(0.48%)p 8.94(4.28%)d99.99(68.16%)f56.60(27.07%)g 0.03(0.01%)	8.80
BD (1) C 40 - C 49<br>(49.88%) 0.7063* C 40 s(25.59%)p 2.91(74.36%)d 0.00(0.05%)	LP*(5)Th 197<br>s(5.05%)p 1.01(5.11%)d 0.92(4.64%) f16.86(85.19%)g 0.00(0.00%)	16.50

(50.12%) 0.7079* C 49 s(29.63%)p 2.37(70.32%)d 0.00(0.05%)		
BD (1) C 40 - C 49<br>(49.88%) 0.7063* C 40 s(25.59%)p 2.91(74.36%)d 0.00(0.05%)<br>(50.12%) 0.7079* C 49 s(29.63%)p 2.37(70.32%)d 0.00(0.05%)	LP*(9)Th 197<br>s(4.01%)p15.49(62.08%)d 1.68(6.74%) f 6.78(27.16%)g 0.00(0.01%)	8.81
BD (1) C 40 -Si 193<br>(76.67%) 0.8756* C 40 s(42.32%)p 1.36(57.65%)d 0.00(0.03%)<br>(23.33%) 0.4830*Si 193 s(26.80%)p 2.69(72.12%)d 0.04(1.08%)	LP*(3)Th 197<br>s(0.48%)p 8.94(4.28%)d99.99(68.16%)f56.60(27.07%)g 0.03(0.01%)	15.25
BD (1) C 40 -Si 193<br>(76.67%) 0.8756* C 40 s(42.32%)p 1.36(57.65%)d 0.00(0.03%)<br>(23.33%) 0.4830*Si 193 s(26.80%)p 2.69(72.12%)d 0.04(1.08%)	LP*(7)Th 197<br>s(7.92%)p 0.08(0.64%)d 0.63(4.97%) f10.92(86.45%)g 0.00(0.02%)	7.52
BD (1) C 47 - C 49<br>(49.84%) 0.7060* C 47 s(29.48%)p 2.39(70.46%)d 0.00(0.06%)<br>(50.16%) 0.7082* C 49 s(29.34%)p 2.41(70.60%)d 0.00(0.05%)	LP*(5)Th 197<br>s(5.05%)p 1.01(5.11%)d 0.92(4.64%) f16.86(85.19%)g 0.00(0.00%)	21.66
BD (1) C 47 - C 49<br>(49.84%) 0.7060* C 47 s(29.48%)p 2.39(70.46%)d 0.00(0.06%)<br>(50.16%) 0.7082* C 49 s(29.34%)p 2.41(70.60%)d 0.00(0.05%)	LP*(7)Th 197<br>s(7.92%)p 0.08(0.64%)d 0.63(4.97%) f10.92(86.45%)g 0.00(0.02%)	9.16
BD (1) C 47 - C 49<br>(49.84%) 0.7060* C 47 s(29.48%)p 2.39(70.46%)d 0.00(0.06%)<br>(50.16%) 0.7082* C 49 s(29.34%)p 2.41(70.60%)d 0.00(0.05%)	LP*(9)Th 197<br>s(4.01%)p15.49(62.08%)d 1.68(6.74%) f 6.78(27.16%)g 0.00(0.01%)	9.97
BD (2) C 47 - C 49<br>(56.25%) 0.7500* C 47 s(0.25%)p99.99(99.67%)d 0.33(0.08%)<br>(43.75%) 0.6614* C 49 s(0.68%)p99.99(99.21%)d 0.15(0.10%)	LP*(1)Th 197<br>s(0.07%)p18.27(1.37%)d99.99(90.54%)f99.99(8.01%)g 0.11(0.01%)	8.27
BD (2) C 47 - C 49<br>(56.25%) 0.7500* C 47 s(0.25%)p99.99(99.67%)d 0.33(0.08%)<br>(43.75%) 0.6614* C 49 s(0.68%)p99.99(99.21%)d 0.15(0.10%)	LP*(4)Th 197<br>s(25.07%)p 0.16(3.91%)d 2.20(55.13%) f 0.63(15.88%)g 0.00(0.01%)	8.05
BD (1) C 47 -Si 195<br>(78.62%) 0.8867* C 47 s(47.02%)p 1.13(52.96%)d 0.00(0.02%)<br>(21.38%) 0.4624*Si 195 s(23.23%)p 3.25(75.38%)d 0.06(1.39%)	LP*(1)Th 197<br>s(0.07%)p18.27(1.37%)d99.99(90.54%)f99.99(8.01%)g 0.11(0.01%)	26.75
BD (1) C 47 -Si 195<br>(78.62%) 0.8867* C 47 s(47.02%)p 1.13(52.96%)d 0.00(0.02%)<br>(21.38%) 0.4624*Si 195 s(23.23%)p 3.25(75.38%)d 0.06(1.39%)	LP*(3)Th 197<br>s(0.48%)p 8.94(4.28%)d99.99(68.16%)f56.60(27.07%)g 0.03(0.01%)	8.09
BD (1) C 47 -Si 195	LP*(4)Th 197	9.64

(78.62%) 0.8867* C 47 s(47.02%)p 1.13(52.96%)d 0.00(0.02%)<br>(21.38%) 0.4624*Si 195 s(23.23%)p 3.25(75.38%)d 0.06(1.39%)	s(25.07%)p 0.16(3.91%)d 2.20(55.13%)<br>f 0.63(15.88%)g 0.00(0.01%)	
BD (1) C 47 -Si 195<br>(78.62%) 0.8867* C 47 s(47.02%)p 1.13(52.96%)d 0.00(0.02%)<br>(21.38%) 0.4624*Si 195 s(23.23%)p 3.25(75.38%)d 0.06(1.39%)	LP*(5)Th 197<br>s(5.05%)p 1.01(5.11%)d 0.92(4.64%)<br>f16.86(85.19%)g 0.00(0.00%)	16.62
BD (1) C 47 -Si 195<br>(78.62%) 0.8867* C 47 s(47.02%)p 1.13(52.96%)d 0.00(0.02%)<br>(21.38%) 0.4624*Si 195 s(23.23%)p 3.25(75.38%)d 0.06(1.39%)	LP*(6)Th 197<br>s(8.35%)p 2.15(17.96%)d 1.02(8.50%)<br>f 7.80(65.17%)g 0.00(0.01%)	23.71
BD (1) C 47 -Si 195<br>(78.62%) 0.8867* C 47 s(47.02%)p 1.13(52.96%)d 0.00(0.02%)<br>(21.38%) 0.4624*Si 195 s(23.23%)p 3.25(75.38%)d 0.06(1.39%)	LP*(8)Th 197<br>s(5.00%)p 8.78(43.94%)d 1.71(8.53%)<br>f 8.50(42.51%)g 0.00(0.01%)	15.12
BD (1) C 47 -Si 195<br>(78.62%) 0.8867* C 47 s(47.02%)p 1.13(52.96%)d 0.00(0.02%)<br>(21.38%) 0.4624*Si 195 s(23.23%)p 3.25(75.38%)d 0.06(1.39%)	LP*(9)Th 197<br>s(4.01%)p15.49(62.08%)d 1.68(6.74%)<br>f 6.78(27.16%)g 0.00(0.01%)	13.44
BD (1) C 49 -Si 196<br>(76.86%) 0.8767* C 49 s(40.17%)p 1.49(59.80%)d 0.00(0.02%)<br>(23.14%) 0.4810*Si 196 s(24.97%)p 2.96(73.84%)d 0.05(1.18%)	LP*(7)Th 197<br>s(7.92%)p 0.08(0.64%)d 0.63(4.97%)<br>f10.92(86.45%)g 0.00(0.02%)	8.41
BD (1) C 49 -Si 196<br>(76.86%) 0.8767* C 49 s(40.17%)p 1.49(59.80%)d 0.00(0.02%)<br>(23.14%) 0.4810*Si 196 s(24.97%)p 2.96(73.84%)d 0.05(1.18%)	LP*(8)Th 197<br>s(5.00%)p 8.78(43.94%)d 1.71(8.53%)<br>f 8.50(42.51%)g 0.00(0.01%)	15.22
BD (1) C 51 - H 157<br>(63.36%) 0.7960* C 51 s(23.66%)p 3.22(76.30%)d 0.00(0.04%)<br>(36.64%) 0.6053* H 157 s(99.96%)p 0.00(0.04%)	LP*(7)Th 197<br>s(7.92%)p 0.08(0.64%)d 0.63(4.97%)<br>f10.92(86.45%)g 0.00(0.02%)	9.58
BD (1) C 51 - H 157<br>(63.36%) 0.7960* C 51 s(23.66%)p 3.22(76.30%)d 0.00(0.04%)<br>(36.64%) 0.6053* H 157 s(99.96%)p 0.00(0.04%)	LP*(13)Th 197<br>s(1.81%)p16.45(29.77%)d 3.22(5.82%)<br>f34.60(62.60%)g 0.00(0.01%)	10.03
BD (1) C 51 - H 167<br>(62.76%) 0.7922* C 51 s(21.45%)p 3.66(78.50%)d 0.00(0.05%<br>(37.24%) 0.6103* H 167 s(99.96%)p 0.00(0.04%)	LP*(7)Th 197<br>s(7.92%)p 0.08(0.64%)d 0.63(4.97%)<br>f10.92(86.45%)g 0.00(0.02%)	20.61
BD (1) C 51 - H 167<br>(62.76%) 0.7922* C 51 s(21.45%)p 3.66(78.50%)d 0.00(0.05%	LP*(11)Th 197<br>s(0.41%)p79.46(32.58%)d16.43(6.74%) f99.99(60.26%)g 0.05(0.02%)	10.09

(37.24%) 0.6103* H 167 s(99.96%)p 0.00(0.04%)		
BD (1) C 51 -Si 195<br>(74.62%) 0.8638* C 51 s(24.68%)p 3.05(75.28%)d 0.00(0.04%)<br>(25.38%) 0.5038*Si 195 s(29.58%)p 2.35(69.44%)d 0.03(0.98%)	LP*(7)Th 197<br>s(7.92%)p 0.08(0.64%)d 0.63(4.97%) f10.92(86.45%)g 0.00(0.02%)	32.63
LP (1) C 40<br>s(6.91%)p13.48(93.07%)d 0.00(0.02%)	LP*(1)Th 197<br>s(0.07%)p18.27(1.37%)d99.99(90.54%)f99.99(8.01%)g 0.11(0.01%)	10.96
LP (1) C 40<br>s(6.91%)p13.48(93.07%)d 0.00(0.02%)	LP*(2)Th 197<br>s(0.29%)p 5.87(1.68%)d99.99(89.30%)f30.53(8.73%)g 0.02(0.01%)	23.08
LP (1) C 40<br>s(6.91%)p13.48(93.07%)d 0.00(0.02%)	LP*(3)Th 197<br>s(0.48%)p 8.94(4.28%)d99.99(68.16%)f56.60(27.07%)g 0.03(0.01%)	16.97
LP (1) C 40<br>s(6.91%)p13.48(93.07%)d 0.00(0.02%)	LP*(4)Th 197<br>s(25.07%)p 0.16(3.91%)d 2.20(55.13%) f 0.63(15.88%)g 0.00(0.01%)	9.18
LP (2)Cl 180<br>s(0.29%)p99.99(99.68%)d 0.12(0.03%)	LP*(3)Th 197<br>s(0.48%)p 8.94(4.28%)d99.99(68.16%)f56.60(27.07%)g 0.03(0.01%)	14.41
LP (2)Cl 180<br>s(0.29%)p99.99(99.68%)d 0.12(0.03%)	LP*(10)Th 197<br>s(0.04%)p99.99(38.89%)d99.99(6.45%)f99.99(54.58%)g 1.11(0.04%)	10.28
LP (3)Cl 180<br>s(0.02%)p99.99(99.95%)d 1.74(0.03%)	LP*(3)Th 197<br>s(0.48%)p 8.94(4.28%)d99.99(68.16%)f56.60(27.07%)g 0.03(0.01%)	11.95
LP (3)Cl 180<br>s(0.02%)p99.99(99.95%)d 1.74(0.03%)	LP*(5)Th 197<br>s(5.05%)p 1.01(5.11%)d 0.92(4.64%) f16.86(85.19%)g 0.00(0.00%)	19.12

Table S12. Computed natural charges for **4**.

Atom Label	Natural charges
C28	-0.80542
C32	-0.75816
C33	-0.68956
C35	-0.74171
C39	-0.63529
C40	-0.79172
C47	-0.64349
C49	-0.53052
C51	-1.45570
Cl180	-0.28420
U197	0.86701

Table S13. Calculated Wiberg bond index for **4**.

Atom Label	Wiberg bond index
C28	0.4723
C32	0.4241
C33	0.3773
C35	0.2070
C39	0.3527
C40	0.3925
C47	0.3495
C49	0.2078
C51	0.6976
Cl180	1.1692
U197	0.0000

Table S14. Bonding orbitals from NBO analysis for **5**.

BD (1) C 16 - U 197<br>(77.49%) 0.8803* C 16 s(27.26%)p 2.67(72.73%)d 0.00(0.01%)<br>(22.51%) 0.4745* U 197 s(11.31%)p 0.07(0.78%)d 5.45(61.67%)f 2.32(26.24%)g 0.00(0.00%)
BD (1)Cl 178 - U 197<br>(83.03%) 0.9112*Cl 178 s(61.09%)p 0.64(38.87%)d 0.00(0.04%)<br>(16.97%) 0.4119* U 197 s(15.18%)p 0.71(10.75%)d 3.84(58.26%)f 1.04(15.80%)g 0.00(0.01%)

Table S15. Second order perturbation analysis for **5**.

Donor NBO	Acceptor NBO	E(2) kcal/mol
BD (1) C 11 - C 17<br>(50.15%) 0.7082* C 11 s(24.98%)p 3.00(74.96%)d 0.00(0.05%)<br>(49.85%) 0.7060* C 17 s(25.20%)p 2.97(74.75%)d 0.00(0.05%)	LP*(6) U 197<br>s(15.22%)p 0.06(0.95%)d 5.02(76.38%) f 0.49(7.45%)g 0.00(0.00%)	10.46
BD (1) C 11 - C 17<br>(50.15%) 0.7082* C 11 s(24.98%)p 3.00(74.96%)d 0.00(0.05%)<br>(49.85%) 0.7060* C 17 s(25.20%)p 2.97(74.75%)d 0.00(0.05%)	LP*(8) U 197<br>s(19.20%)p 1.44(27.64%)d 1.81(34.81%) f 0.96(18.34%)g 0.00(0.00%)	7.06
BD (1) C 11 - C 18<br>(50.04%) 0.7074* C 11 s(24.91%)p 3.01(75.05%)d 0.00(0.05%)<br>(49.96%) 0.7068* C 18 s(24.12%)p 3.14(75.82%)d 0.00(0.05%)	LP*(6) U 197<br>s(15.22%)p 0.06(0.95%)d 5.02(76.38%) f 0.49(7.45%)g 0.00(0.00%)	8.84
BD (1) C 11 - C 18<br>(50.04%) 0.7074* C 11 s(24.91%)p 3.01(75.05%)d 0.00(0.05%)<br>(49.96%) 0.7068* C 18 s(24.12%)p 3.14(75.82%)d 0.00(0.05%)	LP*(8) U 197<br>s(19.20%)p 1.44(27.64%)d 1.81(34.81%) f 0.96(18.34%)g 0.00(0.00%)	5.89
BD (1) C 17 - C 23<br>(49.87%) 0.7062* C 17 s(24.90%)p 3.01(75.05%)d 0.00(0.05%)	LP*(6) U 197<br>s(15.22%)p 0.06(0.95%)d 5.02(76.38%) f 0.49(7.45%)g 0.00(0.00%)	11.66

(50.13%) 0.7080* C 23 s(21.60%)p 3.63(78.34%)d 0.00(0.06%)		
BD (1) C 17 - C 23<br>(49.87%) 0.7062* C 17 s(24.90%)p 3.01(75.05%)d 0.00(0.05%)<br>(50.13%) 0.7080* C 23 s(21.60%)p 3.63(78.34%)d 0.00(0.06%)	LP*(8) U 197<br>s(19.20%)p 1.44(27.64%)d 1.81(34.81%) f 0.96(18.34%)g 0.00(0.00%)	7.13
BD (1) C 18 - C 23<br>(49.87%) 0.7062* C 18 s(22.20%)p 3.50(77.75%)d 0.00(0.05%<br>(50.13%) 0.7080* C 23 s(23.26%)p 3.30(76.69%)d 0.00(0.05%)	LP*(6) U 197<br>s(15.22%)p 0.06(0.95%)d 5.02(76.38%) f 0.49(7.45%)g 0.00(0.00%)	12.00
BD (1) C 18 - C 23<br>(49.87%) 0.7062* C 18 s(22.20%)p 3.50(77.75%)d 0.00(0.05%<br>(50.13%) 0.7080* C 23 s(23.26%)p 3.30(76.69%)d 0.00(0.05%)	LP*(8) U 197<br>s(19.20%)p 1.44(27.64%)d 1.81(34.81%) f 0.96(18.34%)g 0.00(0.00%)	8.13
BD (1) C 18 - C 23<br>(49.87%) 0.7062* C 18 s(22.20%)p 3.50(77.75%)d 0.00(0.05%<br>(50.13%) 0.7080* C 23 s(23.26%)p 3.30(76.69%)d 0.00(0.05%)	LP*(14) U 197<br>s(9.42%)p 0.18(1.68%)d 2.07(19.51%) f 7.37(69.38%)g 0.00(0.02%)	5.86
LP (1) C 17<br>s(12.37%)p 7.08(87.60%)d 0.00(0.04%)	LP*(3) U 197<br>s(0.01%)p 1.00(1.79%)d31.51(56.32%) f23.44(41.88%)g 0.00(0.00%)	33.91
LP (1) C 17<br>s(12.37%)p 7.08(87.60%)d 0.00(0.04%)	LP*(4) U 197<br>s(0.20%)p11.21(2.22%)d99.99(62.24%) f99.99(35.34%)g 0.01(0.00%)	9.51
LP (1) C 17<br>s(12.37%)p 7.08(87.60%)d 0.00(0.04%)	LP*(6) U 197<br>s(15.22%)p 0.06(0.95%)d 5.02(76.38%) f 0.49(7.45%)g 0.00(0.00%)	11.65
LP (1) C 17<br>s(12.37%)p 7.08(87.60%)d 0.00(0.04%)	LP*(8) U 197<br>s(19.20%)p 1.44(27.64%)d 1.81(34.81%) f 0.96(18.34%)g 0.00(0.00%)	10.19
LP (1) C 18<br>s(16.69%)p 4.99(83.28%)d 0.00(0.02%)	LP*(3) U 197<br>s(0.01%)p 1.00(1.79%)d31.51(56.32%) f23.44(41.88%)g 0.00(0.00%)	41.50
LP (1) C 18<br>s(16.69%)p 4.99(83.28%)d 0.00(0.02%)	LP*(4) U 197<br>s(0.20%)p11.21(2.22%)d99.99(62.24%) f99.99(35.34%)g 0.01(0.00%)	9.26
LP (1) C 18<br>s(16.69%)p 4.99(83.28%)d 0.00(0.02%)	LP*(6) U 197<br>s(15.22%)p 0.06(0.95%)d 5.02(76.38%) f 0.49(7.45%)g 0.00(0.00%)	8.76
LP (1) C 18<br>s(16.69%)p 4.99(83.28%)d 0.00(0.02%)	LP*(8) U 197<br>s(19.20%)p 1.44(27.64%)d 1.81(34.81%) f 0.96(18.34%)g 0.00(0.00%)	9.17
LP (1) C 23<br>s(18.45%)p 4.42(81.53%)d 0.00(0.02%)	LP*(3) U 197<br>s(0.01%)p 1.00(1.79%)d31.51(56.32%) f23.44(41.88%)g 0.00(0.00%)	16.91
LP (1) C 23<br>s(18.45%)p 4.42(81.53%)d 0.00(0.02%)	LP*(4) U 197<br>s(0.20%)p11.21(2.22%)d99.99(62.24%) f99.99(35.34%)g 0.01(0.00%)	42.53
LP (1) C 23	LP*(6) U 197	22.83

s(18.45%)p 4.42(81.53%)d 0.00(0.02%)	s(15.22%)p 0.06(0.95%)d 5.02(76.38%) f 0.49(7.45%)g 0.00(0.00%)	
LP (1) C 23<br>s(18.45%)p 4.42(81.53%)d 0.00(0.02%)	LP*(8) U 197<br>s(19.20%)p 1.44(27.64%)d 1.81(34.81%) f 0.96(18.34%)g 0.00(0.00%)	9.20
LP (1) C 29<br>s(5.30%)p17.87(94.67%)d 0.01(0.03%)	LP*(4) U 197<br>s(0.20%)p11.21(2.22%)d99.99(62.24%) f99.99(35.34%)g 0.01(0.00%)	11.37
LP (1) C 29<br>s(5.30%)p17.87(94.67%)d 0.01(0.03%)	LP*(5) U 197<br>s(1.54%)p 5.57(8.59%)d23.38(36.05%) f34.90(53.81%)g 0.00(0.00%)	13.97
LP (1) C 29<br>s(5.30%)p17.87(94.67%)d 0.01(0.03%)	LP*(7) U 197<br>s(11.13%)p 0.49(5.49%)d 2.77(30.79%) f 4.73(52.58%)g 0.00(0.00%)	13.57
LP (1) C 39<br>s(12.76%)p 6.84(87.22%)d 0.00(0.02%)	LP*(3) U 197<br>s(0.01%)p 1.00(1.79%)d31.51(56.32%) f23.44(41.88%)g 0.00(0.00%)	6.47
LP (1) C 39<br>s(12.76%)p 6.84(87.22%)d 0.00(0.02%)	LP*(4) U 197<br>s(0.20%)p11.21(2.22%)d99.99(62.24%) f99.99(35.34%)g 0.01(0.00%)	12.58
LP (1) C 39<br>s(12.76%)p 6.84(87.22%)d 0.00(0.02%)	LP*(5) U 197<br>s(1.54%)p 5.57(8.59%)d23.38(36.05%) f34.90(53.81%)g 0.00(0.00%)	24.38
LP (1) C 39<br>s(12.76%)p 6.84(87.22%)d 0.00(0.02%)	LP*(7) U 197<br>s(11.13%)p 0.49(5.49%)d 2.77(30.79%) f 4.73(52.58%)g 0.00(0.00%)	14.42
LP (3)Cl 178<br>s(0.00%)p 1.00(99.98%)d 0.00(0.02%)	LP*(7) U 197<br>s(11.13%)p 0.49(5.49%)d 2.77(30.79%) f 4.73(52.58%)g 0.00(0.00%)	7.08

Table S16. Computed natural charges for **5**.

Atom Label	Natural charges
C11	-0.63322
C16	-1.39758
C17	-0.64539
C18	-0.70770
C23	-0.72944
C29	-0.63783
C32	-0.74450
C37	-0.53190
C39	-0.71069
Cl178	-0.29230
U197	0.68942

Table S17. Calculated Wiberg bond index for **5**.

Atom Label	Wiberg bond index
C11	0.3863
C16	0.6981
C17	0.4536
C18	0.4813
C23	0.5438
C29	0.3995
C32	0.2116
C37	0.2310
C39	0.4511
Cl178	1.1364
U197	0.0000

Optimized coordinates

2

C	-3.123919000	-0.495341000	-4.328392000
C	-4.058629000	-1.698898000	-4.214694000
C	-1.773115000	-1.131768000	-4.067135000
C	0.620597000	-3.540661000	-3.636535000
C	2.055566000	-4.019496000	-3.847234000
C	-3.455388000	-2.456809000	-3.042695000
C	2.196123000	2.834164000	-2.687226000
C	-1.231605000	2.192194000	-2.868184000
C	2.311238000	-4.895839000	-2.620684000
C	-0.894114000	5.071946000	-1.737156000
C	-1.953376000	-5.161463000	-0.802838000
C	-2.933624000	-5.896333000	0.093996000
C	1.552998000	-4.137197000	-1.550843000
C	3.256178000	4.592984000	-0.452405000
C	3.952014000	1.571711000	-0.609899000
C	-3.254725000	3.253180000	-0.979579000
C	-3.127972000	-4.882127000	1.221470000
C	-3.159694000	-3.561152000	0.472857000
C	-0.341595000	2.715967000	0.092536000
C	1.105641000	2.523596000	0.251848000
C	3.225704000	-1.423820000	1.018760000
C	4.520429000	-1.381912000	1.813627000
C	-0.506227000	2.766020000	1.537586000
C	0.934446000	2.570840000	1.723138000
C	3.864780000	2.571462000	2.757781000
C	-2.340936000	5.099173000	2.180897000
C	-3.459755000	2.183569000	2.370339000
C	1.979318000	4.932370000	3.334729000
C	4.036982000	-1.231154000	3.272206000
C	2.553796000	-0.854804000	3.146625000
C	-1.538536000	3.267831000	4.457572000
C	1.642430000	2.240856000	4.795640000
H	-3.165243000	0.001758000	-5.300157000
H	-4.025765000	-2.301929000	-5.129054000
H	-1.372753000	-1.610362000	-4.969641000
H	-5.098323000	-1.421610000	-4.024725000
H	-3.346794000	0.241474000	-3.551613000
H	2.176950000	-4.550603000	-4.794329000

H	-0.117805000	-4.142714000	-4.173283000
H	-1.028466000	-0.453831000	-3.648867000
H	0.498153000	-2.490159000	-3.904429000
H	3.122451000	3.069204000	-3.224881000
H	-1.847202000	2.590276000	-3.683029000
H	2.738447000	-3.164343000	-3.845023000
H	-3.582322000	-3.539680000	-3.093617000
H	1.441662000	3.566649000	-2.981934000
H	1.866400000	1.839115000	-2.996886000
H	-0.210143000	2.067587000	-3.223362000
H	1.884684000	-5.895799000	-2.756581000
H	-1.995238000	-5.446645000	-1.856668000
H	-3.840428000	-2.090405000	-2.087595000
H	-1.457370000	5.473398000	-2.586730000
H	-1.617559000	1.212902000	-2.579625000
H	0.172553000	5.132342000	-1.971512000
H	-3.877551000	-6.076998000	-0.432351000
H	3.372454000	-5.001700000	-2.382670000
H	4.091472000	4.844015000	-1.115925000
H	-2.540058000	-6.856999000	0.435354000
H	3.717994000	0.703167000	-1.234081000
H	4.923562000	1.966931000	-0.925793000
H	-0.925925000	-5.272333000	-0.440041000
H	-3.771725000	3.696997000	-1.838962000
H	2.465623000	5.335723000	-0.601310000
H	-1.075227000	5.713043000	-0.868729000
H	1.250859000	-4.729208000	-0.687075000
H	2.100998000	-3.255673000	-1.203027000
H	-3.598519000	2.220397000	-0.873486000
H	-4.162505000	-3.300699000	0.117998000
H	3.602045000	4.685517000	0.580127000
H	-4.037927000	-5.046748000	1.803020000
H	4.032057000	1.246866000	0.427526000
H	-3.547757000	3.807931000	-0.087444000
H	3.294417000	-1.049271000	-0.001244000
H	-2.271866000	-4.899421000	1.903138000
H	2.768895000	-2.420509000	1.016943000
H	-2.745498000	-2.727744000	1.039029000
H	5.112359000	-0.514154000	1.510473000
H	-2.551752000	5.236975000	1.117817000
H	4.277018000	3.082997000	1.886132000
H	5.125104000	-2.278897000	1.657049000
H	-3.630172000	1.933833000	1.321983000
H	2.224491000	5.437842000	2.396343000
H	3.946144000	1.495970000	2.583229000
H	-1.476653000	5.725267000	2.427300000
H	-3.201132000	5.465910000	2.752145000
H	4.485978000	2.824273000	3.624195000
H	-4.361316000	2.670396000	2.759580000
H	-3.305223000	1.249472000	2.918197000
H	0.963607000	5.230070000	3.613421000
H	1.887373000	-1.685968000	3.397756000
H	2.665395000	5.285959000	4.112427000
H	4.600674000	-0.456636000	3.796733000
H	4.146578000	-2.162339000	3.833906000
H	2.258171000	0.022850000	3.717307000
H	-0.707565000	3.930831000	4.710171000
H	0.857709000	1.486824000	4.701547000
H	2.535223000	1.767135000	5.220316000

H	-1.298920000	2.256780000	4.791341000
H	-2.422299000	3.612656000	5.007117000
H	1.297488000	2.987986000	5.515721000
Cl	0.877934000	-0.603195000	-1.682421000
Cl	-2.314613000	-0.398444000	-0.403803000
Cl	0.044110000	-2.654565000	0.945979000
Cl	-0.859843000	-0.231635000	3.415148000
Mg	-0.934714000	-2.289496000	-1.28046700
O	-2.030163000	-2.170540000	-3.085459000
O	0.347440000	-3.695539000	-2.219440000
O	-2.327168000	-3.771126000	-0.706897000
O	2.361532000	-0.532782000	1.747479000
Si	-1.393032000	3.285601000	-1.332933000
Si	2.572799000	2.860960000	-0.831651000
Si	-1.939374000	3.290691000	2.612077000
Si	2.063094000	3.049178000	3.118437000
Th	0.074779000	0.304449000	0.976877000

3

C	0.363723000	3.058564000	-4.736560000
C	1.535967000	3.861130000	-4.167405000
C	-0.575755000	2.938887000	-3.541137000
C	2.190184000	-3.208937000	-3.464327000
C	1.100960000	-0.312742000	-3.442295000
C	-1.615447000	-2.488071000	-3.216971000
C	1.637868000	3.293130000	-2.765155000
C	3.762661000	-0.904898000	-2.228602000
C	-1.379627000	-5.109582000	-1.715988000
C	-3.270756000	3.217651000	-1.276706000
C	-4.118872000	2.697589000	-0.128153000
C	1.209605000	-2.101460000	-0.816220000
C	-3.257108000	-2.846411000	-0.737592000
C	-0.105842000	-2.645893000	-0.460363000
C	4.388442000	-3.934200000	-0.036259000
C	0.477023000	5.644893000	-0.446217000
C	1.696019000	6.432868000	0.004527000
C	-3.802209000	3.674576000	1.022659000
C	1.743622000	-2.532368000	0.467981000
C	1.609042000	4.413856000	1.248164000
C	-2.572809000	4.447846000	0.526560000
C	0.428263000	-3.081789000	0.839910000
C	1.904701000	5.894533000	1.421695000
C	4.421888000	-1.091448000	1.258329000
C	0.706211000	-6.036017000	1.535208000
C	-1.979714000	-4.627733000	2.042116000
C	-3.179565000	-0.526065000	1.921038000
C	3.523783000	-3.522233000	2.839297000
C	-4.148096000	-1.313241000	2.789838000
C	-1.908325000	-1.153197000	3.729459000
C	0.362975000	-4.044535000	3.866237000
C	-3.341212000	-1.575135000	4.080719000
H	-0.117774000	3.540445000	-5.590820000
H	2.460855000	3.729165000	-4.733894000
H	0.702806000	2.065660000	-5.043580000
H	1.298510000	4.930926000	-4.136563000
H	1.754225000	0.006078000	-4.263721000
H	2.631463000	-3.006068000	-4.446531000
H	-1.317896000	3.743641000	-3.512277000
H	-2.483232000	-2.911965000	-3.736127000

H	1.220462000	-3.691206000	-3.616103000
H	-0.726167000	-2.719969000	-3.806440000
H	0.160110000	-0.666599000	-3.862809000
H	-1.077006000	1.969990000	-3.483379000
H	4.196657000	-0.789026000	-3.229270000
H	-1.738117000	-1.403839000	-3.168917000
H	2.832380000	-3.924285000	-2.940894000
H	0.885061000	0.541280000	-2.798635000
H	2.161621000	2.333995000	-2.743975000
H	-2.162379000	-5.499020000	-2.376687000
H	2.090554000	3.956000000	-2.025772000
H	-0.407574000	-5.327689000	-2.170421000
H	-3.755256000	4.044464000	-1.814334000
H	-2.959335000	2.445107000	-1.978050000
H	4.432019000	-1.538991000	-1.645944000
H	3.720669000	0.078916000	-1.755076000
H	-5.181252000	2.658184000	-0.380871000
H	-4.017826000	-3.489364000	-1.194627000
H	-1.429239000	-5.654042000	-0.770077000
H	0.406436000	5.481936000	-1.523533000
H	4.433640000	-3.570210000	-1.065648000
H	-3.516037000	-1.807003000	-0.962516000
H	-3.776916000	1.691647000	0.121602000
H	2.561168000	6.194983000	-0.624464000
H	3.854579000	-4.890350000	-0.048781000
H	2.481138000	3.847311000	0.911562000
H	5.412713000	-4.119903000	0.305971000
H	-4.632419000	4.357135000	1.222984000
H	4.305558000	-0.468759000	0.370451000
H	-3.292203000	-2.996148000	0.341950000
H	0.435577000	-6.289013000	0.506182000
H	1.530982000	7.512462000	-0.034194000
H	-0.452055000	6.113107000	-0.101206000
H	2.912140000	6.068810000	1.806239000
H	-3.568117000	3.133739000	1.941149000
H	-2.369582000	-4.973197000	1.082916000
H	-3.300301000	-0.671694000	0.849742000
H	5.490650000	-1.277215000	1.415442000
H	1.796726000	-5.956250000	1.579153000
H	-2.834398000	5.464112000	0.204962000
H	-1.756445000	4.484557000	1.246963000
H	1.185587000	3.917751000	2.122263000
H	0.397495000	-6.859092000	2.189247000
H	4.035951000	-0.530699000	2.114339000
H	1.190260000	6.355373000	2.112713000
H	-2.474151000	-3.685492000	2.291925000
H	-3.192300000	0.546066000	2.149820000
H	-2.252222000	-5.367793000	2.802830000
H	-4.409377000	-2.254660000	2.299232000
H	3.032943000	-4.497985000	2.858996000
H	4.571105000	-3.668352000	3.128278000
H	-5.072618000	-0.758835000	2.970714000
H	3.050640000	-2.875492000	3.580386000
H	0.806453000	-3.054903000	3.992504000
H	1.079244000	-4.780898000	4.241381000
H	-1.143684000	-1.873367000	4.010303000
H	-1.645296000	-0.173013000	4.140805000
H	-0.533529000	-4.108944000	4.493244000
H	-3.383350000	-2.630119000	4.361068000

H	-3.717176000	-0.990616000	4.924272000
Cl	-1.415231000	0.467687000	-1.080737000
Cl	1.838463000	1.326855000	-0.140740000
Cl	-0.838261000	1.971904000	1.913119000
Cl	1.390839000	-0.519763000	3.323747000
Mg	-0.297391000	2.570117000	-0.40958400
O	0.261508000	3.084606000	-2.368739000
O	-2.090289000	3.730929000	-0.630679000
O	0.610988000	4.359860000	0.188446000
O	-1.890275000	-1.049507000	2.286668000
Si	2.021235000	-1.626251000	-2.426742000
Si	-1.549545000	-3.233012000	-1.477725000
Si	3.482017000	-2.730560000	1.124354000
Si	-0.096278000	-4.394599000	2.049471000
U	0.218763000	-0.623860000	1.000044000

8

C	-2.581950000	2.749830000	-5.867421000
C	-1.087675000	2.541895000	-5.525952000
C	-3.295749000	2.634077000	-4.512938000
C	-4.138949000	7.084053000	-4.959243000
C	-1.106798000	2.127330000	-4.061258000
C	-2.901081000	6.403286000	-4.411650000
C	-4.939157000	7.327735000	-3.677397000
C	1.082324000	-6.364227000	-3.304100000
C	-4.641479000	6.084291000	-2.841084000
C	4.064105000	3.028428000	-1.729286000
C	2.559210000	3.269084000	-1.795411000
C	3.331805000	-4.552796000	-2.523120000
C	0.517431000	-3.470429000	-2.434261000
C	-3.945982000	2.173288000	-1.087790000
C	4.503951000	3.875950000	-0.532049000
C	3.584401000	5.077343000	-0.645126000
C	-2.183897000	-5.557621000	-1.423146000
C	-0.648894000	-0.185043000	-1.401785000
C	-4.672821000	1.884031000	0.215656000
C	1.303413000	9.564710000	-1.799951000
C	2.801209000	-1.052604000	-0.994739000
C	-1.247674000	-8.490650000	-1.291434000
C	1.170637000	8.109891000	-2.235958000
C	4.790882000	-7.538070000	-0.719054000
C	-3.088591000	-1.588460000	-0.380045000
C	2.156960000	9.466220000	-0.535036000
C	-4.258824000	4.208601000	0.095451000
C	1.351751000	-5.603484000	-0.300184000
C	1.613280000	8.195875000	0.089876000
C	-4.254270000	3.064720000	1.090072000
C	4.840255000	-4.638903000	0.488649000
C	0.217322000	-6.336993000	0.352432000
C	2.283965000	-6.333909000	0.586605000
C	2.109213000	1.015647000	1.131009000
C	-0.343921000	-2.363712000	0.883079000
C	-2.748546000	-7.271765000	1.031708000
C	1.347875000	4.299386000	1.881519000
C	-1.568341000	0.399321000	1.463080000
C	1.180113000	-7.033417000	1.201087000
C	1.001492000	-1.869697000	1.462462000
C	0.199899000	3.742165000	2.732440000
C	4.045162000	-1.287719000	1.747074000

C	-0.122438000	6.051411000	2.162778000
C	2.216625000	-9.828185000	1.824492000
C	-3.427548000	-4.131902000	1.994880000
C	4.843876000	-7.096891000	2.278548000
C	-0.815683000	-2.523993000	2.334045000
C	-0.879050000	4.840070000	2.678941000
C	0.373733000	-1.885848000	2.772032000
C	-0.563796000	-9.115035000	2.779150000
C	-0.894248000	-5.108105000	3.547490000
C	1.803863000	-7.813961000	4.163804000
C	2.537816000	-0.551785000	4.659294000
C	-0.436710000	0.158294000	4.881102000
C	-2.904399000	-2.788166000	4.621831000
C	0.622738000	-2.590091000	5.815089000
H	-2.955777000	1.998463000	-6.567222000
H	-2.749028000	3.730876000	-6.319103000
H	-0.615353000	1.782029000	-6.152737000
H	-0.530228000	3.474980000	-5.636000000
H	-4.677685000	6.411255000	-5.635498000
H	-3.741300000	1.640729000	-4.378287000
H	-4.047974000	3.401332000	-4.332408000
H	-3.905684000	8.003872000	-5.500630000
H	-2.353592000	5.782446000	-5.121096000
H	-1.249905000	1.049177000	-3.935852000
H	-2.205038000	7.114315000	-3.954045000
H	1.360788000	-6.062055000	-4.320395000
H	-0.247624000	2.451408000	-3.477178000
H	-6.009383000	7.458035000	-3.853252000
H	4.554736000	3.379618000	-2.642760000
H	2.172256000	3.377240000	-2.810329000
H	3.316669000	-4.164287000	-3.548340000
H	0.664319000	-3.160200000	-3.475720000
H	-4.566333000	8.221732000	-3.168050000
H	-4.485536000	1.841982000	-1.976957000
H	0.012215000	-6.584061000	-3.297836000
H	-5.410557000	5.312163000	-2.931339000
H	1.614267000	-7.290760000	-3.064640000
H	4.293841000	1.968119000	-1.605108000
H	-2.939674000	1.750961000	-1.091806000
H	3.989872000	-5.424337000	-2.508134000
H	3.944739000	5.806426000	-1.381105000
H	1.986546000	2.504945000	-1.268979000
H	-1.669029000	-5.498663000	-2.384445000
H	0.847929000	-2.657319000	-1.786669000
H	1.752044000	10.186979000	-2.577763000
H	5.560663000	4.151696000	-0.565643000
H	-0.550679000	-3.625021000	-2.270470000
H	3.782966000	-3.793498000	-1.884301000
H	-0.446355000	-0.943558000	-2.161735000
H	-4.480928000	6.316258000	-1.786283000
H	-1.375816000	0.527211000	-1.802306000
H	-3.237783000	-5.784248000	-1.616940000
H	-0.524732000	-8.383431000	-2.103225000
H	4.371957000	-7.281824000	-1.695370000
H	1.940440000	7.804365000	-2.949689000
H	2.012826000	-0.696652000	-1.659414000
H	3.720574000	-0.498664000	-1.220794000
H	-5.757821000	1.880358000	0.066436000
H	-4.377224000	0.918835000	0.630070000

H	-3.599116000	-0.832082000	-0.987732000
H	3.215914000	9.344235000	-0.786929000
H	-2.205105000	-8.814580000	-1.716198000
H	-2.133484000	-4.571024000	-0.952630000
H	-2.993259000	-2.490493000	-0.992170000
H	2.990084000	-2.101426000	-1.230580000
H	0.267121000	0.368578000	-1.193942000
H	5.883994000	-7.502772000	-0.790187000
H	4.315921000	3.345320000	0.406599000
H	0.319446000	9.978789000	-1.556742000
H	3.395860000	5.587714000	0.301755000
H	4.498027000	-8.570713000	-0.500935000
H	2.298590000	7.685972000	0.767934000
H	4.042945000	-3.930184000	0.246480000
H	-0.885342000	-9.286001000	-0.631167000
H	-5.268783000	4.608640000	-0.058503000
H	0.190558000	7.861096000	-2.643509000
H	-3.721550000	-1.833537000	0.474163000
H	5.607772000	-4.560172000	-0.287423000
H	-3.576443000	5.024031000	0.338878000
H	2.055653000	10.331394000	0.124504000
H	-3.245882000	2.905515000	1.481968000
H	-0.378899000	-3.211190000	0.162501000
H	2.985566000	1.601280000	0.828672000
H	1.237719000	1.359638000	0.565596000
H	1.676585000	3.621085000	1.097859000
H	-3.734740000	-6.970847000	0.662624000
H	-1.762292000	1.364972000	0.990078000
H	-0.172519000	2.805385000	2.317713000
H	-4.930901000	3.241934000	1.929438000
H	0.665711000	8.381507000	0.609661000
H	4.811278000	-1.558261000	1.013932000
H	-3.062867000	-4.415853000	1.006586000
H	1.752344000	-10.234919000	0.919787000
H	5.269934000	-4.326309000	1.444133000
H	-2.785907000	-8.340773000	1.250734000
H	3.225497000	-9.504765000	1.557927000
H	-0.629501000	0.471530000	2.016182000
H	-0.739032000	6.762628000	1.614168000
H	3.908613000	-2.145458000	2.409874000
H	1.917956000	1.216157000	2.189635000
H	0.535534000	3.551206000	3.754365000
H	-4.116262000	-3.288940000	1.887471000
H	-2.561310000	-6.738343000	1.963225000
H	5.936141000	-7.140147000	2.188600000
H	-2.356490000	0.164319000	2.182119000
H	2.208304000	4.595552000	2.493241000
H	4.488282000	-8.103181000	2.509437000
H	-0.991154000	-9.531206000	1.863216000
H	-3.996756000	-4.976687000	2.392640000
H	4.421863000	-0.451599000	2.343278000
H	0.430704000	6.565187000	2.960874000
H	2.297221000	-10.635471000	2.561012000
H	-1.653705000	4.571780000	1.956633000
H	4.595073000	-6.434276000	3.110942000
H	-1.322993000	-6.110763000	3.502250000
H	-1.350146000	5.026162000	3.647022000
H	-1.257151000	-8.370864000	3.176111000
H	-0.482721000	-9.926927000	3.510841000

H	2.751418000	0.249522000	3.948334000
H	-0.252797000	1.022231000	4.235042000
H	2.782930000	-7.337886000	4.091778000
H	-3.458079000	-1.927539000	4.226040000
H	-1.465636000	-0.172368000	4.716694000
H	3.271133000	-1.348791000	4.517615000
H	-0.388183000	-4.981106000	4.517830000
H	1.111470000	-7.082429000	4.593403000
H	1.878161000	-8.659151000	4.858080000
H	-3.623356000	-3.463541000	5.099430000
H	-2.224022000	-2.423623000	5.393910000
H	2.661373000	-0.148634000	5.671073000
H	-0.337097000	0.481981000	5.923092000
H	1.421201000	-3.324243000	5.679003000
H	-0.332962000	-3.113932000	5.774694000
H	0.726668000	-2.142317000	6.810087000
Cl	-0.013999000	5.186398000	-3.300302000
Cl	-0.669477000	3.265050000	-0.591523000
Cl	-1.790146000	6.507148000	-0.778148000
Cl	3.069321000	-4.294886000	3.750019000
Mg	-2.126596000	4.402936000	-2.17635700
Mg	0.479703000	5.425165000	-0.850691000
O	-2.267450000	2.799749000	-3.523677000
O	-3.415832000	5.527374000	-3.383001000
O	2.334516000	4.529812000	-1.099305000
O	-3.813350000	3.625438000	-1.158314000
O	1.371860000	7.319536000	-1.029925000
O	0.835088000	5.494733000	1.237383000
Si	1.551760000	-5.018105000	-2.043509000
Si	-1.429585000	-6.885665000	-0.284704000
Si	-1.388398000	-0.954055000	0.162382000
Si	4.138188000	-6.403506000	0.663458000
Si	2.429043000	-0.847869000	0.851343000
Si	1.157342000	-8.391282000	2.478311000
Si	-2.027673000	-3.687279000	3.188010000
Si	0.788753000	-1.244654000	4.500041000
Th	1.023779000	-4.484285000	1.986894000

9

C	1.883717000	-6.253128000	-5.509088000
C	3.990433000	-4.042331000	-5.313724000
C	1.095212000	-3.324595000	-5.191928000
C	-2.851356000	3.183971000	-3.863551000
C	5.423049000	-7.100886000	-3.642395000
C	-1.425932000	-5.136187000	-3.379615000
C	-1.615966000	2.465026000	-3.321785000
C	-0.354396000	-7.867071000	-2.651799000
C	-0.278425000	-1.216594000	-2.895466000
C	-2.475347000	9.037713000	-0.986743000
C	2.359131000	-5.016738000	-2.788076000
C	-2.924270000	4.436485000	-2.993946000
C	-0.702078000	3.629530000	-3.009981000
C	6.060626000	-4.191165000	-2.699620000
C	-1.946387000	7.929505000	-1.913215000
C	2.752085000	-1.760618000	-2.311473000
C	3.392095000	-5.581484000	-1.937055000
C	1.321419000	-5.432559000	-1.840303000
C	1.291571000	1.056975000	-1.702042000
C	-1.538329000	8.983842000	0.235423000

C	1.682748000	5.617462000	-1.682392000
C	2.831178000	4.629935000	-1.783964000
C	2.376495000	-6.016444000	-0.961240000
C	3.207303000	-8.942468000	-0.774030000
C	-1.411313000	-5.942273000	-0.463716000
C	6.175416000	-6.527074000	-0.756634000
C	-2.588323000	-2.058538000	-0.437914000
C	-0.337034000	8.217018000	-0.287555000
C	0.974020000	-1.565420000	-0.102076000
C	3.017039000	4.218808000	-0.321949000
C	1.586528000	4.153508000	0.190041000
C	0.267565000	-2.799542000	0.470961000
C	-5.145054000	2.130329000	-0.352708000
C	0.884578000	-8.088586000	0.937026000
C	4.337626000	0.268948000	0.329362000
C	-4.527248000	0.944465000	0.409449000
C	1.842448000	-1.554876000	1.027232000
C	3.657074000	-7.072942000	1.664087000
C	1.369691000	-2.812832000	1.552454000
C	-5.575011000	3.070906000	0.758173000
C	-1.261083000	-0.593955000	1.947786000
C	-2.318939000	-3.454957000	2.235446000
C	-4.251342000	1.498796000	1.812397000
C	1.990986000	1.258955000	2.099534000
C	0.418111000	-5.190370000	3.221540000
C	-3.844432000	6.845818000	3.403513000
C	4.024281000	-0.754573000	3.154979000
C	-0.873240000	2.553250000	3.564228000
C	3.158403000	-4.000999000	3.860806000
C	-5.127989000	7.490138000	2.864234000
C	-6.153652000	6.335256000	2.845502000
C	-5.445613000	5.223304000	3.609498000
C	0.646479000	-2.389606000	4.535654000
C	-2.941306000	2.789571000	4.714058000
C	-0.798064000	1.800839000	4.887683000
C	-2.267160000	1.526398000	5.216667000
H	2.018167000	-6.163580000	-6.593071000
H	3.877960000	-3.936129000	-6.399137000
H	0.984269000	-3.407517000	-6.279485000
H	0.839416000	-6.521382000	-5.320550000
H	2.507449000	-7.076713000	-5.148647000
H	4.816143000	-4.729820000	-5.127515000
H	1.466234000	-2.322097000	-4.966203000
H	4.267414000	-3.065875000	-4.907560000
H	0.108383000	-3.431204000	-4.743552000
H	-2.709279000	3.448449000	-4.916538000
H	4.901205000	-6.792677000	-4.551934000
H	-1.021955000	-5.267668000	-4.386417000
H	-1.172808000	1.762145000	-4.029576000
H	-3.762848000	2.586253000	-3.786518000
H	0.186010000	-7.935389000	-3.600686000
H	6.480275000	-7.252101000	-3.888791000
H	-0.068129000	-0.987824000	-3.943631000
H	-2.445231000	-5.539906000	-3.374748000
H	5.003697000	-8.064316000	-3.335275000
H	5.318910000	-3.420575000	-2.923617000
H	-2.431097000	10.009247000	-1.486084000
H	-0.209451000	4.017761000	-3.910416000
H	-3.511026000	8.847137000	-0.699275000

H	-1.493313000	-4.063560000	-3.176008000
H	-1.361535000	-8.272950000	-2.800542000
H	-3.235208000	5.329108000	-3.544727000
H	2.899766000	-1.975679000	-3.369027000
H	-2.702763000	7.195414000	-2.186476000
H	-0.547724000	-2.272843000	-2.834291000
H	6.720904000	-4.293747000	-3.566941000
H	-1.847412000	1.928699000	-2.397715000
H	-1.484073000	8.332105000	-2.821551000
H	-1.137723000	-0.620605000	-2.576810000
H	1.413048000	1.482472000	-2.705311000
H	0.169599000	-8.502894000	-1.934264000
H	1.089270000	5.728261000	-2.591654000
H	-3.576896000	4.311072000	-2.128779000
H	3.650691000	-1.269660000	-1.906494000
H	0.043384000	3.410896000	-2.248649000
H	2.536639000	3.762288000	-2.382846000
H	6.649092000	-3.839501000	-1.847907000
H	2.549950000	-9.160724000	-1.621968000
H	3.725858000	5.072626000	-2.228145000
H	-1.273347000	9.972231000	0.618230000
H	4.201596000	-8.734921000	-1.176161000
H	-1.994619000	8.401893000	1.039567000
H	-2.481796000	-2.900189000	-1.128024000
H	2.026816000	6.608678000	-1.362242000
H	-2.352929000	-1.147363000	-0.991296000
H	0.357506000	1.421346000	-1.259858000
H	2.123023000	1.395136000	-1.081177000
H	7.218352000	-6.666830000	-1.065704000
H	-2.153115000	-6.744534000	-0.393593000
H	-1.945851000	-4.988923000	-0.428335000
H	5.799192000	-7.489034000	-0.402936000
H	-3.635529000	-2.016510000	-0.120342000
H	3.271146000	-9.839504000	-0.147561000
H	-4.381417000	2.623313000	-0.958749000
H	0.338092000	8.850614000	-0.878551000
H	0.159025000	-8.376726000	0.173673000
H	0.119854000	-3.699707000	-0.180054000
H	3.913285000	0.562509000	-0.632186000
H	-0.763738000	-6.002346000	0.412390000
H	6.160493000	-5.813546000	0.070232000
H	-5.977345000	1.839239000	-0.998081000
H	3.526830000	3.259514000	-0.210539000
H	1.146730000	3.165377000	0.063823000
H	-3.605165000	0.605957000	-0.061894000
H	0.225271000	7.679452000	0.475707000
H	5.012748000	-0.574099000	0.156377000
H	3.592681000	4.977749000	0.218645000
H	-5.212469000	0.094998000	0.459360000
H	4.927297000	1.105840000	0.722678000
H	1.113304000	-8.980880000	1.531881000
H	0.409051000	-7.366190000	1.601349000
H	-1.246504000	0.229606000	1.229743000
H	-5.603724000	4.121745000	0.472386000
H	4.483962000	-6.426860000	1.364031000
H	1.456981000	4.476174000	1.223242000
H	-2.424431000	-4.448813000	1.793190000
H	1.222689000	1.447693000	1.345527000
H	4.072271000	-7.986888000	2.103164000

H	-3.326306000	-3.070138000	2.437494000
H	-2.081048000	-0.450513000	2.659332000
H	-6.533306000	2.771611000	1.204810000
H	-2.945253000	7.073289000	2.833856000
H	3.101102000	-6.548383000	2.446075000
H	-0.245389000	-5.236705000	2.356245000
H	-3.214605000	1.397875000	2.123155000
H	-0.316694000	-0.584191000	2.496255000
H	2.597548000	2.161035000	2.236490000
H	1.097997000	-6.043246000	3.167879000
H	-1.800633000	-3.562287000	3.189998000
H	-4.906773000	1.053296000	2.569993000
H	1.485313000	1.047911000	3.046965000
H	-0.779512000	1.894374000	2.702376000
H	3.809474000	-4.267718000	3.024783000
H	4.614024000	-1.648323000	2.940476000
H	-6.343357000	6.017827000	1.817244000
H	-3.675353000	7.086640000	4.459878000
H	-4.970871000	7.882360000	1.857840000
H	-0.156206000	3.369847000	3.471090000
H	4.709568000	0.055216000	3.430369000
H	-0.192196000	-5.307379000	4.122758000
H	3.375675000	-0.953507000	4.011580000
H	-5.709599000	4.211522000	3.305864000
H	-0.412755000	-2.205684000	4.333258000
H	3.102553000	-4.855771000	4.543982000
H	3.623534000	-3.172365000	4.397549000
H	1.166860000	-1.425908000	4.500452000
H	-2.858058000	3.615971000	5.430289000
H	-5.448867000	8.313282000	3.507682000
H	-7.108915000	6.602262000	3.303884000
H	-0.212651000	0.883449000	4.796863000
H	-5.580659000	5.326594000	4.694737000
H	-0.342969000	2.422202000	5.665629000
H	0.739338000	-2.786685000	5.552691000
H	-3.990619000	2.661760000	4.438849000
H	-2.626445000	0.657113000	4.656823000
H	-2.445659000	1.349514000	6.279987000
Cl	-3.646818000	5.764167000	0.059262000
Cl	4.904981000	-3.346944000	0.586414000
Cl	-1.668351000	2.953984000	0.231630000
Cl	-0.912417000	5.748050000	1.990045000
Mg	-1.224635000	5.273387000	-0.48008400
Mg	-2.908905000	4.267118000	1.943841000
O	-1.578392000	4.662867000	-2.488427000
O	-0.924720000	7.237445000	-1.164952000
O	0.828085000	5.069942000	-0.658563000
O	-4.542874000	2.918693000	1.754984000
O	-4.054845000	5.418953000	3.308144000
O	-2.214651000	3.129188000	3.520368000
Si	2.334227000	-4.637258000	-4.608386000
Si	-0.401862000	-6.057114000	-2.071960000
Si	5.217095000	-5.834838000	-2.235147000
Si	1.225767000	-0.848201000	-1.811974000
Si	2.516989000	-7.460174000	0.197498000
Si	-1.444121000	-2.252774000	1.061047000
Si	3.054776000	-0.245491000	1.622495000
Si	1.415794000	-3.577960000	3.251198000
U	2.566696000	-3.581659000	-0.615344000

7

U	7.489354000	3.811747000	13.330076000
Cl	6.385072000	3.292404000	10.727669000
Si	8.359065000	6.894690000	16.100803000
Si	3.685929000	4.831472000	14.016327000
Si	5.965716000	4.026029000	17.106617000
Si	9.789939000	1.798196000	10.123428000
Si	5.995269000	7.699872000	12.993862000
Si	9.575777000	0.772404000	14.111926000
Si	11.567034000	3.974060000	14.248149000
Si	9.964339000	5.599474000	10.971260000
C	10.245270000	7.133876000	12.018593000
H	10.068578000	8.040774000	11.434773000
H	11.257150000	7.175098000	12.419797000
H	9.540415000	7.119368000	12.851287000
C	8.428526000	5.941605000	9.902436000
H	8.169464000	5.103118000	9.255148000
H	8.594528000	6.828716000	9.279604000
C	11.465745000	5.430335000	9.817669000
H	12.376110000	5.283248000	10.407754000
H	11.588384000	6.349391000	9.232810000
H	11.379107000	4.595119000	9.119235000
C	9.507943000	2.723205000	11.724866000
C	9.713338000	4.103635000	12.051163000
C	9.566392000	2.317531000	13.099366000
C	7.288942000	5.828883000	15.007869000
C	9.864955000	3.755961000	13.526986000
C	7.223293000	7.656259000	17.428009000
H	6.328584000	8.081908000	16.964869000
H	7.745226000	8.456086000	17.965897000
H	6.901780000	6.914576000	18.163019000
C	3.260810000	4.447358000	12.210803000
H	4.015159000	4.832957000	11.525445000
H	2.281445000	4.855823000	11.934358000
H	3.228279000	3.362543000	12.096684000
C	11.670237000	1.651756000	9.915118000
H	12.091729000	1.089856000	10.753960000
H	12.156105000	2.629350000	9.897468000
H	11.918444000	1.122527000	8.987649000
C	5.502019000	5.041555000	14.300631000
C	6.430757000	6.107544000	13.854845000
C	6.381961000	4.759929000	15.446214000
C	9.707676000	5.905405000	16.989596000
H	9.518279000	4.839023000	16.866409000
H	9.736611000	6.132631000	18.059801000
H	10.692196000	6.117825000	16.571634000
C	2.868500000	3.422288000	14.989586000
H	3.222669000	2.462671000	14.612160000
H	1.788372000	3.500590000	14.812800000
H	3.039715000	3.469112000	16.066173000
C	5.341495000	2.223976000	17.046645000
H	4.953862000	1.983068000	16.057716000
H	4.560447000	2.070543000	17.800666000
H	6.168943000	1.541340000	17.263396000
C	7.388895000	3.959053000	18.354210000
H	8.212263000	3.343412000	17.982589000
H	7.005437000	3.488405000	19.267558000
H	7.787014000	4.938047000	18.621682000

C	2.709643000	6.375310000	14.555990000
H	2.950144000	6.631940000	15.592580000
H	1.633914000	6.168629000	14.498334000
H	2.918104000	7.251654000	13.938931000
C	9.167042000	0.013135000	10.124420000
H	9.804790000	-0.589547000	10.776266000
H	9.258212000	-0.401271000	9.113489000
H	8.134823000	-0.089769000	10.461801000
C	9.165705000	8.407992000	15.293784000
H	9.859891000	8.173014000	14.486446000
H	9.731317000	8.925549000	16.077592000
H	8.417484000	9.108985000	14.915360000
C	9.119456000	2.658131000	8.568231000
H	9.338712000	2.035601000	7.692111000
H	9.592774000	3.628927000	8.408594000
H	8.041618000	2.821647000	8.639156000
C	4.580288000	5.062996000	17.901135000
H	4.891629000	6.105155000	18.006215000
H	4.326321000	4.677194000	18.895543000
H	3.675160000	5.053020000	17.288710000
C	4.738796000	7.424357000	11.593416000
H	5.146842000	6.723994000	10.857610000
H	4.571903000	8.381905000	11.087052000
H	3.774020000	7.045361000	11.930581000
C	11.283371000	-0.036404000	14.337857000
H	11.635123000	-0.395319000	13.36356000
H	11.208253000	-0.905229000	15.00286800
H	12.043665000	0.635982000	14.736791000
C	5.252030000	8.882586000	14.287559000
H	4.608122000	8.353930000	14.992958000
H	4.670264000	9.680955000	13.813039000
H	6.057852000	9.352536000	14.860493000
C	8.523842000	-0.645643000	13.425904000
H	7.523838000	-0.311657000	13.144890000
H	8.453430000	-1.414139000	14.203915000
H	8.986502000	-1.109362000	12.554049000
C	12.169045000	5.770683000	14.403800000
H	11.360859000	6.476543000	14.595748000
H	12.686185000	6.079002000	13.490756000
H	12.887606000	5.842290000	15.227887000
C	7.349265000	8.714630000	12.143037000
H	8.189783000	8.956766000	12.792199000
H	6.885422000	9.658111000	11.829574000
H	7.735091000	8.218819000	11.251521000
C	11.815009000	3.199403000	15.969677000
H	12.881979000	3.263966000	16.214695000
H	11.530301000	2.148184000	16.022934000
H	11.266260000	3.739743000	16.742985000
C	8.810562000	1.299830000	15.769125000
H	9.262736000	2.230383000	16.121397000
H	8.916746000	0.547473000	16.558462000
H	7.738914000	1.485367000	15.628966000
C	12.826201000	3.139630000	13.093163000
H	12.627855000	2.071523000	12.982314000
H	13.848430000	3.265746000	13.467222000
H	12.767071000	3.591417000	12.099001000
Cl	2.819658000	1.045172000	11.118082000
Cl	5.663719000	-0.607713000	10.991565000
Cl	5.575053000	1.614762000	13.636798000

Mg	4.157568000	-0.198416000	12.89132100
Mg	4.904598000	1.601110000	10.08744300
O	3.024216000	-1.940640000	12.358449000
O	2.532234000	0.104757000	14.234029000
O	5.090009000	-1.532545000	14.242427000
O	5.334279000	0.743524000	8.236335000
O	3.948389000	2.952776000	8.754087000
O	1.023285000	0.969588000	7.586978000
C	1.911671000	-4.009611000	12.496266000
H	1.003144000	-4.485802000	12.872938000
H	2.726177000	-4.740998000	12.543026000
C	1.205223000	0.474739000	13.788154000
H	0.987261000	-0.105973000	12.890309000
H	1.214580000	1.535441000	13.526277000
C	2.272847000	-2.755191000	13.277908000
H	1.378783000	-2.203472000	13.585209000
H	2.893534000	-2.931876000	14.159403000
C	0.299087000	0.180488000	14.970187000
H	0.008790000	-0.876687000	14.985839000
H	-0.612196000	0.783060000	14.946387000
C	1.774359000	-3.460237000	11.075129000
H	1.849364000	-4.229162000	10.302658000
H	0.815214000	-2.945149000	10.954425000
C	5.721444000	-2.737977000	13.741815000
H	6.428026000	-2.460877000	12.956883000
H	4.929075000	-3.349312000	13.306323000
C	2.922626000	-2.468049000	11.016162000
H	3.873491000	-2.947039000	10.761770000
H	2.765251000	-1.620153000	10.348452000
C	6.250084000	-0.290336000	6.341642000
H	6.666330000	-1.269169000	6.091464000
H	6.703392000	0.443756000	5.671210000
C	1.206588000	0.508504000	16.155462000
H	1.240635000	1.588494000	16.320066000
H	0.899360000	0.023846000	17.085295000
C	6.017807000	-2.499410000	16.136047000
H	5.101105000	-2.864622000	16.611832000
H	6.798215000	-2.439118000	16.898187000
C	2.565489000	0.018255000	15.677902000
H	3.384280000	0.640225000	16.041198000
H	2.746459000	-1.029485000	15.944604000
C	4.705898000	-0.258059000	6.226008000
H	4.293618000	-1.194922000	5.842381000
H	4.385033000	0.546995000	5.559943000
C	5.760232000	-1.163101000	15.469686000
H	5.105087000	-0.494353000	16.021665000
H	6.679374000	-0.626671000	15.231523000
C	4.761831000	3.673819000	7.811440000
H	4.389222000	3.465957000	6.798708000
H	5.785250000	3.310710000	7.901752000
C	6.410162000	-3.391941000	14.948870000
H	6.095859000	-4.429259000	15.089034000
H	7.493901000	-3.389412000	14.808439000
C	2.731089000	3.712445000	8.914944000
H	2.371444000	3.523849000	9.924778000
H	2.006458000	3.332256000	8.187437000
C	0.157190000	0.993152000	8.717238000
H	-0.836300000	1.357000000	8.412946000
H	0.582996000	1.682445000	9.447070000

C	0.076104000	-0.458473000	9.229368000
H	-0.955549000	-0.741491000	9.457100000
H	0.670882000	-0.574936000	10.138052000
C	3.105764000	5.181076000	8.655781000
H	2.468578000	5.604972000	7.874465000
H	2.988836000	5.789686000	9.554370000
C	4.579150000	5.125839000	8.212031000
H	5.245851000	5.345696000	9.049062000
H	4.806924000	5.816763000	7.396646000
C	6.525729000	0.062208000	7.806281000
H	6.662924000	-0.826015000	8.431835000
H	7.361943000	0.740674000	7.964093000
C	0.660475000	-1.288092000	8.065916000
H	0.042540000	-2.148840000	7.796625000
H	1.658031000	-1.658089000	8.320474000
C	0.761478000	-0.261636000	6.934990000
H	1.572363000	-0.450757000	6.226729000
H	-0.185039000	-0.204254000	6.372813000
C	4.238876000	0.019073000	7.652247000
H	3.344239000	0.637944000	7.739936000
H	4.102364000	-0.911266000	8.220961000
H	7.564381000	6.137142000	10.544994000
**6**			
Th	7.490562000	3.755180000	13.374470000
Cl	6.365054000	3.262114000	10.736096000
Si	8.358274000	6.905584000	16.103964000
Si	3.649487000	4.857297000	14.051364000
Si	5.947120000	4.067103000	17.153164000
Si	9.824201000	1.758239000	10.101270000
Si	5.973012000	7.703384000	13.011012000
Si	9.594467000	0.781273000	14.137750000
Si	11.631232000	3.951151000	14.201138000
Si	9.962973000	5.576714000	10.951765000
C	10.205420000	7.105431000	12.015707000
H	10.018323000	8.016096000	11.441476000
H	11.209621000	7.161155000	12.432846000
H	9.488610000	7.065002000	12.838192000
C	8.420201000	5.907886000	9.887779000
H	8.160957000	5.067878000	9.242296000
H	8.576730000	6.795548000	9.263178000
C	11.464548000	5.429659000	9.794957000
H	12.373135000	5.267320000	10.383894000
H	11.593226000	6.354260000	9.220440000
H	11.372253000	4.602469000	9.086953000
C	9.572031000	2.687294000	11.703577000
C	9.738471000	4.077163000	12.026349000
C	9.663270000	2.285097000	13.071529000
C	7.275855000	5.840423000	15.030320000
C	9.921208000	3.732784000	13.502513000
C	7.260579000	7.680460000	17.456437000
H	6.368410000	8.126162000	17.006818000
H	7.805249000	8.467446000	17.990812000
H	6.931818000	6.943006000	18.192656000
C	3.241822000	4.436352000	12.248294000
H	3.997256000	4.819235000	11.562129000
H	2.260641000	4.830095000	11.957288000
H	3.221374000	3.349161000	12.152039000
C	11.697353000	1.615032000	9.834227000
H	12.154986000	1.095407000	10.681188000

H	12.172144000	2.595555000	9.753533000
H	11.917341000	1.048131000	8.921941000
C	5.458597000	5.087812000	14.347998000
C	6.412781000	6.114358000	13.870678000
C	6.336190000	4.810613000	15.491060000
C	9.735783000	5.919935000	16.954484000
H	9.550168000	4.851027000	16.846635000
H	9.809117000	6.156000000	18.020845000
H	10.701121000	6.134718000	16.493298000
C	2.841545000	3.450469000	15.039786000
H	3.204102000	2.488508000	14.675270000
H	1.761021000	3.516735000	14.859969000
H	3.009472000	3.512592000	16.116396000
C	5.329386000	2.261251000	17.090633000
H	4.918153000	2.027103000	16.109745000
H	4.566443000	2.094920000	17.860227000
H	6.164775000	1.580013000	17.280229000
C	7.399558000	3.998001000	18.366116000
H	8.207790000	3.373160000	17.976739000
H	7.039189000	3.538364000	19.294335000
H	7.813807000	4.975759000	18.612816000
C	2.644717000	6.393276000	14.562476000
H	2.856527000	6.649494000	15.605659000
H	1.571927000	6.181033000	14.476416000
H	2.865188000	7.272634000	13.953811000
C	9.197608000	-0.026082000	10.136916000
H	9.843613000	-0.620273000	10.788687000
H	9.270725000	-0.458892000	9.132165000
H	8.170167000	-0.119347000	10.492495000
C	9.157849000	8.409614000	15.272992000
H	9.832641000	8.157876000	14.454201000
H	9.744030000	8.928131000	16.040919000
H	8.409267000	9.114545000	14.902724000
C	9.110390000	2.615024000	8.564004000
H	9.300789000	1.992365000	7.681311000
H	9.585839000	3.583066000	8.392205000
H	8.036115000	2.786290000	8.664749000
C	4.571740000	5.097802000	17.970298000
H	4.889319000	6.137638000	18.081590000
H	4.319908000	4.704854000	18.962331000
H	3.664724000	5.097486000	17.360186000
C	4.707641000	7.420040000	11.618904000
H	5.117277000	6.721016000	10.882563000
H	4.532535000	8.375613000	11.111491000
H	3.746602000	7.035937000	11.960957000
C	11.264488000	-0.026540000	14.547439000
H	11.688597000	-0.438872000	13.62488600
H	11.125961000	-0.856049000	15.25120800
H	11.995038000	0.664401000	14.969393000
C	5.233819000	8.887628000	14.305448000
H	4.597104000	8.353183000	15.013465000
H	4.645322000	9.684210000	13.836268000
H	6.040983000	9.359424000	14.875011000
C	8.565693000	-0.653402000	13.450795000
H	7.572853000	-0.329030000	13.132897000
H	8.472354000	-1.410881000	14.237105000
H	9.056860000	-1.128802000	12.600665000
C	12.270217000	5.738603000	14.295406000
H	11.490951000	6.456155000	14.556054000

H	12.710621000	6.039761000	13.340684000
H	13.057545000	5.798283000	15.055354000
C	7.320207000	8.709195000	12.139617000
H	8.167736000	8.954106000	12.778497000
H	6.857063000	9.650779000	11.819553000
H	7.696030000	8.201099000	11.250319000
C	11.859684000	3.246523000	15.957663000
H	12.930208000	3.080696000	16.127369000
H	11.348983000	2.298041000	16.127865000
H	11.509458000	3.953980000	16.711501000
C	8.714786000	1.432486000	15.697331000
H	9.124634000	2.409540000	15.973789000
H	8.797908000	0.776679000	16.570468000
H	7.640374000	1.556525000	15.501763000
C	12.871444000	3.050986000	13.074363000
H	12.682935000	1.975349000	13.046168000
H	13.902032000	3.214347000	13.409032000
H	12.778651000	3.429634000	12.052405000
Cl	2.795968000	1.023506000	11.123389000
Cl	5.660223000	-0.608009000	11.001715000
Cl	5.509260000	1.577327000	13.672685000
Mg	4.126425000	-0.237151000	12.88817300
Mg	4.873197000	1.580706000	10.08479700
O	3.014750000	-1.973523000	12.341527000
O	2.513686000	0.069824000	14.237571000
O	5.074754000	-1.562923000	14.241195000
O	5.328115000	0.738677000	8.239754000
O	3.932701000	2.949730000	8.763618000
O	1.030220000	0.970301000	7.574755000
C	1.895928000	-4.039740000	12.464182000
H	0.982705000	-4.514392000	12.831219000
H	2.707907000	-4.773534000	12.516293000
C	1.188255000	0.459811000	13.804643000
H	0.956454000	-0.111784000	12.904356000
H	1.207298000	1.522576000	13.550843000
C	2.253465000	-2.788794000	13.252959000
H	1.358988000	-2.234637000	13.554790000
H	2.867344000	-2.969773000	14.138354000
C	0.290247000	0.166640000	14.992584000
H	-0.001609000	-0.890119000	15.008812000
H	-0.619801000	0.771314000	14.976917000
C	1.772604000	-3.486275000	11.043305000
H	1.852645000	-4.253523000	10.269693000
H	0.815850000	-2.968570000	10.915369000
C	5.718563000	-2.758989000	13.734934000
H	6.427170000	-2.470212000	12.955762000
H	4.933429000	-3.373094000	13.290412000
C	2.923311000	-2.496296000	10.996244000
H	3.875598000	-2.975929000	10.748618000
H	2.772539000	-1.646652000	10.329212000
C	6.325043000	-0.162386000	6.331709000
H	6.787654000	-1.094524000	5.998756000
H	6.779454000	0.655600000	5.767307000
C	1.208412000	0.492144000	16.170407000
H	1.245660000	1.572043000	16.334775000
H	0.907811000	0.008006000	17.102635000
C	6.027443000	-2.517879000	16.128161000
H	5.118063000	-2.883878000	16.616904000
H	6.816838000	-2.451535000	16.880512000

C	2.563472000	0.000014000	15.682440000
H	3.383976000	0.628665000	16.030768000
H	2.751429000	-1.044205000	15.957355000
C	4.786106000	-0.161999000	6.149934000
H	4.414059000	-1.103703000	5.738062000
H	4.475443000	0.641738000	5.477849000
C	5.756603000	-1.185181000	15.459418000
H	5.105277000	-0.516084000	16.015681000
H	6.671369000	-0.646717000	15.208357000
C	4.750429000	3.681145000	7.831681000
H	4.377718000	3.488912000	6.815831000
H	5.772068000	3.312235000	7.918310000
C	6.408434000	-3.415446000	14.940451000
H	6.087009000	-4.449936000	15.084991000
H	7.491350000	-3.421790000	14.794192000
C	2.719203000	3.713891000	8.940011000
H	2.366964000	3.518862000	9.951360000
H	1.987458000	3.342252000	8.215345000
C	0.155997000	0.981732000	8.698527000
H	-0.837387000	1.342060000	8.389543000
H	0.572770000	1.668245000	9.436208000
C	0.079779000	-0.473502000	9.199846000
H	-0.950056000	-0.760356000	9.430836000
H	0.679627000	-0.594307000	10.104584000
C	3.097758000	5.183006000	8.690033000
H	2.464891000	5.611788000	7.907876000
H	2.978093000	5.787598000	9.590950000
C	4.572456000	5.127827000	8.251854000
H	5.236637000	5.333458000	9.094460000
H	4.806504000	5.829192000	7.447313000
C	6.521276000	0.047850000	7.832580000
H	6.584231000	-0.896246000	8.384783000
H	7.368820000	0.674464000	8.101701000
C	0.661708000	-1.293544000	8.028149000
H	0.037090000	-2.145195000	7.745672000
H	1.654568000	-1.676642000	8.282043000
C	0.774959000	-0.254837000	6.908966000
H	1.592893000	-0.437758000	6.207022000
H	-0.165493000	-0.191659000	6.337426000
C	4.246879000	0.079640000	7.560559000
H	3.370129000	0.725729000	7.624447000
H	4.043383000	-0.866142000	8.081916000
H	7.560100000	6.099698000	10.537703000

TS

U	7.835033000	5.163341000	12.929018000
Cl	6.251219000	4.073517000	10.983129000
Si	8.219538000	8.871625000	14.056179000
Si	4.028295000	5.064674000	14.328074000
Si	6.669839000	6.360301000	16.831702000
Si	10.051112000	1.792265000	11.482401000
Si	5.277841000	7.760108000	11.584367000
Si	9.761383000	3.551810000	14.837377000
Si	12.069785000	6.285970000	12.390318000
Si	9.804435000	5.057309000	9.342418000
C	10.090275000	6.912637000	9.077597000
H	9.941951000	7.154627000	8.019209000
H	11.112460000	7.193921000	9.341563000
H	9.398352000	7.523739000	9.661970000

C	8.250632000	4.600228000	8.350567000
H	7.854252000	3.618830000	8.616729000
H	8.505005000	4.600961000	7.284101000
C	11.275162000	4.167097000	8.501341000
H	11.868443000	4.884667000	7.923967000
H	10.929035000	3.390198000	7.814189000
H	11.936077000	3.698320000	9.233712000
C	9.373726000	3.464740000	12.011168000
C	9.700254000	4.702507000	11.171382000
C	10.185662000	4.113492000	13.118347000
C	7.115259000	7.385421000	13.995439000
C	10.546001000	5.183566000	12.245738000
C	7.370279000	10.319365000	14.941963000
H	6.435964000	10.578931000	14.435775000
H	8.010180000	11.208457000	14.961370000
H	7.121885000	10.055622000	15.974068000
C	3.484943000	3.951562000	12.883926000
H	3.628570000	4.467467000	11.935072000
H	2.442547000	3.635224000	12.974217000
H	4.131867000	3.070558000	12.980185000
C	11.911881000	1.755729000	11.829102000
H	12.123348000	1.872194000	12.894412000
H	12.396315000	2.583828000	11.303028000
H	12.360146000	0.819605000	11.478337000
C	5.620989000	5.948534000	14.038696000
C	6.051801000	6.964027000	13.072724000
C	6.686071000	6.370842000	14.962403000
C	9.856788000	8.500987000	14.934067000
H	9.724200000	8.333279000	16.002423000
H	10.555448000	9.335410000	14.810735000
H	10.320635000	7.606928000	14.510408000
C	4.150553000	3.845473000	15.772912000
H	4.897400000	3.077119000	15.548608000
H	3.179871000	3.357271000	15.910053000
H	4.419712000	4.318823000	16.716237000
C	6.846287000	4.647061000	17.665804000
H	6.995277000	3.838870000	16.946495000
H	5.964530000	4.409611000	18.268879000
H	7.708149000	4.655487000	18.340449000
C	7.962331000	7.469964000	17.667832000
H	8.984959000	7.104118000	17.561001000
H	7.726029000	7.480165000	18.738445000
H	7.916307000	8.501670000	17.312557000
C	2.597001000	6.265729000	14.669584000
H	2.781558000	6.843205000	15.578588000
H	1.650279000	5.725959000	14.788838000
H	2.483231000	6.979430000	13.849227000
C	9.193128000	0.390133000	12.428047000
H	9.345630000	0.439320000	13.508350000
H	9.577548000	-0.573253000	12.072871000
H	8.115896000	0.429293000	12.241765000
C	8.643882000	9.436888000	12.296599000
H	9.063161000	8.614323000	11.710014000
H	9.392410000	10.235601000	12.325247000
H	7.767176000	9.821299000	11.770831000
C	9.834935000	1.378957000	9.636384000
H	9.351688000	0.403480000	9.526090000
H	10.815524000	1.322731000	9.156231000
H	9.246311000	2.115146000	9.090105000

C	5.015469000	7.116801000	17.392546000
H	4.838879000	8.054510000	16.856158000
H	5.036276000	7.333446000	18.466620000
H	4.166665000	6.456349000	17.201182000
C	3.678770000	6.908499000	11.025144000
H	3.878463000	5.901961000	10.656598000
H	3.252878000	7.495995000	10.203269000
H	2.930099000	6.847213000	11.817832000
C	10.729744000	2.064320000	15.496517000
H	10.767798000	1.238788000	14.783760000
H	10.277497000	1.703001000	16.426253000
H	11.758184000	2.372040000	15.713989000
C	4.792937000	9.547862000	11.999478000
H	4.108234000	9.551314000	12.853949000
H	4.290159000	10.028741000	11.152809000
H	5.659194000	10.156591000	12.269115000
C	7.961318000	3.085743000	14.320718000
H	8.284612000	3.082438000	12.885103000
H	7.795068000	2.008778000	14.379491000
C	11.908868000	8.133960000	12.022879000
H	11.340936000	8.659671000	12.789367000
H	11.435866000	8.326987000	11.058966000
H	12.916719000	8.563721000	11.994622000
C	6.398647000	7.753856000	10.052362000
H	7.322834000	8.313458000	10.205172000
H	5.878432000	8.194776000	9.193781000
H	6.663498000	6.724188000	9.796327000
C	12.785427000	6.062104000	14.127320000
H	13.803243000	6.464277000	14.165231000
H	12.828160000	4.999091000	14.382206000
H	12.194121000	6.577928000	14.887236000
C	9.922494000	4.934693000	16.104734000
H	10.972119000	5.220751000	16.212061000
H	9.546574000	4.619069000	17.080023000
H	9.358866000	5.818017000	15.795187000
C	13.332511000	5.589505000	11.159491000
H	13.501726000	4.524113000	11.338522000
H	14.290301000	6.110274000	11.267647000
H	12.996261000	5.710752000	10.126742000
Cl	2.430694000	1.035730000	11.124703000
Cl	4.582165000	-1.809294000	10.084142000
Cl	5.517432000	0.958461000	12.541841000
Mg	3.828966000	-0.814436000	12.12925900
Mg	4.446623000	2.428318000	10.89597800
O	2.077601000	-2.047776000	12.117244000
O	3.179391000	-0.394112000	14.146253000
O	4.994091000	-2.331247000	13.102357000
O	5.229920000	1.473240000	9.285490000
O	3.517157000	3.728074000	9.543238000
O	1.340563000	1.528942000	6.815064000
C	0.021506000	-3.088757000	12.593149000
H	-0.930288000	-2.949999000	13.112267000
H	0.287563000	-4.150703000	12.640480000
C	2.380860000	0.759965000	14.467133000
H	1.491056000	0.730781000	13.838073000
H	2.945616000	1.665446000	14.229771000
C	1.129611000	-2.231433000	13.184600000
H	0.748812000	-1.252268000	13.491405000
H	1.649307000	-2.685453000	14.030923000

C	2.138395000	0.623284000	15.956149000
H	1.378418000	-0.142571000	16.148427000
H	1.812526000	1.557775000	16.419078000
C	0.007579000	-2.606621000	11.142456000
H	-0.475817000	-3.304581000	10.454605000
H	-0.500240000	-1.638996000	11.066412000
C	4.533145000	-3.695364000	12.993000000
H	4.061490000	-3.819055000	12.013206000
H	3.793996000	-3.861002000	13.782049000
C	1.489541000	-2.447501000	10.852891000
H	1.959357000	-3.386195000	10.542447000
H	1.729938000	-1.680949000	10.118868000
C	6.850860000	0.747711000	7.796032000
H	7.674425000	0.052775000	7.620341000
H	7.097746000	1.702330000	7.318415000
C	3.518049000	0.167186000	16.438254000
H	4.161303000	1.038192000	16.587813000
H	3.481992000	-0.393402000	17.375581000
C	6.827586000	-3.670133000	12.439991000
H	7.855022000	-3.921445000	12.714469000
H	6.722971000	-3.733242000	11.352834000
C	4.038455000	-0.685299000	15.276009000
H	5.067805000	-0.441122000	15.003754000
H	3.963996000	-1.758849000	15.463574000
C	5.502320000	0.211390000	7.301201000
H	5.434093000	-0.862145000	7.494548000
H	5.344247000	0.389822000	6.233967000
C	6.433261000	-2.279432000	12.905812000
H	6.887983000	-2.009769000	13.865558000
H	6.648284000	-1.500052000	12.175976000
C	4.225159000	4.357643000	8.442450000
H	5.035549000	3.691421000	8.144650000
H	4.659740000	5.291864000	8.807766000
C	5.781884000	-4.551486000	13.123525000
H	6.036969000	-4.709332000	14.177749000
H	5.661217000	-5.528248000	12.648058000
C	2.086100000	3.856944000	9.359610000
H	1.655851000	4.171885000	10.313245000
H	1.699825000	2.872652000	9.088839000
C	0.095322000	1.276171000	7.448569000
H	-0.703743000	1.190112000	6.693408000
H	-0.133833000	2.123127000	8.100252000
C	0.296239000	-0.045004000	8.194742000
H	-0.639628000	-0.596661000	8.321280000
H	0.718342000	0.155989000	9.182431000
C	1.920450000	4.863888000	8.234508000
H	0.987090000	4.712339000	7.687203000
H	1.937565000	5.885449000	8.627217000
C	3.161331000	4.590244000	7.383750000
H	3.422409000	5.416947000	6.718351000
H	2.999557000	3.689295000	6.783760000
C	6.576452000	0.956913000	9.271583000
H	6.594618000	0.017667000	9.831518000
H	7.198709000	1.701117000	9.766313000
C	1.321641000	-0.790437000	7.310998000
H	0.888601000	-1.666502000	6.820000000
H	2.177166000	-1.132142000	7.899918000
C	1.738084000	0.273747000	6.277125000
H	2.812959000	0.313426000	6.089576000

H	1.226911000	0.102989000	5.316592000
C	4.479704000	0.947284000	8.167203000
H	4.013327000	1.795256000	7.661596000
H	3.706255000	0.288075000	8.559922000
H	7.451828000	5.326553000	8.514795000
H	7.127138000	3.562416000	14.857060000

TS

Th	7.642947000	5.129434000	12.933344000
Cl	6.195650000	4.176029000	10.720119000
Si	8.235827000	8.839235000	13.916309000
Si	3.805178000	5.219930000	14.067158000
Si	6.179634000	6.703376000	16.705223000
Si	9.911363000	1.628693000	11.968737000
Si	5.403262000	7.868000000	11.309170000
Si	9.409637000	3.553091000	15.232299000
Si	12.122364000	5.995158000	12.947282000
Si	10.175400000	4.755173000	9.689132000
C	10.706182000	6.551696000	9.394763000
H	10.695533000	6.756600000	8.318462000
H	11.720553000	6.737523000	9.755741000
H	10.032358000	7.264176000	9.878038000
C	8.710253000	4.428234000	8.535910000
H	8.233169000	3.470794000	8.748264000
H	9.069969000	4.419046000	7.500431000
C	11.603896000	3.682237000	9.028840000
H	11.814726000	3.968579000	7.991546000
H	11.367321000	2.615610000	9.038774000
H	12.518732000	3.826842000	9.608224000
C	9.283575000	3.344099000	12.380281000
C	9.801614000	4.514937000	11.503482000
C	9.997458000	4.029606000	13.533703000
C	6.964488000	7.493093000	13.887803000
C	10.544934000	5.000423000	12.647362000
C	7.531556000	10.489949000	14.531970000
H	6.696174000	10.810289000	13.903299000
H	8.293937000	11.276707000	14.519604000
H	7.156738000	10.396064000	15.555582000
C	3.527094000	3.926361000	12.703680000
H	3.553456000	4.413490000	11.727974000
H	2.571622000	3.411787000	12.831277000
H	4.340528000	3.195541000	12.800266000
C	11.799233000	1.595782000	12.054450000
H	12.129336000	1.885483000	13.056340000
H	12.230552000	2.300240000	11.340926000
H	12.190446000	0.597117000	11.832852000
C	5.387130000	6.153383000	13.876836000
C	5.941957000	7.108902000	12.912531000
C	6.413082000	6.541462000	14.861450000
C	9.708471000	8.358906000	15.002602000
H	9.431173000	8.253553000	16.050467000
H	10.501645000	9.111123000	14.939408000
H	10.118181000	7.402619000	14.666848000
C	3.769193000	4.169640000	15.642625000
H	4.628741000	3.494982000	15.689399000
H	2.855668000	3.564757000	15.616439000
H	3.751509000	4.760093000	16.558386000
C	6.207016000	5.071558000	17.704043000
H	6.470819000	4.209882000	17.086490000

H	5.241438000	4.868918000	18.175796000
H	6.952747000	5.148337000	18.501690000
C	7.425740000	7.839025000	17.576718000
H	8.422488000	7.399582000	17.654268000
H	7.057096000	7.998915000	18.596837000
H	7.506164000	8.817782000	17.098614000
C	2.259686000	6.321641000	14.081160000
H	2.395712000	7.176502000	14.748773000
H	1.386591000	5.752144000	14.420384000
H	2.043850000	6.706469000	13.081627000
C	9.230059000	0.327381000	13.173674000
H	9.481423000	0.510654000	14.218377000
H	9.635828000	-0.652101000	12.893466000
H	8.142369000	0.290756000	13.075336000
C	8.910811000	9.087874000	12.160366000
H	9.350544000	8.155299000	11.790652000
H	9.701406000	9.845068000	12.151962000
H	8.137849000	9.401742000	11.456201000
C	9.280439000	1.039844000	10.287144000
H	8.200911000	0.897875000	10.373526000
H	9.737040000	0.077434000	10.028712000
H	9.477855000	1.741269000	9.475566000
C	4.508042000	7.567614000	16.987627000
H	4.487741000	8.500118000	16.414007000
H	4.361409000	7.811374000	18.045958000
H	3.660934000	6.960714000	16.659944000
C	3.704028000	7.223832000	10.769015000
H	3.667709000	6.133282000	10.770539000
H	3.489160000	7.575269000	9.753211000
H	2.919185000	7.602381000	11.428168000
C	10.398112000	2.179175000	16.091142000
H	10.812303000	1.454196000	15.388677000
H	9.781976000	1.648241000	16.824507000
H	11.234803000	2.641968000	16.625975000
C	5.199521000	9.744268000	11.515051000
H	4.530249000	9.951440000	12.356074000
H	4.767136000	10.188376000	10.611014000
H	6.149698000	10.245808000	11.713480000
C	7.670364000	3.062782000	14.573272000
H	8.126718000	3.013559000	13.189170000
H	7.466445000	1.991365000	14.618193000
C	12.165510000	7.827692000	12.481526000
H	11.521032000	8.429294000	13.120874000
H	11.874745000	8.003461000	11.444838000
H	13.194664000	8.183295000	12.607601000
C	6.594552000	7.557624000	9.854501000
H	7.643039000	7.547749000	10.165842000
H	6.487397000	8.334856000	9.089124000
H	6.379805000	6.588648000	9.397857000
C	12.566245000	5.822124000	14.778977000
H	13.601743000	6.139679000	14.940651000
H	12.476572000	4.778909000	15.095905000
H	11.926101000	6.436132000	15.416284000
C	9.363725000	5.004996000	16.430431000
H	10.373271000	5.391943000	16.589777000
H	8.952495000	4.700510000	17.395853000
H	8.743930000	5.814692000	16.038697000
C	13.495941000	5.148789000	11.952102000
H	13.498476000	4.069354000	12.123243000

H	14.471404000	5.547802000	12.251258000
H	13.377110000	5.327036000	10.880261000
Cl	2.552649000	1.002424000	11.161340000
Cl	4.439327000	-1.674204000	9.570806000
Cl	5.864242000	0.980847000	11.884674000
Mg	4.181991000	-0.834665000	11.80011400
Mg	4.442536000	2.460463000	10.59188700
O	2.510412000	-2.098512000	12.161841000
O	4.074978000	-0.478223000	13.929860000
O	5.538558000	-2.388489000	12.385154000
O	4.941435000	1.683059000	8.764449000
O	3.285408000	3.802259000	9.480799000
O	0.532332000	1.563906000	7.241178000
C	0.646615000	-3.199754000	13.081160000
H	-0.145753000	-3.106374000	13.828104000
H	0.923695000	-4.257032000	13.003600000
C	3.376758000	0.656883000	14.478814000
H	2.337497000	0.594914000	14.153162000
H	3.814785000	1.575293000	14.078390000
C	1.861673000	-2.351749000	13.422326000
H	1.566180000	-1.395661000	13.865720000
H	2.580188000	-2.837708000	14.085681000
C	3.608067000	0.536016000	15.970760000
H	2.977930000	-0.256108000	16.391342000
H	3.407462000	1.466428000	16.504335000
C	0.265277000	-2.646510000	11.707803000
H	-0.369267000	-3.320140000	11.126894000
H	-0.253916000	-1.687660000	11.812571000
C	5.076757000	-3.753514000	12.273170000
H	4.384981000	-3.809512000	11.427461000
H	4.551822000	-4.005004000	13.198888000
C	1.624964000	-2.439732000	11.064469000
H	2.008908000	-3.348147000	10.589577000
H	1.666114000	-1.626304000	10.343109000
C	6.158124000	0.968528000	6.918808000
H	6.908576000	0.265933000	6.548706000
H	6.299645000	1.928036000	6.408675000
C	5.084421000	0.138147000	15.996779000
H	5.709819000	1.029371000	15.885702000
H	5.381697000	-0.370737000	16.916955000
C	7.175799000	-3.614606000	11.200915000
H	8.239800000	-3.863981000	11.197622000
H	6.812941000	-3.575648000	10.169673000
C	5.224679000	-0.762488000	14.768656000
H	6.133742000	-0.556689000	14.200440000
H	5.187369000	-1.827666000	15.010589000
C	4.724116000	0.455019000	6.746935000
H	4.685096000	-0.618740000	6.945830000
H	4.324157000	0.646438000	5.747454000
C	6.899923000	-2.288443000	11.885528000
H	7.555311000	-2.114879000	12.745245000
H	6.952831000	-1.430077000	11.215479000
C	3.837355000	4.569921000	8.378257000
H	4.579169000	3.946168000	7.876262000
H	4.338561000	5.442326000	8.800425000
C	6.326320000	-4.582469000	12.024765000
H	6.825375000	-4.828151000	12.969143000
H	6.101088000	-5.514995000	11.501009000
C	1.837576000	3.824900000	9.437622000

H	1.481244000	3.920871000	10.465055000
H	1.495221000	2.878872000	9.010832000
C	-0.549061000	1.267786000	8.113337000
H	-1.481430000	1.161239000	7.534743000
H	-0.664957000	2.104952000	8.806719000
C	-0.164860000	-0.050900000	8.792145000
H	-1.042297000	-0.645160000	9.062574000
H	0.407026000	0.156824000	9.699482000
C	1.501427000	5.000132000	8.538997000
H	0.514256000	4.895457000	8.082775000
H	1.535292000	5.937555000	9.102812000
C	2.639159000	4.943844000	7.517664000
H	2.796229000	5.889012000	6.992070000
H	2.432313000	4.165259000	6.775962000
C	6.239363000	1.155392000	8.419771000
H	6.379063000	0.206126000	8.943886000
H	6.975345000	1.884427000	8.754664000
C	0.729986000	-0.742190000	7.737800000
H	0.285231000	-1.662938000	7.349947000
H	1.706999000	-0.998985000	8.156780000
C	0.860258000	0.321190000	6.632779000
H	1.864841000	0.405708000	6.213719000
H	0.158874000	0.115376000	5.808577000
C	3.945171000	1.199880000	7.829948000
H	3.408069000	2.071173000	7.448225000
H	3.256059000	0.556757000	8.376854000
H	7.948745000	5.204287000	8.633662000
H	6.831382000	3.572991000	15.069703000

5

U	1.365507000	1.676914000	0.693643000
Cl	-0.266278000	0.912576000	-1.443573000
Si	1.746646000	5.807593000	1.144971000
Si	-2.240367000	2.003780000	2.164899000
Si	0.720398000	3.601115000	4.259439000
Si	3.858207000	-1.660363000	-1.359216000
Si	-1.276087000	4.370471000	-0.867435000
Si	2.370434000	-0.964805000	2.450712000
Si	5.334503000	1.765634000	1.632598000
Si	3.790955000	2.069472000	-2.234533000
C	3.566368000	3.902188000	-1.827392000
H	3.397785000	4.480962000	-2.741794000
H	4.422880000	4.338396000	-1.308789000
H	2.688928000	4.033234000	-1.185052000
C	2.475209000	1.651210000	-3.531706000
H	2.280816000	0.580013000	-3.604397000
H	2.791566000	2.012715000	-4.516420000
C	5.525034000	1.841970000	-2.976532000
H	6.279740000	2.284297000	-2.320906000
H	5.589611000	2.347229000	-3.946867000
H	5.785124000	0.790591000	-3.123297000
C	2.785915000	-0.313881000	-0.525464000
C	3.439438000	1.071686000	-0.703077000
C	3.207174000	-0.230532000	0.939923000
C	0.888463000	4.153054000	1.245124000
C	3.973865000	0.931850000	0.636091000
C	0.806403000	7.028308000	2.260308000
H	-0.232419000	7.099352000	1.924155000
H	1.249664000	8.029127000	2.209303000

H	0.789087000	6.715995000	3.306019000
C	-2.736770000	0.691495000	0.871407000
H	-2.795183000	1.149008000	-0.116351000
H	-3.696446000	0.223419000	1.108120000
H	-1.956294000	-0.079176000	0.885029000
C	5.635925000	-1.520286000	-0.751022000
H	5.692952000	-1.745474000	0.315676000
H	6.021101000	-0.509130000	-0.906130000
H	6.276620000	-2.221889000	-1.295435000
C	-0.655398000	2.850721000	1.718029000
C	-0.321446000	3.677208000	0.571591000
C	0.559543000	3.314179000	2.423999000
C	3.572360000	5.760269000	1.650527000
H	4.018599000	6.749965000	1.503787000
H	4.118283000	5.050273000	1.023053000
H	3.718187000	5.473552000	2.691798000
C	-2.257697000	1.076449000	3.814826000
H	-1.554534000	0.241338000	3.808989000
H	-3.270387000	0.684791000	3.967325000
H	-2.021945000	1.723856000	4.661273000
C	0.904535000	2.032783000	5.312543000
H	0.088960000	1.320283000	5.180902000
H	0.936476000	2.315641000	6.371139000
H	1.839498000	1.520675000	5.074040000
C	2.180096000	4.682993000	4.813332000
H	3.143528000	4.188713000	4.678600000
H	2.052362000	4.866756000	5.886603000
H	2.218043000	5.652994000	4.316755000
C	-3.643519000	3.279501000	2.292982000
H	-3.487240000	3.895440000	3.184038000
H	-4.622936000	2.795100000	2.380610000
H	-3.664154000	3.953495000	1.434330000
C	3.143621000	-3.364647000	-0.964286000
H	3.362599000	-3.668353000	0.060220000
H	3.558763000	-4.112976000	-1.648294000
H	2.057072000	-3.344907000	-1.086103000
C	1.745365000	6.638249000	-0.564245000
H	2.449662000	6.177662000	-1.258158000
H	2.065462000	7.675564000	-0.409817000
H	0.764071000	6.666312000	-1.038600000
C	3.857005000	-1.558196000	-3.253007000
H	2.855675000	-1.690666000	-3.669095000
H	4.479172000	-2.378749000	-3.629391000
H	4.265094000	-0.623787000	-3.639666000
C	-0.841658000	4.521507000	4.837121000
H	-1.011748000	5.398443000	4.205414000
H	-0.744474000	4.857219000	5.875594000
H	-1.731584000	3.890372000	4.769790000
C	-2.849063000	3.414421000	-1.326032000
H	-2.606375000	2.390493000	-1.605297000
H	-3.296753000	3.915185000	-2.193142000
H	-3.595727000	3.395236000	-0.529924000
C	2.286478000	-2.857349000	2.380300000
H	1.687484000	-3.185515000	1.529601000
H	1.827567000	-3.228649000	3.303524000
H	3.290046000	-3.289354000	2.300838000
C	-1.919328000	6.099499000	-0.403655000
H	-2.544932000	6.016395000	0.491563000
H	-2.534192000	6.514536000	-1.210705000

H	-1.123543000	6.812851000	-0.182579000
C	0.767342000	-0.043900000	2.354321000
H	1.739006000	-0.511178000	-0.814937000
H	-0.044612000	-0.636074000	1.917614000
C	6.364017000	2.920501000	0.550736000
H	5.763376000	3.707693000	0.091647000
H	6.867051000	2.362191000	-0.243218000
H	7.132893000	3.403303000	1.163240000
C	-0.260275000	4.443432000	-2.468708000
H	0.774781000	4.739985000	-2.293695000
H	-0.701043000	5.151759000	-3.179435000
H	-0.253379000	3.451710000	-2.930442000
C	4.595857000	2.758731000	3.067012000
H	5.171206000	3.673851000	3.233368000
H	4.599025000	2.186680000	3.997788000
H	3.563442000	3.055851000	2.858536000
C	3.336876000	-0.553822000	4.023761000
H	4.284987000	-1.097921000	4.067525000
H	2.739491000	-0.842097000	4.895223000
H	3.549248000	0.512447000	4.105585000
C	6.494161000	0.432423000	2.305686000
H	5.929383000	-0.362905000	2.797121000
H	7.191007000	0.860362000	3.034206000
H	7.077102000	-0.014952000	1.495859000
Cl	-3.729091000	-2.342409000	-0.748899000
Cl	-1.705924000	-5.087488000	-2.234355000
Cl	-0.415765000	-2.362207000	-0.078758000
Mg	-2.120951000	-4.173942000	-0.05148400
Mg	-1.863232000	-0.924909000	-1.41760200
O	-3.864792000	-5.376372000	0.219824000
O	-2.278334000	-3.712813000	2.052216000
O	-0.862951000	-5.749137000	0.658557000
O	-1.213155000	-1.748063000	-3.173276000
O	-3.124919000	0.254348000	-2.606012000
O	-4.995485000	-2.340350000	-5.592812000
C	-6.007614000	-5.995185000	0.976029000
H	-6.859844000	-5.653099000	1.568693000
H	-5.922798000	-7.081802000	1.089215000
C	-2.916860000	-2.520047000	2.558561000
H	-3.965927000	-2.550869000	2.263231000
H	-2.450291000	-1.644749000	2.101344000
C	-4.714105000	-5.308425000	1.383041000
H	-4.890398000	-4.255897000	1.623839000
H	-4.189376000	-5.780722000	2.215951000
C	-2.655322000	-2.564783000	4.049097000
H	-3.308655000	-3.300510000	4.532772000
H	-2.802514000	-1.595095000	4.526847000
C	-6.095715000	-5.615506000	-0.502010000
H	-6.765112000	-6.257275000	-1.080011000
H	-6.427755000	-4.577347000	-0.608867000
C	-1.371080000	-7.099536000	0.610156000
H	-1.972055000	-7.210626000	-0.298094000
H	-2.001849000	-7.247646000	1.490264000
C	-4.649860000	-5.742617000	-0.945814000
H	-4.383199000	-6.769067000	-1.219881000
H	-4.363933000	-5.080506000	-1.760398000
C	0.237626000	-2.388993000	-4.867588000
H	1.094012000	-3.009695000	-5.141584000
H	0.310899000	-1.438313000	-5.407578000

C	-1.198827000	-3.025951000	4.060321000
H	-0.538870000	-2.180941000	3.834584000
H	-0.885231000	-3.464870000	5.010803000
C	0.806143000	-7.133548000	-0.291374000
H	1.859272000	-7.401722000	-0.178133000
H	0.530103000	-7.213416000	-1.347127000
C	-1.156902000	-4.037329000	2.919487000
H	-0.238422000	-3.971745000	2.338873000
H	-1.298924000	-5.068345000	3.255457000
C	-1.106378000	-3.076566000	-5.133934000
H	-1.037446000	-4.140241000	-4.891192000
H	-1.434423000	-2.969356000	-6.170913000
C	0.516893000	-5.726059000	0.202867000
H	1.140306000	-5.440021000	1.056260000
H	0.599766000	-4.972548000	-0.579562000
C	-2.677946000	0.912667000	-3.818022000
H	-1.878834000	0.304242000	-4.239674000
H	-2.272254000	1.893395000	-3.554919000
C	-0.135264000	-7.981050000	0.565269000
H	0.276804000	-8.124231000	1.570683000
H	-0.346022000	-8.963307000	0.134546000
C	-4.554226000	0.427202000	-2.440424000
H	-4.731673000	0.871450000	-1.458252000
H	-5.006573000	-0.566573000	-2.466464000
C	-6.095847000	-2.384436000	-4.698513000
H	-7.028503000	-2.586286000	-5.251634000
H	-6.182598000	-1.404768000	-4.222880000
C	-5.772582000	-3.524115000	-3.731870000
H	-6.673739000	-3.971176000	-3.302690000
H	-5.153980000	-3.153758000	-2.909921000
C	-4.992314000	1.312834000	-3.599797000
H	-6.008867000	1.083077000	-3.929406000
H	-4.956155000	2.366110000	-3.306099000
C	-3.932082000	1.020049000	-4.664308000
H	-3.853444000	1.807882000	-5.417696000
H	-4.143036000	0.070684000	-5.168292000
C	0.159792000	-2.140421000	-3.375299000
H	0.353898000	-3.048886000	-2.798241000
H	0.776878000	-1.320653000	-3.005208000
C	-4.973752000	-4.507824000	-4.615081000
H	-5.541186000	-5.416870000	-4.834335000
H	-4.041113000	-4.809557000	-4.130365000
C	-4.712393000	-3.700439000	-5.903840000
H	-3.680126000	-3.754456000	-6.255696000
H	-5.372679000	-4.039148000	-6.717321000
C	-2.062509000	-2.392759000	-4.157248000
H	-2.672965000	-1.621167000	-4.627066000
H	-2.704039000	-3.100393000	-3.633654000
H	1.533933000	2.140141000	-3.272050000
H	0.451119000	0.420104000	3.287399000

4

Th	1.273580000	1.625604000	0.643691000
Cl	-0.266944000	0.903618000	-1.606648000
Si	1.746217000	5.813004000	1.146955000
Si	-2.295547000	2.052860000	2.129045000
Si	0.666289000	3.617444000	4.253710000
Si	3.928536000	-1.644928000	-1.367662000
Si	-1.267083000	4.431052000	-0.896805000

Si	2.373863000	-1.007342000	2.440761000
Si	5.336629000	1.742061000	1.705814000
Si	3.914745000	2.065104000	-2.197572000
C	3.741792000	3.907247000	-1.805722000
H	3.588663000	4.481594000	-2.725844000
H	4.609309000	4.325036000	-1.290692000
H	2.868146000	4.061967000	-1.164251000
C	2.594476000	1.678400000	-3.505377000
H	2.260825000	0.640213000	-3.467766000
H	2.979193000	1.882350000	-4.510607000
C	5.646292000	1.793138000	-2.934180000
H	6.413333000	2.194037000	-2.265835000
H	5.733317000	2.320264000	-3.891189000
H	5.872393000	0.737944000	-3.106666000
C	2.842476000	-0.315384000	-0.528131000
C	3.502041000	1.075288000	-0.677951000
C	3.236212000	-0.257002000	0.948755000
C	0.881033000	4.166022000	1.226348000
C	4.001229000	0.910198000	0.671730000
C	0.803522000	7.047906000	2.245408000
H	-0.228582000	7.134920000	1.892007000
H	1.261783000	8.042546000	2.207347000
H	0.763949000	6.730674000	3.289236000
C	-2.696157000	0.734853000	0.804772000
H	-2.724122000	1.200992000	-0.180528000
H	-3.650241000	0.236668000	0.998683000
H	-1.894903000	-0.014098000	0.844124000
C	5.707257000	-1.483993000	-0.768904000
H	5.783388000	-1.771492000	0.281723000
H	6.052111000	-0.451540000	-0.864929000
H	6.367391000	-2.126763000	-1.360414000
C	-0.722296000	2.943274000	1.729008000
C	-0.361215000	3.741861000	0.571971000
C	0.518011000	3.343510000	2.417017000
C	3.561600000	5.744044000	1.686883000
H	4.025660000	6.727237000	1.551747000
H	4.108547000	5.026679000	1.068582000
H	3.683272000	5.453405000	2.730287000
C	-2.332435000	1.109777000	3.768714000
H	-1.593787000	0.305807000	3.777295000
H	-3.331285000	0.672355000	3.883085000
H	-2.152405000	1.757511000	4.628862000
C	0.820720000	2.030880000	5.283295000
H	-0.004247000	1.333821000	5.129192000
H	0.847561000	2.290513000	6.347884000
H	1.750072000	1.509935000	5.040362000
C	2.130063000	4.677918000	4.835384000
H	3.090573000	4.179048000	4.696778000
H	1.998070000	4.844945000	5.910876000
H	2.176815000	5.655893000	4.354685000
C	-3.742661000	3.281394000	2.212067000
H	-3.657468000	3.872768000	3.129473000
H	-4.710756000	2.767449000	2.226681000
H	-3.731560000	3.981651000	1.374307000
C	3.234050000	-3.361044000	-0.983551000
H	3.456560000	-3.673402000	0.037557000
H	3.656747000	-4.098202000	-1.675030000
H	2.147387000	-3.351580000	-1.108828000
C	1.789863000	6.645314000	-0.562516000

H	2.469745000	6.150749000	-1.257859000
H	2.164121000	7.664167000	-0.406238000
H	0.811275000	6.725872000	-1.037274000
C	3.886870000	-1.532993000	-3.260976000
H	2.874795000	-1.675159000	-3.648925000
H	4.506608000	-2.345022000	-3.659282000
H	4.270944000	-0.592028000	-3.655814000
C	-0.895232000	4.543858000	4.822499000
H	-1.018948000	5.457950000	4.233960000
H	-0.840762000	4.818781000	5.881782000
H	-1.793068000	3.938010000	4.674616000
C	-2.830545000	3.476078000	-1.393660000
H	-2.582988000	2.451776000	-1.669042000
H	-3.259100000	3.976508000	-2.270502000
H	-3.593831000	3.457209000	-0.613213000
C	2.308533000	-2.898884000	2.344459000
H	1.726668000	-3.218324000	1.478505000
H	1.835879000	-3.286369000	3.253923000
H	3.315940000	-3.323052000	2.274833000
C	-1.909544000	6.172194000	-0.484313000
H	-2.576677000	6.107346000	0.382073000
H	-2.480339000	6.587497000	-1.322912000
H	-1.113230000	6.874908000	-0.232652000
C	0.734410000	-0.135696000	2.360111000
H	1.806248000	-0.524658000	-0.854089000
H	-0.057505000	-0.767985000	1.942586000
C	6.396088000	2.899286000	0.656849000
H	5.819960000	3.728533000	0.242750000
H	6.867040000	2.357918000	-0.167665000
H	7.191283000	3.324841000	1.278267000
C	-0.184610000	4.456947000	-2.455659000
H	0.853294000	4.709936000	-2.232596000
H	-0.559744000	5.178621000	-3.190000000
H	-0.198952000	3.462075000	-2.910622000
C	4.552560000	2.732728000	3.116113000
H	5.125899000	3.643935000	3.309082000
H	4.512986000	2.158137000	4.044312000
H	3.531932000	3.037052000	2.864200000
C	3.298951000	-0.602870000	4.040007000
H	4.249200000	-1.140775000	4.105323000
H	2.678351000	-0.904954000	4.890419000
H	3.500418000	0.463861000	4.140786000
C	6.469503000	0.396601000	2.398262000
H	5.884416000	-0.394647000	2.871754000
H	7.157462000	0.811050000	3.142763000
H	7.061672000	-0.053708000	1.596627000
Cl	-3.720121000	-2.342666000	-0.758887000
Cl	-1.717668000	-5.089723000	-2.252815000
Cl	-0.386942000	-2.332248000	-0.158645000
Mg	-2.087516000	-4.156847000	-0.07328200
Mg	-1.883585000	-0.930713000	-1.48203400
O	-3.809546000	-5.368668000	0.251799000
O	-2.235119000	-3.663706000	2.016811000
O	-0.823146000	-5.727451000	0.633702000
O	-1.256219000	-1.756919000	-3.243314000
O	-3.170532000	0.261799000	-2.619223000
O	-5.120164000	-2.313452000	-5.505431000
C	-5.871337000	-6.113197000	1.101690000
H	-6.714008000	-5.834537000	1.739474000

H	-5.708049000	-7.192488000	1.197561000
C	-2.867400000	-2.466939000	2.519457000
H	-3.907416000	-2.476690000	2.192117000
H	-2.371308000	-1.594925000	2.086614000
C	-4.607186000	-5.346273000	1.451791000
H	-4.835785000	-4.305520000	1.702125000
H	-4.016781000	-5.783510000	2.259357000
C	-2.650116000	-2.536634000	4.016161000
H	-3.331927000	-3.265875000	4.469484000
H	-2.793191000	-1.570993000	4.503135000
C	-6.058614000	-5.727459000	-0.365674000
H	-6.708626000	-6.410664000	-0.917710000
H	-6.472471000	-4.716317000	-0.442934000
C	-1.328176000	-7.079797000	0.576282000
H	-1.951952000	-7.174168000	-0.317971000
H	-1.935712000	-7.245164000	1.469734000
C	-4.631117000	-5.747096000	-0.884367000
H	-4.314331000	-6.743903000	-1.208096000
H	-4.433950000	-5.041110000	-1.688589000
C	0.173846000	-2.421019000	-4.945935000
H	1.025779000	-3.046696000	-5.222565000
H	0.243822000	-1.476438000	-5.496763000
C	-1.203441000	-3.026174000	4.065518000
H	-0.521751000	-2.190181000	3.874431000
H	-0.929159000	-3.486910000	5.017781000
C	0.837199000	-7.089011000	-0.359523000
H	1.891327000	-7.362261000	-0.270151000
H	0.544293000	-7.135110000	-1.412590000
C	-1.143464000	-4.018859000	2.908508000
H	-0.207674000	-3.957625000	2.355592000
H	-1.308927000	-5.053436000	3.222563000
C	-1.174760000	-3.107982000	-5.190937000
H	-1.103825000	-4.170947000	-4.945774000
H	-1.516018000	-3.004451000	-6.224106000
C	0.558390000	-5.698694000	0.184090000
H	1.182548000	-5.451231000	1.049089000
H	0.652037000	-4.915398000	-0.567408000
C	-2.764925000	0.901261000	-3.856839000
H	-1.989709000	0.279276000	-4.302998000
H	-2.339570000	1.880375000	-3.619896000
C	-0.092887000	-7.959274000	0.486330000
H	0.334979000	-8.132087000	1.480441000
H	-0.311680000	-8.928576000	0.031050000
C	-4.592030000	0.448288000	-2.403076000
H	-4.729307000	0.900771000	-1.418336000
H	-5.053737000	-0.541506000	-2.407122000
C	-6.212639000	-2.373048000	-4.602336000
H	-7.149472000	-2.572984000	-5.149057000
H	-6.300008000	-1.399722000	-4.113845000
C	-5.872537000	-3.522626000	-3.654049000
H	-6.763909000	-3.971667000	-3.206662000
H	-5.232035000	-3.159883000	-2.845721000
C	-5.063617000	1.329701000	-3.552125000
H	-6.095328000	1.110453000	-3.838961000
H	-5.001746000	2.384887000	-3.269705000
C	-4.050055000	1.012395000	-4.654223000
H	-3.992564000	1.790830000	-5.419216000
H	-4.290327000	0.059445000	-5.137699000
C	0.114447000	-2.155978000	-3.456040000

H	0.311891000	-3.057351000	-2.869160000
H	0.737967000	-1.333660000	-3.102940000
C	-5.097237000	-4.498953000	-4.565113000
H	-5.682465000	-5.392759000	-4.799640000
H	-4.165904000	-4.827262000	-4.095348000
C	-4.833486000	-3.667713000	-5.838571000
H	-3.799818000	-3.713441000	-6.187428000
H	-5.489539000	-3.993393000	-6.660435000
C	-2.118442000	-2.421803000	-4.202714000
H	-2.748878000	-1.661429000	-4.665023000
H	-2.739106000	-3.132524000	-3.658433000
H	1.717994000	2.310445000	-3.342730000
H	0.416473000	0.302477000	3.305933000

References

(1) Khan, I. A.; Ahuja, H. S.; Bagnall, K. W.; Sinf, L. *Inorg. Synth*. John P. Fackler Jr., Ed.; 1982; Vol. 21, pp 187–190.

(2) Takanashi, K.; Inatomi, A.; Lee, V. Y.; Nakamoto, M.; Ichinohe, M.; Sekiguchi. *Eur J Inorg Chem* **2008**, 1752–1755.

(3) Westland, A. D.; Tarafder, T H. *Inorg. Chem*. **1982**, *21*, 3228.

(4) Clark, R. C.; Reid, J. S. *Acta Crystallogr. A* **1995**, *51*, 887–897.

(5) Dolomanov, O. v.; Bourhis, L. J.; Gildea, R. J.; Howard, J. A. K.; Puschmann, H. *J. Appl. Crystallogr*. **2009**, 42, 339–341.

(6) (a) Becke, A. D. *J. Chem. Phys*. **1993**, *98*, 5648. (b) Burke, K.; Perdew, J. P.; Yang, W. in *Electronic Density Functional Theory: Recent Progress and New Directions*, Eds: J. F. Dobson, G. Vignale, M. P. Das, Plenum, New York, 1998.

(7) (a) Kuechle, W.; Dolg, M.; Stoll, H.; Preuss, H. *J. Chem. Phys*. **1994**, *100*, 7535 (b) Moritz, A.; Cao X.; Dolg, M. *Theor. Chem. Acc*. **2007**, *118*, 845. (c) Hollwarth, A.; Bohme, M.; Dapprich, S.; Ehlers, A. W.; Gobbi, A.; Jonas, V.; Kohler, K. F.; Stegmann, R.; Veldkamp, A.; Frenking G. *J. Chem. Phys*. **1993**, *208*, 237.

(8) (a) Hariharan P. C.; Pople, J. A.; *Theor. Chim. Acta* **1973**, *28*, 213. (b) Hehre, W. J.; Ditchfield R.; Pople, J. A. *J. Chem. Phys*. **1972**, *56*, 2257.

(9) Gaussian 09, Revision D.01: M. J. Frisch, G. W. Trucks, H. B. Schlegel, G. E. Scuseria, M. A. Robb, J. R. Cheesman, G. Scalmani, V. Barone, B. Mennucci, G. A. Petersson, H. Nakatsuji, M. Caricato, X. Li, H. P. Hratchian, A. F. Izmaylov, J. Bloino, G. Zheng, J. L. Sonnenberg, M. Hada, M. Ehara, K. Toyota, R. Fukuda, J. Hasegawa, M. Ishida, T. Nakajima, Y. Honda, O. Kitao, H. Nakai, T. Vreven, J. A., Jr. Montgomery, J. E. Peralta, F. Ogliaro, M. Bearpark, J. J. Heyd, E. Brothers, K. N. Kudin, V. N. Staroverov, R. Kobayashi, J. Normand, K. Raghavachari, J. C. Burant, S. S. Iyengar, J. Tomasi, M. Cossi, N. Rega, M. J. Millam, M. Klene, J. E. Knox, J. B. Cross, V. Bakken, C. Adamo, J. Jaramillo, R. Gomperts, R. E. Stratmann, O. Yazyev, A. J. Austin, R. Cammi, C. Pomelli, J. W. Ochterski, R. L. Martin, K. Morokuma, V. G. Zakrzewski, G. A. Voth, P. Salvador, J. J. Dannenberg, S. Dapprich, A. D. Daniels, O. Farkas, J. B. Foresman, J. V. Ortiz, J. Cioslowski and D. J. Fox, Gaussian Inc., 2009, Wallingford CT.
